# Automated building energy modeling for energy retrofits using a large language model-based multi-agent framework

**DOI:** 10.1016/j.isci.2025.113867

**Published:** 2025-10-25

**Authors:** Jie Lu, Zeyu Zheng, Max Langtry, Monty Jackson, Yang Zhao, Chenxin Feng, Ruqian Zhang, Chaobo Zhang, Jian Zhang, Ruchi Choudhary

**Affiliations:** 1 Institute of Refrigeration and Cryogenics, Zhejiang University, Hangzhou, China; 2 Energy Efficient Cities Initiative, Department of Engineering, University of Cambridge, Cambridge, UK; 3 Key Laboratory of Clean Energy and Carbon Neutrality of Zhejiang Province, Jiaxing Research Institute, Zhejiang University, Jiaxing, China; 4 Department of the Built Environment, Eindhoven University of Technology, Eindhoven, the Netherlands

**Keywords:** Artificial intelligence, Energy engineering

## Abstract

Building energy modeling is critical for retrofit design, but it is labor-intensive. We present Data2BEM (Data to Building Energy Model), a large language model-based multi-agent framework that parses architectural drawings, specifications, and sensor data to automatically generate and calibrate building energy simulations. Applied to an existing University of Cambridge office building, Data2BEM produced a calibrated model meeting industry accuracy benchmarks and enabled the assessment of heat-electrification retrofits. Relative to professional practice, the system reduced total modeling time by over 90% (48 min versus 8–32 h) with minimal human input. The workflow integrates information extraction, model generation, and data-driven calibration, delivering end-to-end automation while accurately reflecting measured performance. These results indicate that large language model-driven multi-agent methods can accelerate retrofit analysis, lowering expertise and time barriers for practitioners and supporting scalable pathways to building-sector decarbonization.

## Highlights

• LLM-based multi-agent system automates end-to-end building energy modeling
• Achieves ASHRAE Guideline 14-level calibration accuracy on a real building
• Cuts modeling time by >90% (48 min vs. 8–32 h) with minimal human intervention
• Electrification (ASHP) cuts annual energy costs by 44% and carbon tax roughly 5-fold

## Graphical abstract

**Figure:**
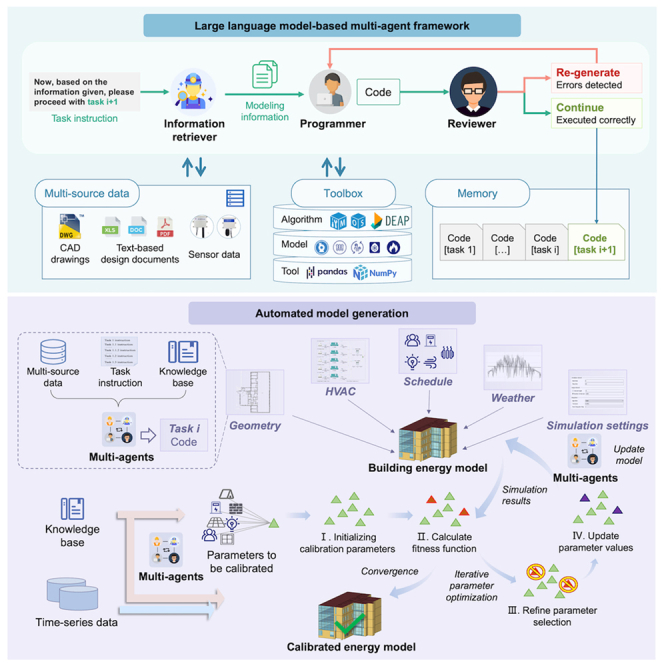

## Introduction

The building sector is one of the world’s largest energy consumers, accounting for approximately one-third of global energy use.^1^ Improving building energy performance through retrofits is crucial for reducing carbon emissions.^2^^,^^3^ Building energy modeling has emerged as a vital technique to represent the physical system.^4^^,^^5^ Such models enable a deeper understanding of energy use patterns and provide actionable guidance for energy-saving interventions. However, developing detailed energy models remains a labor-intensive and technically complex task, involving multiple interdependent stages such as geometry modeling, parameter configuration, model calibration, and retrofit evaluation.

Recent advances in automation technologies have enabled the partial automation of individual modeling tasks. For example, perception-based approaches (such as computer vision,^6^^,^^7^^,^^8^^,^^9^ and 3D sensing via LiDAR or drones^10^^,^^11^^,^^12^) can reconstruct building geometry directly from the physical environment, identifying building components and their topological relationships. Parameter-population approaches^13^^,^^14^^,^^15^ (e.g., rule-based data extraction or template-based tools) can extract and assign model inputs directly from structured design data (e.g., building information modeling or SysML). Optimization-based calibration algorithms can automatically refine parameters to align simulations with real-world performance, thereby supporting retrofit evaluation.^16^^,^^17^^,^^18^ However, these methods function as isolated, single-purpose tools, each addressing only a specific stage of the modeling process, and thus require expert knowledge to manually orchestrate the entire workflow. This is because traditional tools are designed around rigid input-output pipelines with limited contextual awareness. They lack the ability to interpret various unstructured data, understand different user goals, or retain cross-stage dependencies. Consequently, current practices still rely heavily on expert judgment and manual coordination, which limits the scalability and automation of building energy modeling for energy retrofits.

A language-driven modeling framework powered by large language models (LLMs)^19^^,^^20^^,^^21^^,^^22^^,^^23^ enables a fundamentally different paradigm. Unlike task-specific automation techniques, in such an approach, natural language instructions, unstructured design documents, and modeling code can be interpreted and linked within a unified workflow.^20^ Such a system offers the potential to unify the entire modeling workflow within a single, natural language-operable platform, laying the groundwork for autonomous, end-to-end building energy modeling. LLMs act as the central intelligence of an automated modeling system that reads human-oriented design documents and writes machine-executable simulation input files. Prior studies have demonstrated the feasibility of LLM-driven automation for individual modeling tasks. For example, LLMs have been used to convert plain-language building descriptions into the Energyplus model,^24^^,^^25^^,^^26^ extract relevant modeling parameters,^27^^,^^28^ and evaluate energy retrofits.^29^^,^^30^

**Figure 1:**
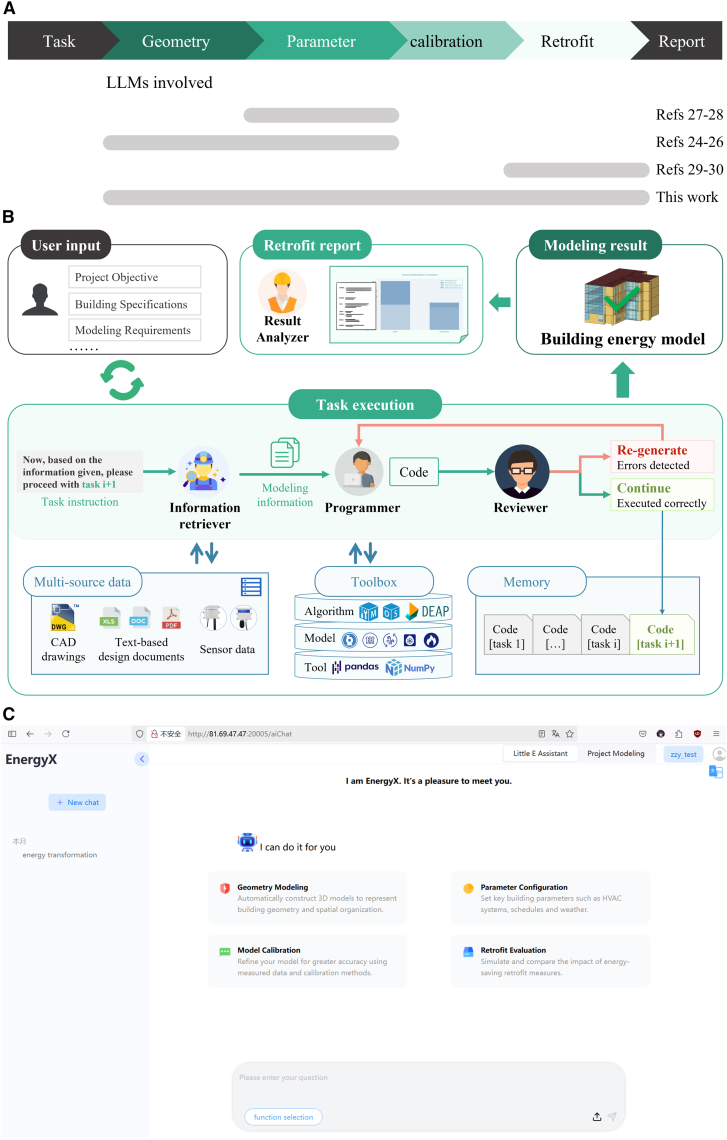
Overview of the LLM-based multi-agent system for automated building energy modeling (A) Task coverage comparison across representative LLM-based frameworks for building energy modeling and analysis (The gray bars denote the stages supported by each method). (B) Architecture of the proposed LLM-based multi-agent system, illustrating how user intent is translated into simulation-ready models. (C) EnergyX: a web-based application interface that enables users to run and visualize the automated modeling process driven by LLM agents.

These pioneering works highlight the potential of LLMs to automate isolated tasks in building energy modeling, such as parameter extraction. However, existing solutions remain fragmented and task-specific. They have not realized the full capabilities of LLM-based agents across the entire modeling pipeline, which starts with heterogeneous building data and ends with retrofit insights (Figure 1A). This fragmentation poses significant barriers to real-world applications. In practice, energy retrofit projects require a seamless flow from data acquisition to model generation, calibration, and scenario evaluation to enable informed decision-making. When tools operate only on isolated stages, practitioners must manually integrate outputs, often relying on expert knowledge to interpret results, resolve inconsistencies, and maintain data continuity across stages. This not only increases labor costs but also introduces risks of human error and delays, undermining the scalability of modeling workflows for large building portfolios. The core challenge in achieving end-to-end automation lies in the demand for structural task planning and verification mechanisms to manage the complexity and interdependencies of multi-stage modeling workflows.

Our proposed platform, Data to Building Energy Model (Data2BEM), addresses the first challenge by introducing a logically structured workflow decomposition (4 stages, 14 tasks, and 32 subtasks). To ensure that each subtask remains within the capability of an autonomous agent team to execute effectively, we define a clear division of labor among four specialized LLM-driven agents (Information Retriever, Programmer, Result Analyzer, and Reviewer, Figures 1B and S1–S4). Each agent is assigned a precise function aligned with domain modeling stages. The Information Retriever parses unstructured inputs (e.g., design documents and user prompts) and outputs structured parameter dictionaries. The Programmer maps these parameters to simulation logic codes. The Result Analyzer interprets simulation outputs and conducts side-by-side comparisons for retrofit evaluation. The Reviewer agent is designed for verification mechanisms to enforce cross-stage consistency, detect potential failure points, and trigger recovery procedures when necessary. This architecture transforms LLM capabilities into a deterministic, verifiable pipeline, where each agent contributes domain-aligned reasoning and interpretable outputs. We apply Data2BEM to a real-world building on the University of Cambridge campus, demonstrating its ability to produce a calibrated model and conduct scenario-based retrofit evaluation (e.g., building heat electrification). While this study represents an early exploration, Data2BEM demonstrates the potential for automated modeling to substantially accelerate energy modeling and retrofit analysis.

## Results

### Large language model-based multi-agent system architecture

With the aim of retrofit analysis, we have decomposed a typical building energy modeling workflow into four major stages: (1) geometry modeling, (2) parameter configuration, (3) model calibration, and (4) retrofit evaluation. Within each stage, we further disaggregated the workflow into 14 tasks and 32 subtasks based on the specific characteristics of the modeling process (see Sections 2.2–2.5 for details). Moreover, we developed a multi-agent framework of LLM-driven intelligent agents to handle the fundamental tasks at each stage (Figure 1B). These agents include Information Retriever, Programmer, Result Analyzer, and Reviewer, each specializing in a distinct role in the workflow. We built the agents on a GPT-4-based LLM to maximize their capacity for context understanding and domain-specific reasoning in building energy systems.^21^ Each agent was pre-prompted with structured instructions to ensure consistent behavior on its designated subtask. Detailed agent design and construction procedures are provided in the STAR Methods and in the Data S1 as the supplemental information.

With this team of LLM-based agents, our framework orchestrates the modeling process in a natural language-driven and modular fashion. Upon receiving the user’s input (e.g., building specifications and modeling requirements), the Information Retriever agent is triggered to extract task-relevant data from heterogeneous sources, such as CAD drawings, text-based design documents, and operational datasets. The Programmer agent then interprets the retrieved information and user instructions, and converts them into executable modeling code, such as generating building geometry in an energy modeling tool and configuring parameters for systems and schedules. The Reviewer agent systematically evaluates both the information retrieved and the modeling code generated by upstream agents. It verifies whether the extracted data correctly reflects the task-specific objectives and whether the generated code conforms to the expected simulation input structure and can execute without errors. This iterative loop (Retriever → Programmer → Reviewer) continues until all stages are completed and a final validated energy model is produced. At the final stage of retrofit evaluation, the Result Analyzer agent is invoked to assess the simulation outputs. It focuses on analyzing retrofit performance and quantifying energy savings by automatically comparing pre- and post-retrofit scenarios. Throughout the process, structured prompts and persistent internal memory enable agents to maintain task context and progressively refine results. The outcome is a coordinated, human-in-the-loop multi-agent workflow (Figure 1B), in which each agent contributes a specific expert capability, including information retrieval, code generation, validation, and result analysis. These agents work collaboratively to construct and evaluate high-fidelity energy models that support retrofit decision-making.

This LLM-based multi-agent approach enables users to generate building energy models primarily based on heterogeneous real-world data sources, supplemented by natural language specifications. After uploading source documents, users are relieved of scripting or technical modeling details, as the agents autonomously generate and refine the simulation-ready model. In addition, the agents are equipped to invoke external simulation engines and libraries (e.g., energy modeling APIs and optimization algorithms) to carry out specialized computations beyond LLM reasoning capabilities, enhancing overall automation. Throughout the modeling process, users remain in a supervisory role to verify final outputs and ensure alignment with practical requirements. To further streamline user interaction and lower adoption barriers, we developed a web-based application interface that integrates the entire multi-agent system (Figure 1C and Video S1). Through this interface, users can upload documents, define modeling requirements via natural language inputs, and monitor task progression and model generation results with relatively fast response times (Data S6 in the supplemental information). This design enables non-expert users to efficiently access the full capabilities of automated building energy modeling without requiring programming expertise.

Video S1. Demo video of the EnergyX platform, related to Figure 1C

To demonstrate the applicability of the proposed methodology in a realistic scenario, we selected a representative case from a building on the University of Cambridge campus. This site features a typical institutional facility with centralized gas boilers, common across higher education and public sector buildings in the UK. Its well-documented operational data, clear heating distribution topology, and standard geometry make it an ideal candidate for testing automated modeling workflows.

### Geometry modeling

**Figure 2:**
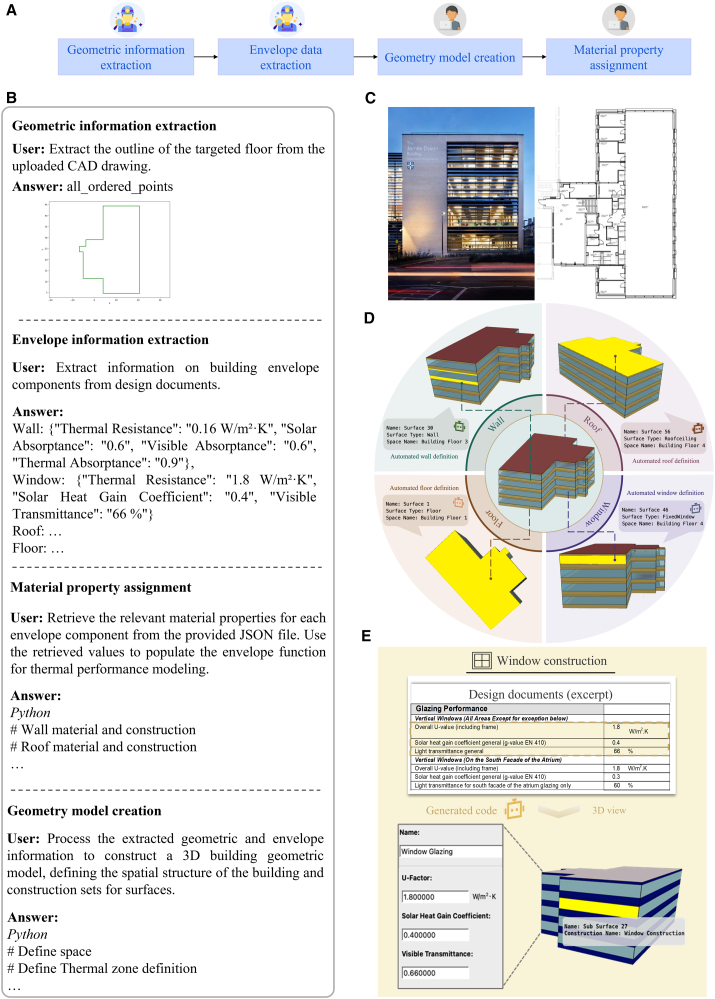
Results of automated geometry modeling (A) Workflow for geometry modeling copiloted by Information Retriever and Programmer agents. (B) Interaction between humans with agents for geometry modeling. The dialogue presented in the figure is simplified for the illustrative purpose (see details in Data S2). (C) The view of the tested building and the first-floor plan. (D) Results of automated geometry modeling, including walls, roof, floor, and windows. (E) Results of automated envelope modeling for window construction.

To generate the building’s geometry automatically, we deployed an LLM-based agent team on the raw CAD drawings, rather than manually recreating the geometry in an energy modeling tool. We designed a structured procedure where the agent sequentially extracts geometric data from the uploaded files and incrementally generates Python code that defines the 3D geometry (surfaces and zones) for the energy model (Figures 2A and S5).

Internally, the geometry modeling task was handled through a collaboration between an information extraction agent and a programmer agent (Figure 2B). Leveraging the structured information embedded in the CAD files, the Information Retriever agent parsed the drawings to identify key geometric features of the building. This process utilized CAD layers and coordinates, such as reading a DXF file with a lightweight Python parser (e.g., ezdxf), to obtain a precise outline of the targeted floor. It distilled this data into a structured description (e.g., a list of corner coordinates for the outline). The Information Retriever agent parsed design documents to identify the thermal and optical properties of the building envelope. Textual design documents specify critical parameters such as thermal transmittance (U-values). From unstructured text and tables, it pulled out key parameters for walls, windows, the roof, and floors (e.g., insulation levels, glazing thermal resistance, solar heat gain coefficient, and surface absorptances). For example, the agent could read that exterior walls were specified to have a thermal transmittance of about 0.16 W/m^2^·K and windows a solar heat gain coefficient of about 0.40, and it compiled these values into a structured JSON output. The programmer agent then translated the extracted geometry description into well-organized Python codes that programmatically construct the building geometry model. In these codes, each floor is represented as a single thermal zone, with its bounding surfaces defined based on the extracted geometry. For example, a closed polyline denoting the outer boundary of a floor plan is converted into a surface set comprising the surrounding walls, a floor slab at the base, and an overhead ceiling or roof. Surface types were automatically differentiated based on layer names and geometric connectivity, distinguishing exterior walls from internal partitions, horizontal floor/ceiling planes from the roof, and identifying transparent elements such as windows where applicable. Finally, the Programmer agent uses the envelope JSON to assign material and construction properties to each surface. It automatically creates material objects (insulation, glazing layers, and so forth) and assigns the exact U-values, SHGCs, and absorptances extracted earlier. Each wall, floor, roof, and window in the model is given the numeric thermal properties from the design documents instead of generic defaults. In this way, the building’s envelope is configured to match the specified performance purely through natural-language guidance. The modeling subtask outputs a modular Python script (e.g., Geometry modeling.py), preserving stepwise transparency and facilitating the downstream simulation configuration. Details about geometry modeling are shown in Data S2 in the supplemental information.

An educational building at the University of Cambridge (UK), constructed in 2016 (Figure 2C), was used to illustrate the automated modeling framework. This building’s primary usage is for research office space. Executing the agent-generated geometry modeling workflow yielded a complete 3D model of the building that aligned with the original CAD design (Figure 2D). The floor plan outline was correctly reproduced: all building surfaces appear in their proper locations and are assigned to the correct building story. Every identified wall, roof, floor, and window was placed and oriented according to the blueprint, without any manual adjustments. Additionally, envelope construction parameters were accurately configured based on the design documentation, ensuring consistency with material specifications and thermal properties (Figure 2E). This output geometry is saved in.osm format, forming the basis for downstream modeling tasks. As demonstrated in this geometry modeling step, the LLM-based agent can accelerate model creation, requiring only minimal human guidance (the CAD file and an instruction). Notably, by directly interfacing with the CAD data, the agent can incorporate design details that would be inaccessible to the natural language description alone (as adopted in previous research), allowing it to handle complex layouts (such as irregularly shaped building footprints) with ease.

### Parameter configuration

To fully automate the setup of a building energy model, the platform employed LLM-based agents to configure all key simulation parameters through four natural language-guided subtasks: (i) HVAC system configuration, (ii) schedule generation, (iii) weather data assignment, and (iv) simulation setting. At this stage, each subtask was handled by a team of agents that interpreted design intent from documents or user-provided descriptions and imputed the corresponding model parameters (Figures S6–S9).

**Figure 3:**
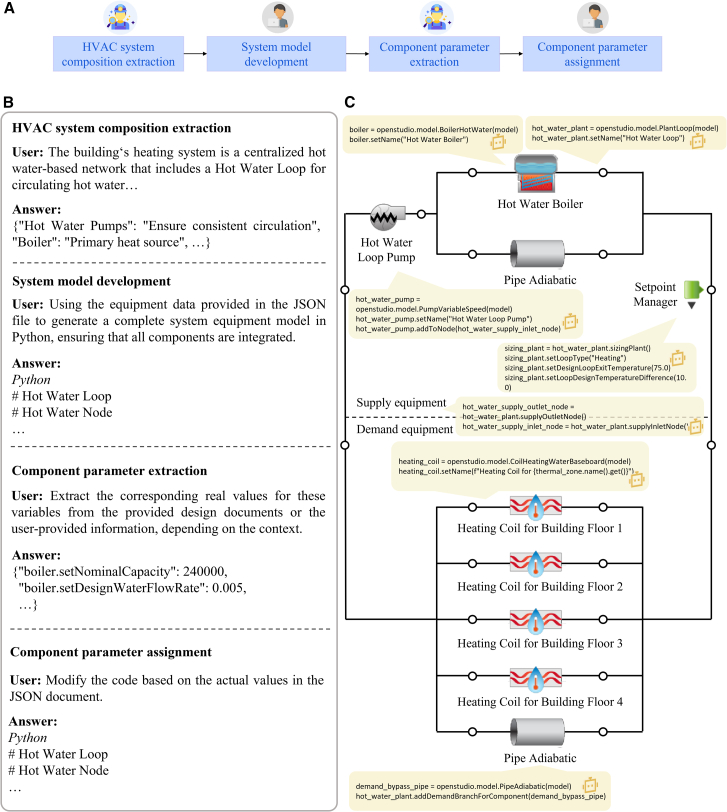
Results of automated heating system modeling (A) Workflow for heating system modeling copiloted by Information Retriever and Programmer agents. (B) Interaction between humans with agents for heating system modeling (see details in Data S3). (C) Heating System constructed in OpenStudio based on code provided by the Programmer agent.

The first subtask focused on configuring the building’s heating, ventilation, and air-conditioning (HVAC) system based on a high-level description (Figure 3A). Starting with a natural language summary of the intended heating system (for instance, “a centralized hot-water loop with a boiler, pumps, bypass pipes, setpoint controls, and radiators for heat delivery”), the Information Retriever agent identified the list of required HVAC components and their relationships (Figure 3B). It produced a structured representation of the system composition, enumerating elements such as the hot water plant loop, circulation pumps, boiler, setpoint manager, supply and demand bypasses, and zone radiators. Given this breakdown, the Programmer agent then assembled the corresponding HVAC model automatically by stitching together model code scripts for each component drawn from a knowledge base of OpenStudio models. For example, creating a PlantLoop for the hot water circuit, adding a BoilerHotWater as the heat source, pumps on the loop inlet, and baseboard radiator coils in each thermal zone. All components are named and linked precisely according to the user’s requirements. This LLM-driven code generation yielded a complete HVAC system model that mirrored the intended design. Next, to ensure the system was not only structurally correct but also quantitatively accurate, a parameter extraction phase was carried out. The Information Retriever agent cross-referenced the placeholders in the generated HVAC code (e.g., “actual numeric value” for boiler capacity or design temperatures) against the project’s design documentation and specifications. It extracted numerical values for each of these performance parameters, such as the boiler’s nominal capacity (around 240 kW in this case), its thermal efficiency, design supply temperature (e.g., 75 °C), loop ΔT, pump flow rates, and radiator capacity factors. All retrieved values were compiled into a JSON file and verified by a Reviewer agent for consistency with the source documents. Finally, the Programmer agent automatically replaced the code placeholders with the real values from the JSON (e.g., inserting the boiler capacity of “240000” W, efficiency of 0.90, and so forth into the script and thus finalized the HVAC configuration in the model. Through this chain of LLM-guided steps, the platform achieved a populated HVAC system ready for simulation, derived entirely from the user’s natural language description and project data (Figure 3C). Details about the HVAC system configuration are shown in Data S3 in the supplemental information.

**Figure 4:**
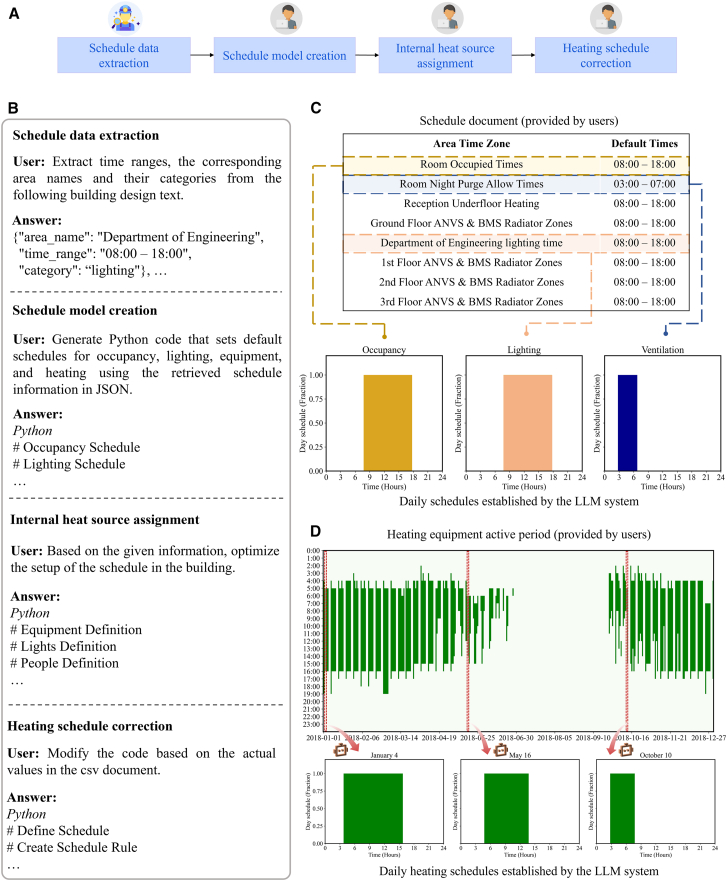
Results of automated schedule modeling (A) Workflow for schedule modeling copiloted by Information Retriever and Programmer agents. (B) Interaction between humans with agents for schedule modeling (see details in Data S3). (C) Extraction of occupancy, lighting, and ventilation schedules from user-provided schedule documents. (D) Derivation of heating schedule from user-provided heating timetables.

The second subtask dealt with operational schedules and internal loads. The system leveraged natural language descriptions of the building’s usage patterns to generate realistic schedule profiles (Figure 4A). The Information Retriever agent first analyzed textual schedule documents to identify typical occupied hours and system operation times for different space types and end-uses (Figure 4B). From these, it extracted time ranges (e.g., “08:00–18:00”) and categorized them into occupancy, lighting, equipment, HVAC heating, and so forth, producing a structured schedule dataset. For instance, it might determine that offices are occupied from 8 a.m. to 6 p.m. on weekdays (with lights and equipment following the same interval), or that heating is active in certain areas during those hours. Using this information, the Programmer agent created corresponding schedule codes in the model. It generated daily schedule profiles for occupancy, lighting, equipment loads, and heating by hard-coding the extracted time intervals into an OpenStudio ScheduleRuleset format (e.g., setting occupancy to 1.0 between 8 a.m. and 6 p.m. and 0.0 otherwise) (Data S3). In parallel, the platform configured the building’s internal heat gains according to design documents. The number of occupants (e.g., 73 people in an office area) and their activity level (metabolic rate of 80 W per person during working hours), lighting power density (e.g., 6.56 W/m^2^), equipment power density (12.34 W/m^2^), and an infiltration rate (say 1.5 air changes per hour with a specific schedule) were taken from the documents. The Programmer agent automatically instantiated People, Lights, and ElectricEquipment objects in each zone with these values, tying their operation to previously generated schedules. For example, people and equipment loads ramp up only from 8 a.m. to 6 p.m., and an infiltration flow is applied from 03:00 to 20:00 each day as specified (Figure 4C). Notably, the heating schedule derived from design intent was further refined using available operational data: in this case, a log of heating “active times” by date was recorded, which the agent analyzed to adjust the heating control schedule dynamically (Figure 4D). By reading a CSV of first-on and last-off times for the heating system, the agent updated the default radiator schedule such that on each day, the heating would start and stop at the times recorded. Through this combination of document extraction and data-driven refinement, the system produced a comprehensive set of building schedules and internal load assignments with minimal manual intervention. Details about schedule generation are shown in Data S3 in the supplemental information.

The third subtask handled the integration of climate data into the model. In our case study, the user simply uploaded a typical meteorological year file for the building’s location (“Weather.epw”), and the Programmer agent incorporated it by setting the weather file path in the model initialization script (replacing a placeholder with the actual file name). The system likewise accepted a design-day (.ddy) file for the location’s peak conditions and inserted it into the simulation settings. In either case, the outcome is that the correct external weather data is attached to the building energy model, ensuring that all subsequent energy simulations run under the proper climate conditions. Details about the weather data assignment are shown in Data S3 in the supplemental information.

**Figure 5:**
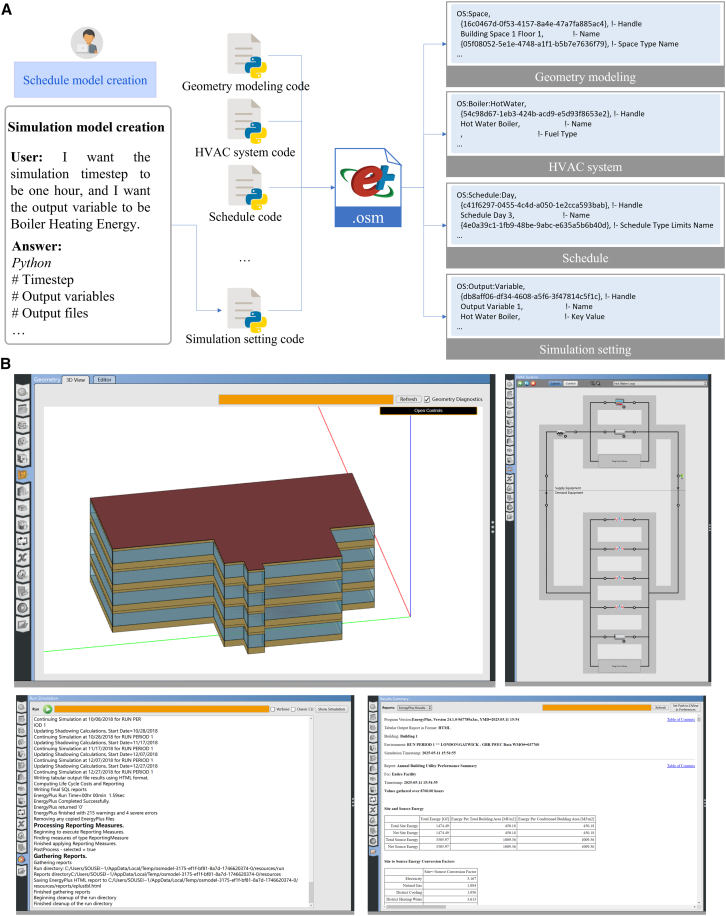
Results of automated simulation settings (A) Generation of simulation setting code by the Programmer agent according to user requirements, and integration into a unified.osm file describing building and simulation parameters (see details in Data S3). (B) Visualization of building and HVAC structures in OpenStudio and execution of simulation using the generated.osm file.

Finally, upon receiving a user’s prompt describing the simulation scenario, the agent parses the request into specific configuration tasks (Figure 5A). For example, given the instruction “I want the simulation timestep to be 1 h, and I want the output variable to be Boiler Heating Energy,” the agent recognizes the need to set a 1-h time step and to monitor boiler heating energy as an output with a reporting frequency of “Timestep.” The new output is bound to the corresponding boiler instance in the model via its key name, so that it measures the boiler’s heating energy usage. If the user had requested a specific simulation period or heating season window, the agent would likewise configure the RunPeriod start and end dates to match those dates, ensuring the simulation only covers the specified months. In the subsequent step, the script previously generated by the Programmer is further processed and integrated into a complete executable file. Running this executable yields an.osm file (OpenStudio file), with its detailed contents presented in Figure 5B (. This resulting building model reflects the geometry, HVAC configurations, schedules, weather conditions, and simulation parameters exactly as requested by the user. Details about the simulation setting are shown in Data S3 in the supplemental information.

### Model calibration

**Figure 6:**
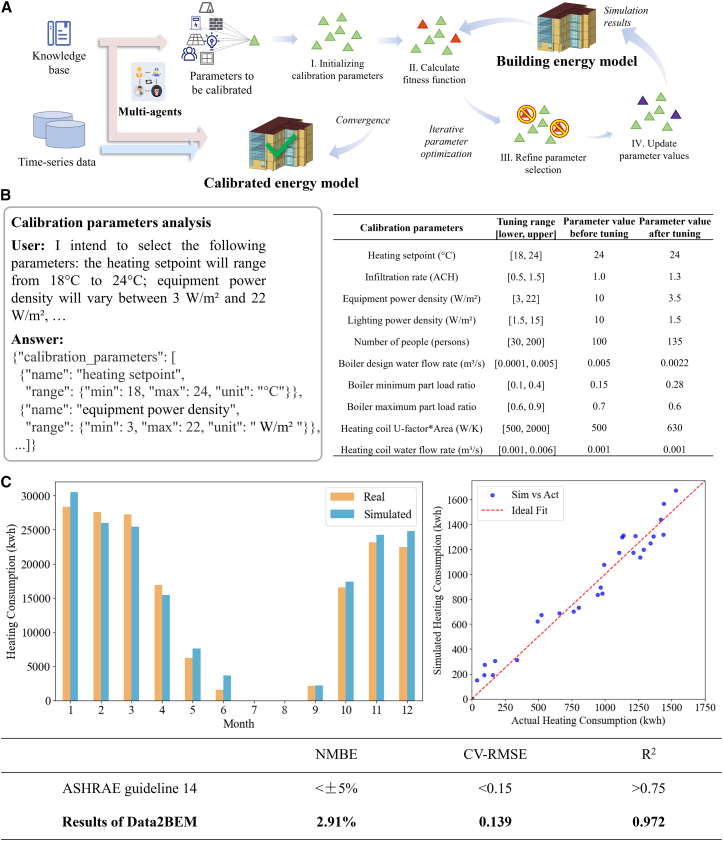
Results of automated model calibration (A) Framework for model calibration using multi-agent collaboration. (B) Interaction between humans with agents for calibration parameter configuration (see details in Data S4), with tabulated ranges and pre- and post-tuning values. (C) Comparison of energy consumption after calibration with real-world values.

To ensure the accuracy of simulation predictions for downstream retrofit evaluations, we developed a natural language-driven, LLM-based multi-agent system to calibrate the building energy model against real-world measurements. This calibration workflow consists of four coordinated subtasks: calibration parameters integration, optimization algorithm configuration, evaluation function definition, and optimization execution. Each subtask is implemented by a team of specialized agents that translate user instructions into simulation-ready code with minimal manual intervention (Figures 6A and S10–S13). In this case, heating energy consumption data from 2018 are used to calibrate the model parameters.

The calibration process begins with the user specifying adjustable parameters via natural language, such as: “heating setpoint from 18°C to 24 °C, equipment power density from 3 to 22 W/m^2^, boiler flow between 0.0001 and 0.005 m^3^/s” (Figure 6B). The Information Retriever agent parses this prompt and compiles a structured JSON file containing all parameters and their ranges (e.g., heating_setpoint, equipment_power_density, lighting_power_density, and boiler_minimum_part_load_ratio). The Reviewer agent then verifies each entry to ensure it faithfully matches user intent. Next, the Programmer agent links each parameter to its corresponding OpenStudio measure in the model library. For instance, AdjustHeatingSetpointMeasure or AdjustBoilerMinimumPartLoadRatioMeasure are identified and loaded into the script using dynamic module paths. The agent then declares the valid numerical ranges for each variable using Python tuples, such as heating_setpoint_range = (18, 24). Note that in this study, operational schedules (e.g., lighting on/off times) were not included in the parameter tuning process, as the focus was placed on physical and equipment-level variables to constrain optimization complexity. Details about calibration parameters integration are shown in Data S4 in the supplemental information.

In the optimization algorithm configuration subtask, the genetic algorithm (GA) is chosen because it is one of the most effective optimization algorithms for model parameter calibration. To search for the optimal parameter set, the Information Retriever extracts the desired GA configuration from natural language: “Use blend crossover with alpha = 0.5, Gaussian mutation (mu = 0, sigma = 1, indpb = 1.0), tournament selection with size 3.” These are compiled into structured JSON and passed to the Programmer, who defines the GA using DEAP’s standard API. Specifically, the agent registers attribute functions for all calibration parameters, constructs the GA individuals using initCycle, and assigns GA operators such as cxBlend and mutGaussian with user-defined hyperparameters. Population size, number of generations, crossover probability (0.5), and mutation probability (0.2) are also defined based on user instructions. Details about optimization algorithm configuration are shown in Data S4 in the supplemental information.

In the evaluation function definition subtask, for calibration evaluation, the user specifies: “Compare the simulated heating energy to real values, and use RMSE as the objective.” The Information Retriever parses and verifies this input, while the Programmer constructs the full evaluation pipeline. A fitness function is defined that reads the measured data and the EnergyPlus simulation output (eplusout.csv), aligns timestamps, and computes the RMSE. Prior to simulation, the agent initializes the model with the ten input variables from each individual, applies the corresponding measure objects to the OpenStudio model, and triggers a simulation run. The complete evaluation logic is encapsulated in an automatically generated script. Details about the evaluation function definition are shown in Data S4 in the supplemental information.

In the optimization algorithm conduction subtask, the GA is executed across 20 generations with a population of 100 individuals. In each generation, offspring are produced via mating and mutation, and then clipped within their valid parameter bounds using the registered ranges. Fitness values are computed using the previously defined evaluation function, and the best-performing individuals are retained. At the end of the evolution process, the best individual’s parameters are recorded, formatted as a string identifier (e.g., hsp_21.4_epd_7.6_ …) and written to best_parameters.txt. The corresponding model is stored in calibrated.osm, ready for downstream use. Details about optimization algorithm conduction are shown in Data S4 in the supplemental information.

Figure 6C presents the calibration results, consisting of three plots. First, it provides an overview by comparing the monthly heating energy consumption between the real data and simulated results. The simulation closely replicates the seasonal heating patterns, with only minor deviations observed in the transitional months. Next, for the test set data, the scatterplots show a strong alignment with the ideal fit line, indicating that the simulated values closely match the actual measurements. Finally, the model’s performance is assessed against the ASHRAE benchmarks, with an NMBE of 2.91%, a CV-RMSE of 0.139, and an R^2^ of 0.972, further confirming the accuracy of the calibrated model in reflecting short-term fluctuations (Data S4).

### Retrofit evaluation

**Figure 7:**
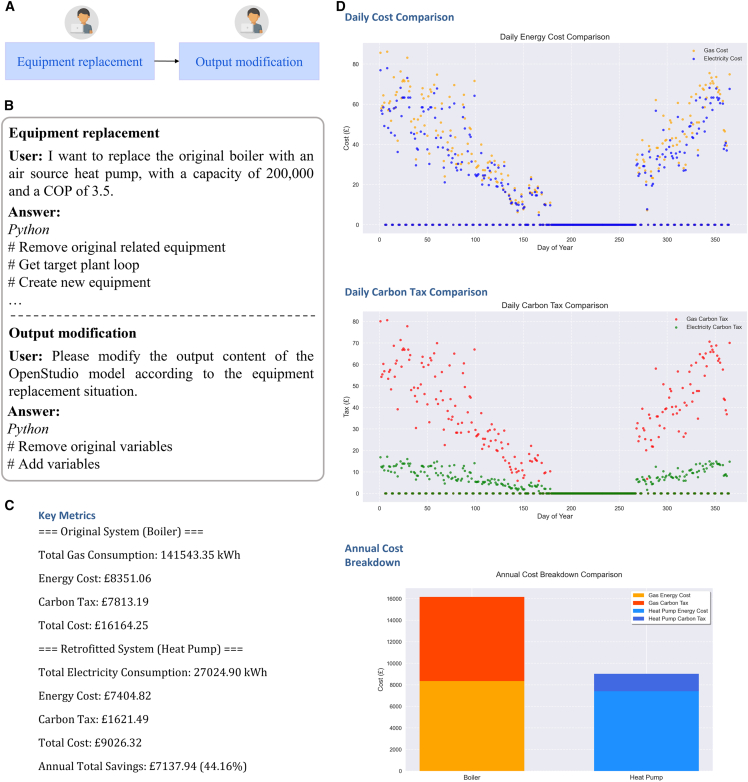
Results of automated retrofit evaluation (A) Workflow for retrofit evaluation copiloted by Programmer and Result Analyzer agents. (B) Interaction between humans with agents for retrofit evaluation. (C) Comparative analysis of pre- and post-Retrofit buildings by the Result Analyzer agent. (D) Visualization of daily cost, daily carbon tax, and annual cost for pre- and post-retrofit buildings by the Result Analyzer agent.

In this study, a representative retrofit scenario is selected to validate the proposed methodology. Specifically, the replacement of gas boilers with air-source heat pumps (ASHPs) exemplifies a widely adopted electrification pathway, frequently emphasized in national and international decarbonization strategies.^31^ To evaluate the performance of energy retrofit scenarios in a calibrated model, we implemented a two-stage workflow (simulation reset and process execution) using our LLM-based multi-agent system (Figure 7A). Given a natural language instruction (e.g., “replace the existing boiler with an air source heat pump of 200 kW and COP 3.5″), the agents collaborated to modify the model, reconfigure outputs, conduct new simulations, and compare results across baseline and retrofit conditions (Figures 7B and S14).

In the simulation reset subtask, the retrofit process began with resetting the HVAC configuration to reflect the intended upgrade. In this case, the Programmer agent responded to the user instruction to replace the original boiler with a high-efficiency ASHP. Following a structured editing routine, the agent removed all existing BoilerHotWater objects from the previous model, validated the presence of the hot water loop, and inserted a new HeatPumpPlantLoopEIRHeating object configured with user-defined parameters: 200,000 W capacity and 3.5 COP. Additional properties, including the minimum part load ratio and sizing factor, were set based on standard defaults, and the ASHP was connected to the supply side of the heating loop. Following the equipment modification, the Programmer agent was prompted to update the simulation outputs. It removed legacy output variables associated with the original boiler (e.g., “Boiler Heating Energy”), and declared new ones for the heat pump, including “Heat Pump Electricity Energy,” bound to the correct device key (“Air Source Heat Pump”) and reporting frequency (“Daily”). All operations adhered to OpenStudio SDK standards and were implemented via agent-generated Python code. Details about the simulation reset are shown in Data S5 in the supplemental information.

In the process execution subtask, with the simulation environment updated, a fresh EnergyPlus was run using the modified.osm model. This execution was performed autonomously, with the run status being monitored and the output files being stored for downstream analysis. After the run, a Results Interpreter agent was activated to conduct side-by-side comparisons of the retrofit and baseline scenarios. Based on the energy usage and cost outputs parsed from simulation files, the agent compiled a detailed summary of annual performance metrics (Figure 7C). The retrofit model using the ASHP showed substantial operational improvements: annual energy cost decreased from £16,164.25 (boiler) to £9,026.32 (ASHP), resulting in yearly savings of £7,137.94 (−44.16%). Similarly, carbon tax obligations dropped from £7,813.19 to £1,621.49—an almost 5-fold reduction (Figures 7D and S13; analysis_report.docx). These results were processed and visualized entirely by the agent through automated CSV parsing and interpretation routines. A downloadable report file summarizing the comparative results was also generated, enabling users to access detailed retrofit insights without manual analysis. Details about the simulation reset are shown in Data S5 in the supplemental information.

Beyond demonstrating the energy-saving potential of retrofits, this case also illustrates a key advantage of the proposed modeling methodology. Unlike traditional workflows, where simulation models are constructed through graphical user interfaces, our method outputs a modular Python code representing the entire energy model. As a result, later-stage interventions, such as replacing HVAC systems, adjusting schedules, or conducting scenario analysis, can be efficiently implemented by simply prompting the LLM agents with natural language instructions. This is because structured code constitutes a native and expressive language for LLMs, allowing them to parse, reason over, and modify simulation logic. This architecture not only reduces manual effort but also enables seamless iteration and adaptation to evolving design goals, making the modeling pipeline inherently more flexible and scalable for real-world applications.

## Discussion

### Time cost comparison

To ensure a fair and reliable benchmarking process, we designed the manual modeling experiment with clear procedures and consistent inputs for all participants. Each modeler received the same complete set of input materials, including the building’s architectural CAD drawings, envelope specifications, HVAC documentation, operational schedules, and other technical references.

The manual modeling workflow was carried out using conventional tools and interfaces without involving any LLM or prompt-based assistance. The process began with geometry reconstruction, in which the participants interpreted the CAD plans and manually redrew the building’s 3D form using SketchUp. This step involved defining spatial zones, floors, and surfaces, requiring careful attention to ensure consistency with architectural intent. Next, participants manually extracted parameter values, such as envelope assemblies, window U-values, HVAC configuration, and occupancy schedules, from supporting documents and configured these within OpenStudio. No automated parsing or generation tools were used. Modelers had to cross-reference source documents and input each parameter manually. Following the initial setup, they engaged in iterative parameter tuning, comparing simulated results with measured data to adjust variables such as internal loads and system efficiencies. This stage was often the most time-consuming and required several trial-and-error cycles using OpenStudio’s built-in features or external calibration plugins. Finally, during the retrofit evaluation stage, modelers manually implemented the retrofit measures by editing the.osm model (e.g., replacing gas boilers with heat pumps) and reran simulations to assess energy savings.

**Figure 8:**
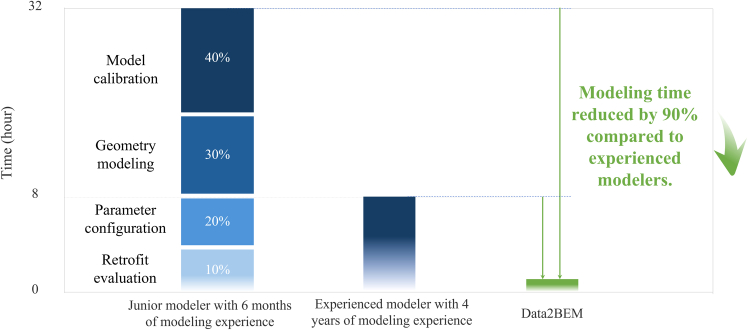
The time cost of modeling for different modelers and Data2BEM

This procedure was repeated by two third-party human participants (someone unfamiliar with the internal workings of Data2BEM) representing realistic proficiency levels in practice: a junior modeler with six months of experience and a senior professional with over four years of hands-on modeling experience. These profiles were selected based on industry observations and reflect common roles in energy modeling and consulting projects. As shown in Figure 8, detailed time logs revealed that approximately 30% of the time was spent on geometry modeling, 20% on parameter configuration, 40% on calibration, and 10% on retrofit evaluation. Overall, the junior modeler required about 32 h to complete the process, while the senior modeler completed it in approximately 8 h. In contrast, the LLM-based multi-agent framework achieved the same end-to-end modeling pipeline in just 48 min with minimal human intervention. This represents a dramatic 90% reduction in total modeling time compared to the expert user.

### Influences of prompt styles on modeling results

The style and phrasing of user prompts significantly influence how LLMs interpret tasks and subsequently represent parameters, particularly reflected in variable naming within its generated modeling code. Differences in terminology, sentence structure, and detail levels provided by users may lead to variations in the symbolic representation of the code. To systematically evaluate the robustness of our model against variations in prompt styles, we conducted extensive experiments where user prompts were intentionally altered in terms of wording, spelling accuracy and added extraneous terms (Data S7).

**Figure 9:**
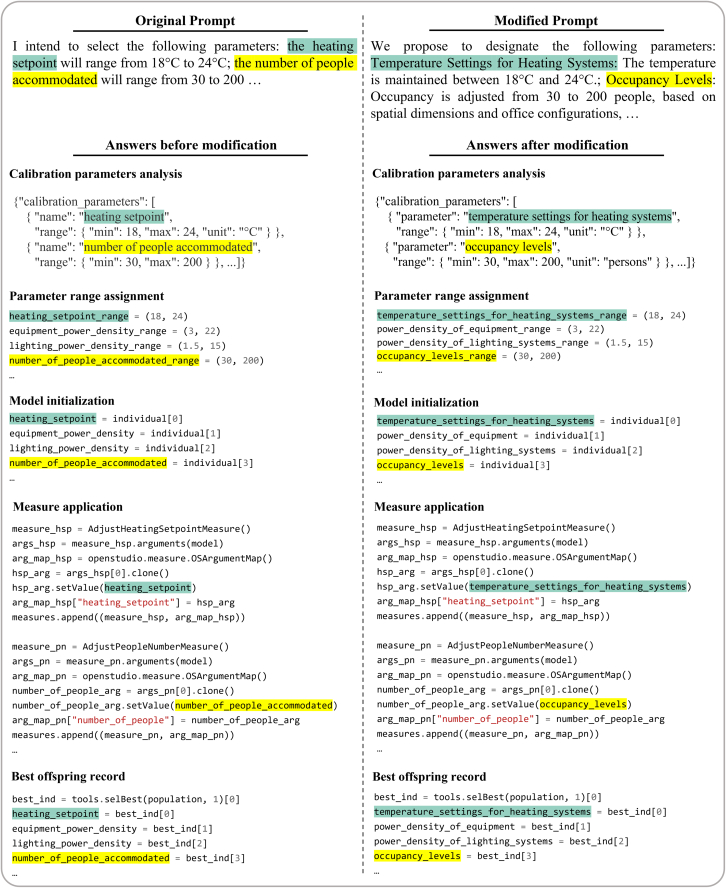
Effect of different prompt styles on calibration parameter extraction and code variable naming

Specifically, our robustness evaluation introduced three types of noise for each initial prompt: altered tone styles, spelling mistakes, and inclusion of unnecessary words. Each prompt was tested multiple times under each noise condition, resulting in an assessment across diverse scenarios. As demonstrated in our results (one case in Figure 9; Table S2, others in Data S7; Figure S15; Table S1), despite these stylistic and linguistic variations, our model consistently produced computationally accurate outputs, maintaining an accuracy rate in meeting specified user requirements.

This evaluation underscores the high robustness and flexibility of our approach, highlighting the model’s capability to interpret and adapt to varied linguistic inputs effectively. These findings further emphasize that prompt styles mainly influence the readability and symbolic clarity of the generated code rather than its underlying computational logic or accuracy.

### Challenge of automated modeling

A core difficulty lies in prompt engineering within a multi-agent system. Unlike single-turn applications, LLM-driven modeling requires chained reasoning across subtasks, where each agent must interpret context, execute domain-specific logic, and produce outputs that downstream agents can reliably process. As such, prompts must be both context-aware and structurally constrained, clearly specifying task objectives, schema formats, naming conventions, and domain assumptions. In practice, developing effective prompts for the Programmer and Reviewer agents proved most demanding. These agents operate at critical transition points, where retrieved information is transformed into executable OpenStudio code and subsequently verified for correctness. For example, the Programmer often requires reference examples with explicitly formatted code blocks to avoid syntax ambiguities or misinterpretation of variable types. Without such a structure, we would observe frequent errors in system object definitions or control logic, which would cascade into downstream failures.

### Limitations of the study

This framework has certain limitations. Firstly, the framework relies on rich data, such as detailed CAD drawings, sensor data, and design documents. Buildings with incomplete or outdated documentation may pose challenges to the framework’s automated data extraction capabilities, potentially requiring manual interventions. Secondly, this study tested the framework within the specific use case of automating energy modeling. The framework has not yet been tested in scenarios requiring more high-resolution modeling, such as precise room-level temperature control or in a simulation setting with links to continuous data and updates from the building. Adapting the framework to such cases will require additional enhancements. Moreover, the validation in this work is limited to a single building case. While sufficient for demonstrating feasibility, one case cannot fully capture the diversity of real-world buildings. Broader evaluations across different building types, scales, and data qualities will be essential to further substantiate the generalizability of the framework. Finally, the current framework employs the commonly used algorithm GA for model calibration. This approach may not be optimal for all calibration scenarios. Incorporating alternative methods, more suited to continuous calibration, could further enhance the framework’s flexibility and performance.

Several promising research directions can be pursued to enhance the functionality, scalability, and real-world impact of LLM-based automated building energy modeling frameworks. First, the proposed Data2BEM platform holds potential to assist retrofit decision-making. Future work should investigate how the system performs on more complex building types, such as multi-use facilities, high-rise structures with mixed HVAC zones, and buildings with integrated renewable energy or energy storage systems. Second, improving the information extraction capability of LLMs remains a critical area of advancement. Future work may focus on enhancing its ability to handle more complex data sources, such as scanned drawings or handwritten sensor logs. Third, an exciting direction lies in leveraging LLMs for real-time calibration and digital twin adaptation. Currently, the system supports offline model tuning using historical operational data. While the calibrated model achieves acceptable accuracy according to ASHRAE Guideline 14 metrics, some discrepancies remain between simulated and measured data, particularly during peak-load periods. These factors introduce inevitable uncertainty, which future work may address by incorporating real-time sensor feedback, refining data extraction mechanisms, and extending the framework to support schedule optimization based on empirical patterns or adaptive inference.

## Resource availability

### Lead contact

Requests for further information and resources should be directed to and will be fulfilled by the lead contact, Yang Zhao (youngzhao@zju.edu.cn).

### Materials availability

This study did not generate new unique materials.

### Data and code availability

• The building operational and design data reported in this study cannot be deposited in a public repository because they contain sensitive information related to building security and privacy.
• This article reports the original code, which has been made publicly available via DOI at https://doi.org/10.5281/zenodo.17374005.
• Any additional information required to reanalyze the data reported in this article is available from the lead contact upon request.

## Acknowledgments

This work is supported by the 10.13039/501100001809National Natural Science Foundation of China (No. 52161135202), the ‘Pioneer’ and ‘Leading Goose’ R&D Program of Zhejiang (No. 2025C01059), the Hangzhou Key Scientific Research Plan Project (No. 2023SZD0028), the Basic Research Funds for the Central Government 'Innovative Team of Zhejiang University' (No. 2022FZZX01-09), and China Scholarship Fund. Max Langtry and Monty Jackson are supported by the 10.13039/501100000266Engineering and Physical Sciences Research Council, through the CDT in Future Infrastructure and Built Environment: Resilience in a Changing World, Grant [EP/S02302X/1].

## Author contributions

Conceptualization, J.L.; methodology, J.L., Z.-Y.Z., J.Z., and R.C.; software, Z.-Y.Z., M.L., M.J., C.-X.F., and R.-Q.Z.; validation, Z.-Y.Z., M.L., M.J., C.-X.F., and R.-Q.Z.; formal analysis, J.L.; investigation, J.L. and C.-B.Z.; writing – original draft, J.L.; writing – review and editing, J.L., Z.-Y.Z., M.L., C.-B.Z., J.Z., Y.Z., and R.C.; visualization, M.J.; supervision, Y.Z. and R.C.

## Declaration of interests

The authors declare no competing interests.

## STAR★Methods

### Key resources table

**Table:** 

REAGENT or RESOURCE	SOURCE	IDENTIFIER
**Software and algorithms**		

Vue.js version 3.5	Evan You	https://vuejs.org/
Python version 3.12.7	Python Software Foundation	https://www.python.org/
EnergyPlus	U.S. Department of Energy’s Building Technologies Office	https://energyplus.net/
Data2BEM Python scripts	This paper	https://doi.org/10.5281/zenodo.17374005
Genetic Algorithm	Holland, J.H.^32^	ISBN: 9780262581110

### Experimental model and study participant details

This study does not involve experimental models or study participants typical in the life sciences.

### Method details

**Figure 10:**
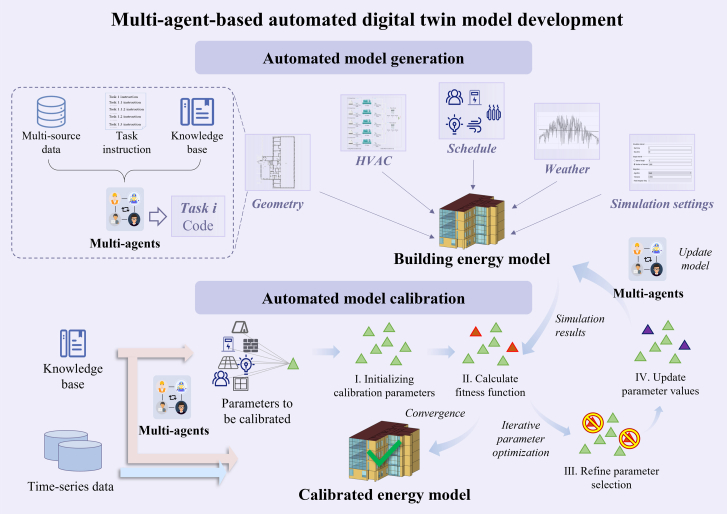
Illustration of the LLM based multi-agent framework for automated modeling

This section introduces the proposed LLM-based multi-agent framework for end-to-end automated modeling of building energy systems (Figure 10). This includes the workflow of tasks for creating and calibrating the energy model.

#### Multi-agent roles and functions

For each modeling subtask, the process can be divided into four sequential steps: instruction generation, extraction of modeling information from multi-source data, code generation, and result review. Accordingly, four specialized agents are defined: Information Retriever, Programmer, Result Analyzer, and Reviewer. Each agent is assigned a distinct role, focusing on a specific step of the task. Working collaboratively, these agents enable the framework to efficiently handle the complex tasks required for modeling.

The information retriever is responsible for searching for and extracting necessary data from multiple information sources, such as CAD files, text-based design documents, and sensor data. This agent ensures that all required information is gathered to support model building. The programmer responsible for generating the initial code for model creation and calibration, and converting the retrieved information into suitable modeling code. The result analyzer generates Python scripts that systematically evaluate and compare performance between original and updated models. This agent extracts essential metrics, conducts comparative analyses, and automatically generates structured summary reports. The reviewer critically evaluates generated outputs against original source materials, assigning confidence scores to indicate their accuracy and consistency. If inaccuracies or unsupported claims occur, the reviewer provides clear feedback and justifications to correct and refine the outputs. Details about agents are shown in Data S1 in supplemental information.

#### Multi-source data and knowledge base for automated modeling

**Figure 11:**
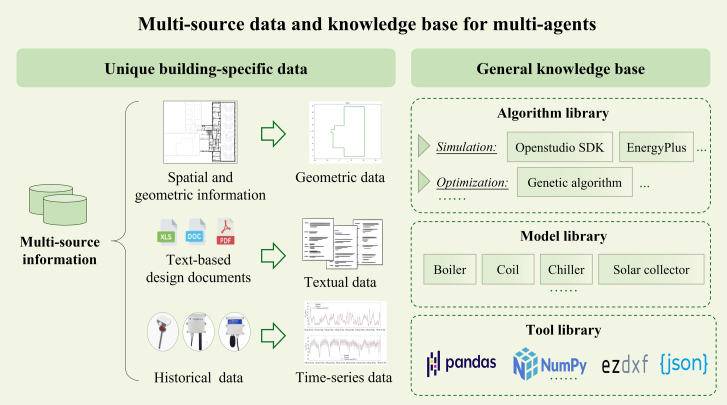
Multi-source data and knowledge base for multi-agents

The automation of modeling relies on the integration of multi-source data and a structured knowledge base, as shown in Figure 11. Multi-source data provides essential information about a building’s spatial configuration, operational characteristics, and historical performance, while the knowledge base consists of computational tools, predefined models, and data processing libraries that support the automation process. The combination of these elements enables the multi-agent system to generate accurate and efficient building energy models with minimal human intervention.

#### Multi-source building-specific data

Three key categories of building-specific data are currently used: spatial and geometric information , text-based design documents and historical data. Future versions could include other forms of data, such as images, BIM files, videos etc.

• Spatial and geometric information: This includes information about the building's physical layout, extracted from various sources such as CAD drawings, BIM models, or point cloud data. Key elements include floor plans, wall positions, room dimensions, window placements, and spatial relationships between components.
• Text-based design documents: Text-based design documents provide essential functional and operational details about the building. Based on the specific needs of the model, different types of information can be extracted, such as material properties, system settings, and usage schedules.
• Historical data: sensor data provides historical measurements of the building operational conditions, such as indoor temperatures, air quality, and energy consumption. Depending on the modeling objectives, historical data can be processed at various levels of resolution, from overall energy use to time-series records. This information supports the calibration of the energy model so that it reflects its real-world operation.

#### Knowledge base

The knowledge base consists of high-level tool functions encapsulated within foundational modeling Application Programming Interfaces (APIs). These APIs provide integration between the agents and the tools required for the energy model. The knowledge base is structured into three core libraries: the algorithm library, the model library, and the tool library.

• Algorithm library: algorithm library contains the vital computational algorithms that drive simulations and optimizations within the modeling framework. For example, for the illustrative case presented in Section 2, the simulation algorithm is the OpenStudio software development kit (SDK). OpenStudio SDK provides an API to access the EnergyPlus modeling engine, enabling integration with LLMs like GPT to perform simulations in various software environments. For calibration, we use an GA algorithm to fine-tune the model parameters.
• Model library: model library consists of two key components. The first component includes pre-defined equipment models for building energy systems, providing ready-to-use representations of typical building energy components, including boilers, chillers and renewable energy modules. The parameters of these models can be customized to reflect the unique energy characteristics of different buildings. The second component comprises modification scripts such as OpenStudio measures. They are scripts designed to automate modifications to model parameters. Each script represents a single adjustment action, such as reducing lighting power density. Users can access hundreds of pre-written measures from the publicly available database like OpenStudio database, or create and add custom measures to meet specific project needs. This library allows LLMs to efficiently simulate various real-world energy systems tailored to specific user requirements, supporting accurate customization and scalability in energy modeling.
• Tool library: Tool library provides predefined functions for data processing and extraction to manage diverse data sources. It includes widely used libraries for data handling, such as Pandas and NumPy, along with specific tools like ezdxf for extracting CAD information and JSON for structuring data. LLMs can directly access these predefined functions, eliminating the need to generate code from scratch, which minimizes potential coding errors and enhances the reliability and accuracy of the results. These tools enable smooth data preparation, integration, and transformation, ensuring complex datasets are efficiently processed.

This knowledge base is fully extendable. For example, users can add custom models or algorithms to the model libraries and algorithm libraries based on specific project needs. This ensures that the framework can efficiently handle diverse modeling requirements, from geometry to HVAC systems, schedules, and weather data.

#### Evaluation method of calibration results

The calibration results are evaluated against ASHRAE Guideline 14 benchmarks. ASHRAE recommends the following statistical indices as criteria for evaluating model accuracy: Normalized Mean Bias Error (NMBE), Coefficient of Variation of the Root Mean Square Error (CV-RMSE), and Coefficient of Determination (R^2^) . The calculation formulars for the se statistical indices are as follows:

$$\text{NMBE}\left(\%\right)=\frac{\sum_{i=1}^{n}\left(M_{i}-S_{i}\right)}{n\times \bar{S}}\times 100\%$$

$$\text{CV}-\text{RMSE}=\frac{\sqrt{\frac{1}{n}\sum_{i=1}^{n}{\left(M_{i}-S_{i}\right)}^{2}}}{\bar{S}}$$

$$R^{2}=1-\frac{\sum_{i=1}^{n}{\left(S_{i}-M_{i}\right)}^{2}}{\sum_{i=1}^{n}{\left(S_{i}-\bar{S}\right)}^{2}}$$

where *M*_*i*_ is the model prediction at time *i*, *S*_*i*_ is the measured value at time *i*, *n* is the total number of data points, and $\bar{S}$ is the mean of the measured values.

#### Construction of the web application

The frontend of the web application was developed using the Vue.js framework, which provides a responsive client-side interface for user interaction (Video S1). Vue.js was responsible for rendering the user interface, capturing user inputs, and communicating with the backend via RESTful API requests. The backend was implemented using Python's Flask framework, which exposed a set of API endpoints to handle various functional requests from the frontend. When users interact with the application, the frontend sends requests to the corresponding API routes, and the backend processes these requests and returns the results to the frontend for display. This architecture ensures a clear separation between the client and server, supports modular development, and enables efficient real-time communication between user operations and backend computation.
