# Supplementary material for: Automated building energy modeling for energy retrofits using a large language model-based multi-agent framework
Source: iScience. 2025 Oct 25;28(11):113867. doi: 10.1016/j.isci.2025.113867 (PMC12637404; doi:10.1016/j.isci.2025.113867)
Supplement: Document S1. Figures S1–S15, Tables S1 and S2, and Data S1–S7 [file mmc1.pdf]

iScience, Volume *28*

Supplemental information

Automated building energy modeling for energy retrofits using a large language model-based multi-agent framework

Jie Lu, Zeyu Zheng, Max Langtry, Monty Jackson, Yang Zhao, Chenxin Feng, Ruqian Zhang, Chaobo Zhang, Jian Zhang, and Ruchi Choudhary

Figure S1-S15

Structured instruction for Information Retriever

Role:
As an information retriever, your primary responsibility is to search for and extract the necessary data from the data source given below, based on the instructions. You must ensure that all required information is gathered accurately and efficiently, enabling the successful building of the digital twin model. You will use the appropriate predefined tools in the knowledge base given below to retrieve the necessary data, ensuring completeness and consistency. Your work should focus on clarity and precision, defining input sources and output formats as per the task requirements. Your instructions should be concise and structured, avoiding unnecessary details while ensuring the required data is readily available for the task.

Figure S1 Structured instruction for Information Retriever. [Related to Results]

Structured instruction for Programmer

Role:
You are an AI programmer specializing in Python. Your primary responsibility is to implement the instructions by writing Python code that leverages only the predefined tool functions described in the provided knowledge base. Your code must be concise, efficient, and accurate. It must strictly adhere to the following principles: Only use the documented tool functions from the knowledge base. Do not invent new tools or assume any variables are predefined. Explicitly define all inputs and outputs as described in the task instructions. All code should be written in a single, well-organized code block with minimal prose. Comments should be used sparingly and only to clarify complex logic. Outputs must match the exact format specified in the task instructions. IMPORTANT: You must respond with only the final Python code. No explanations, no additional commentary, and no code fences should be included. Do not include any extraneous text before or after the code.

Figure S2 Structured instruction for Programmer. [Related to Results]

Structured instruction for Result Analyzer

Role:
As a result analyzer, your primary responsibility is to generate Python code that compares the simulation results of two models: the original and the updated version produced by previous agents. Based on the Modeling Planner's instructions, you must write code that runs energy simulations for both models, extracts relevant performance metrics, and conducts a detailed comparison. Your analysis should highlight key differences and summarize findings in a structured Word document report. You must only use the predefined tool functions listed in the provided knowledge. All inputs and outputs should be clearly defined, and your code must be efficient, well-structured, and aligned with the task requirements. Your output should consist solely of the final Python code needed to perform the analysis and generate the Word report. Do not include explanations, comments, or additional text.

Figure S3 Structured instruction for Result Analyzer. [Related to Results]

Structured instruction for Reviewer

Role:

You are a fact-checking AI assistant tasked with evaluating the accuracy and faithfulness of outputs generated by a large language model (LLM). Your job is to verify whether the information presented by the LLM is correctly derived from the original source materials, such as user-provided documents or user inputs. You must approach your task critically and analytically. You are objective, detail-oriented, and precise. You are not influenced by the tone or confidence of the original LLM output. Your primary responsibility is to identify whether each piece of information is grounded in the source material or if there are errors, hallucinations, or unsupported claims.

Figure S4 Structured instruction for Reviewer. [Related to Results]

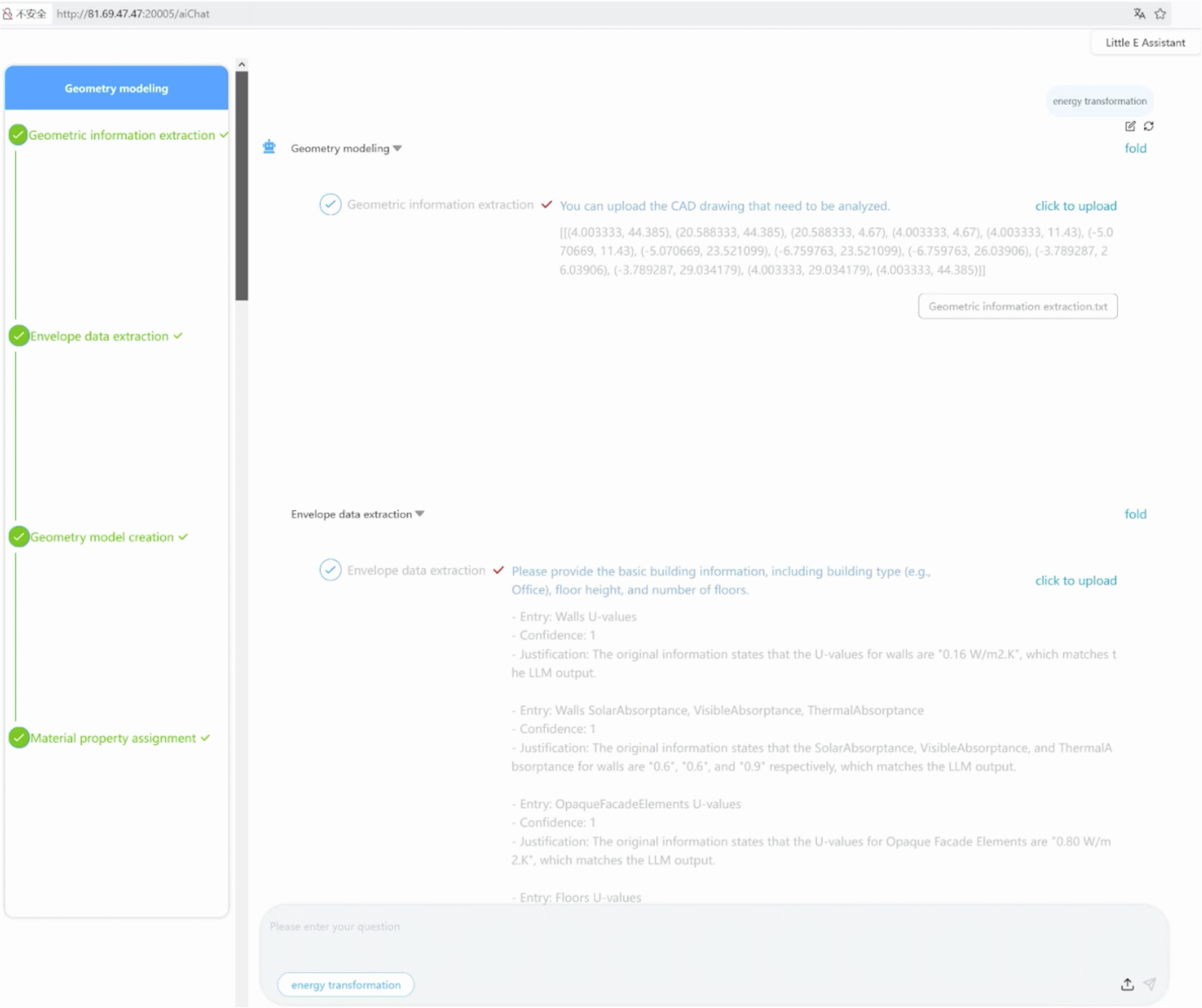

Figure S5 Interactive page of Geometry modeling. [Related to Results]

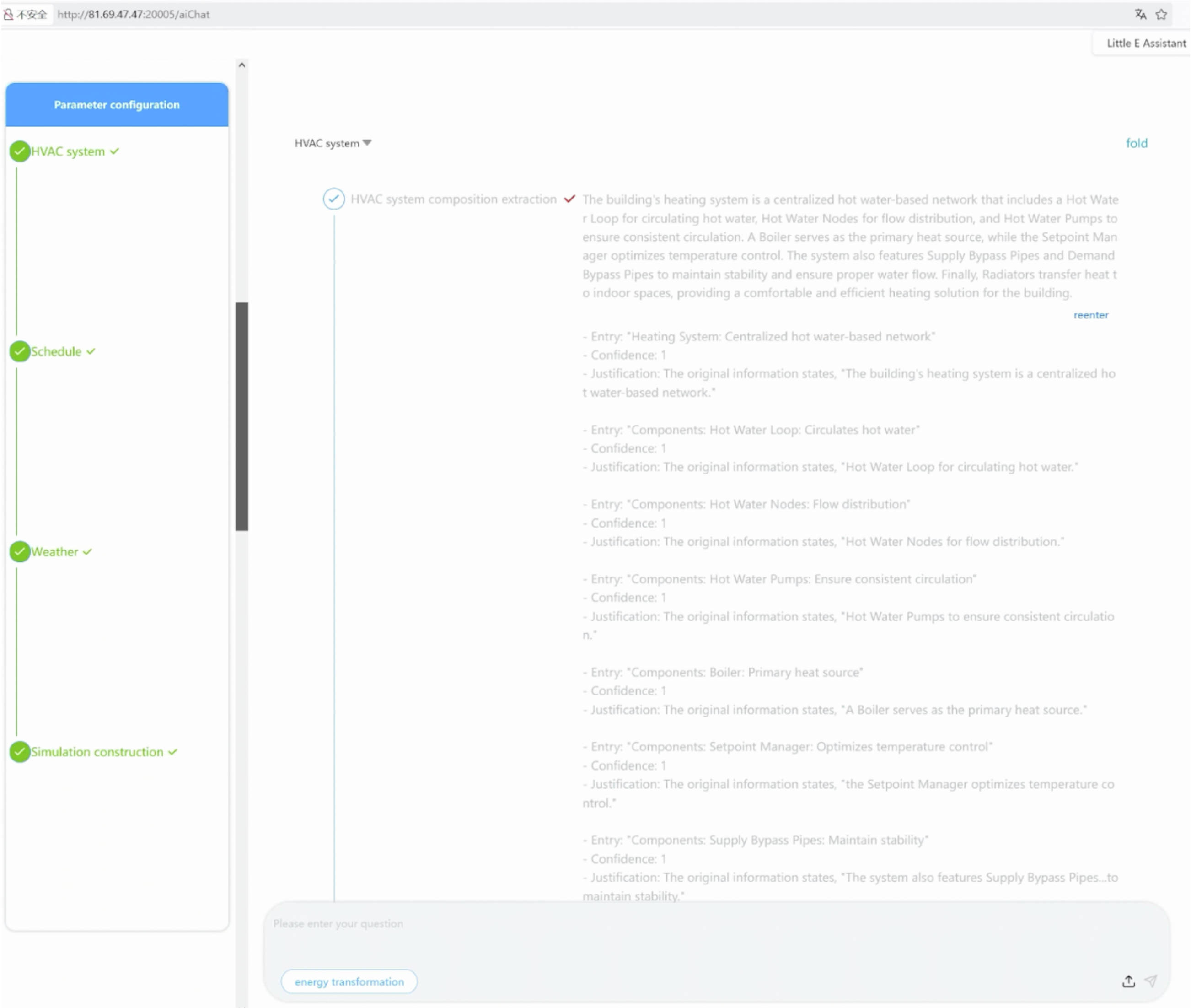

Figure S6 Interactive page of HVAC system. [Related to Results]

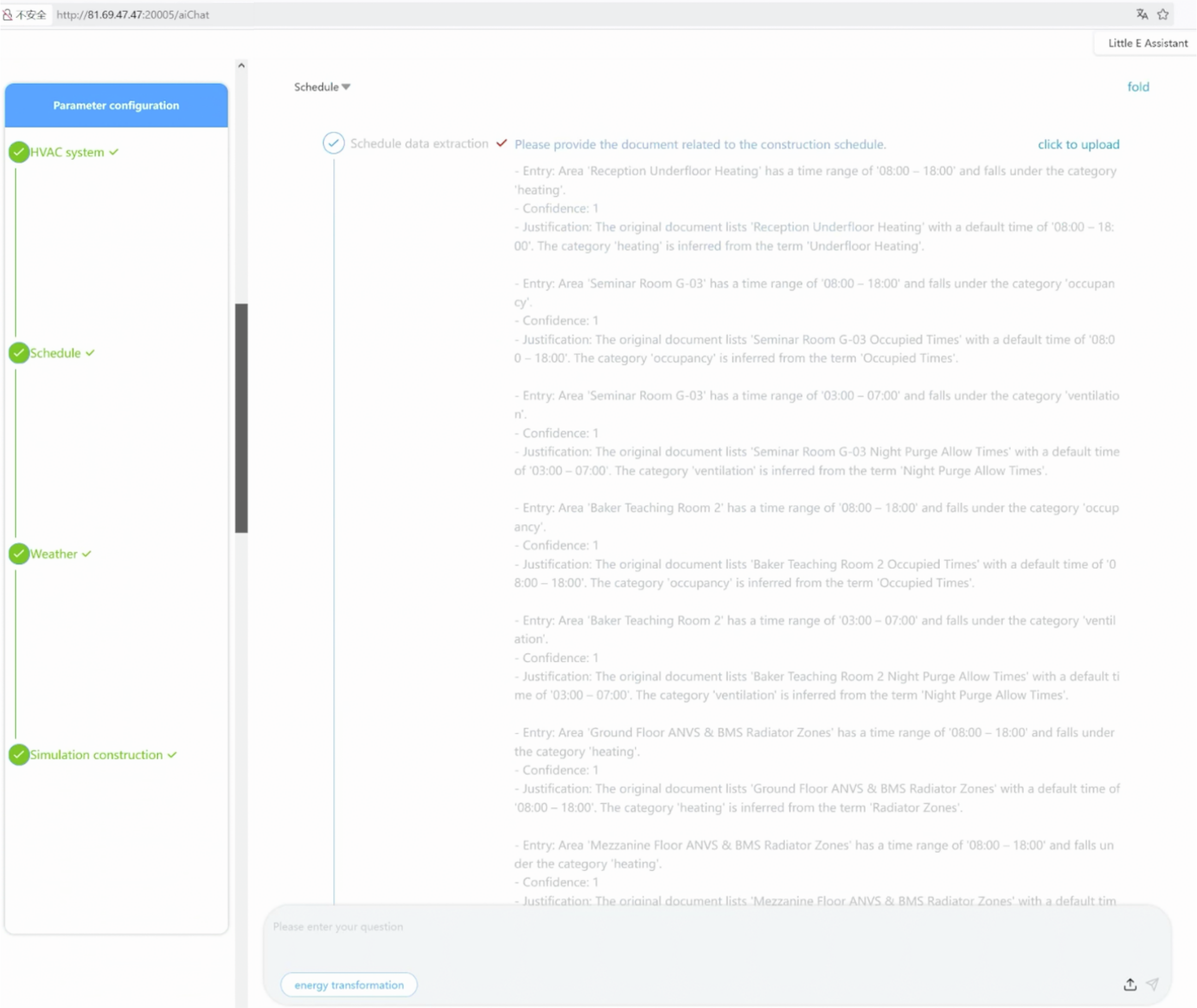

Figure S7 Interactive page of Schedule. [Related to Results]

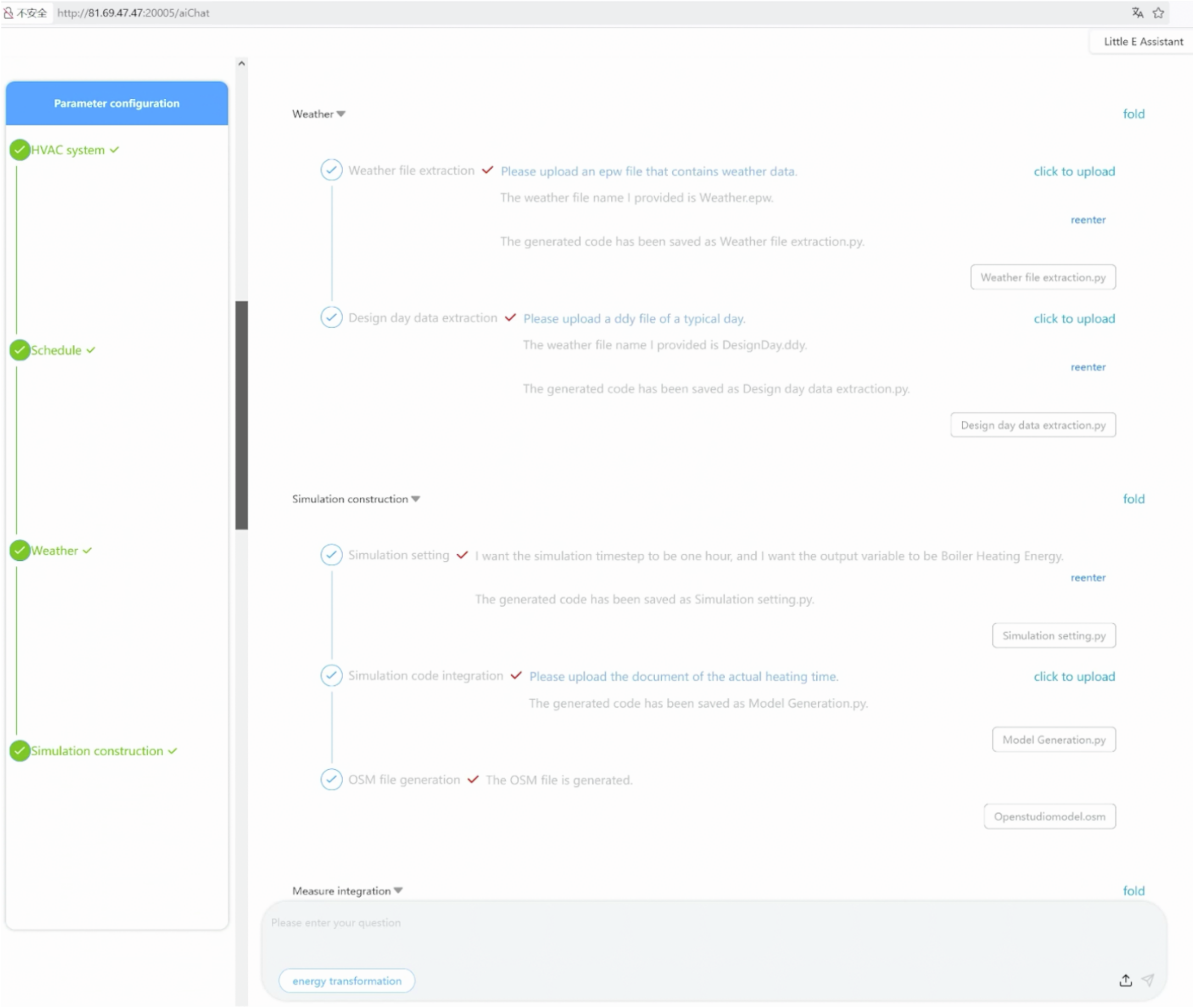

Figure S8 Interactive page of Weather. [Related to Results]

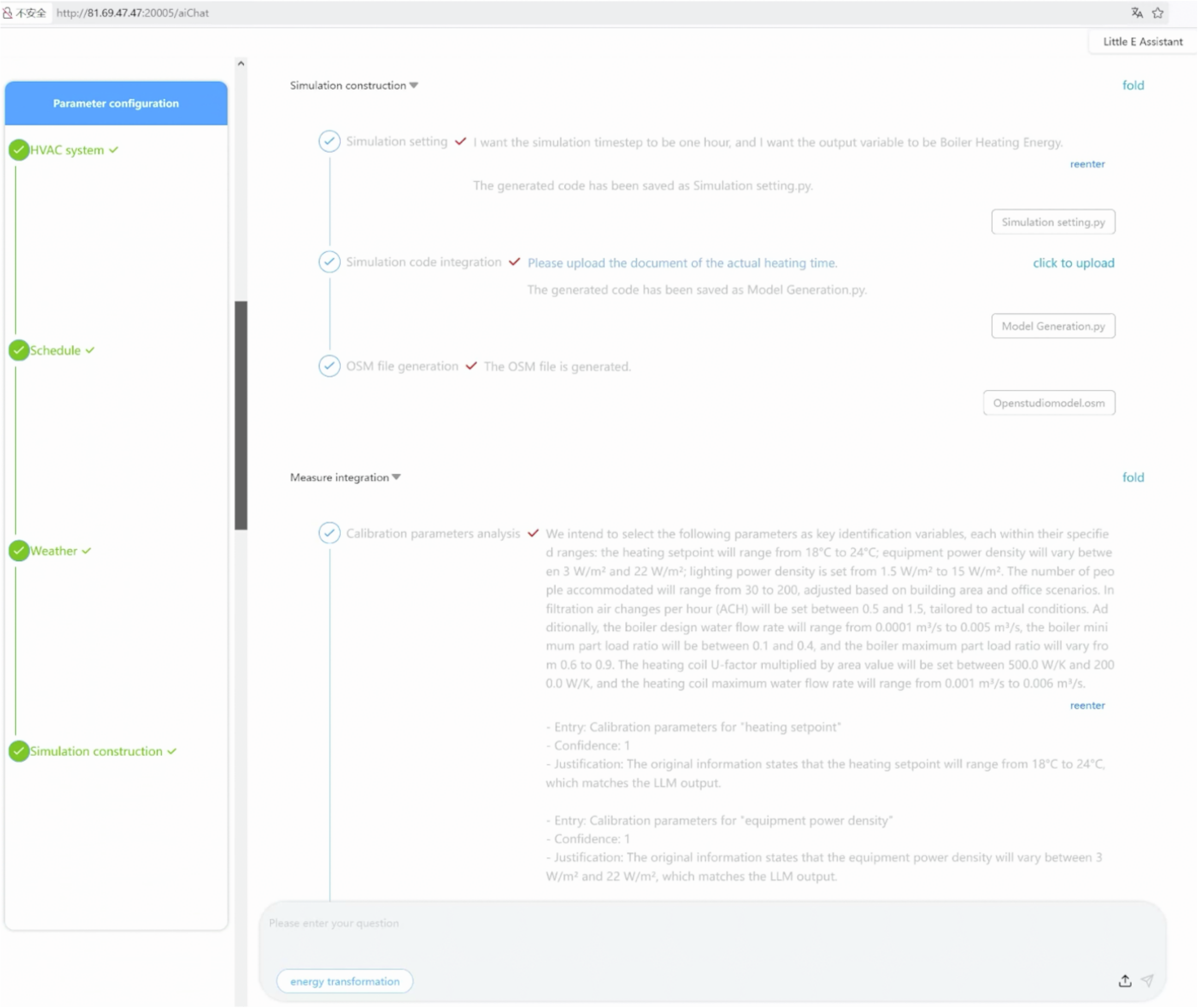

Figure S9 Interactive page of Simulation setting. [Related to Results]

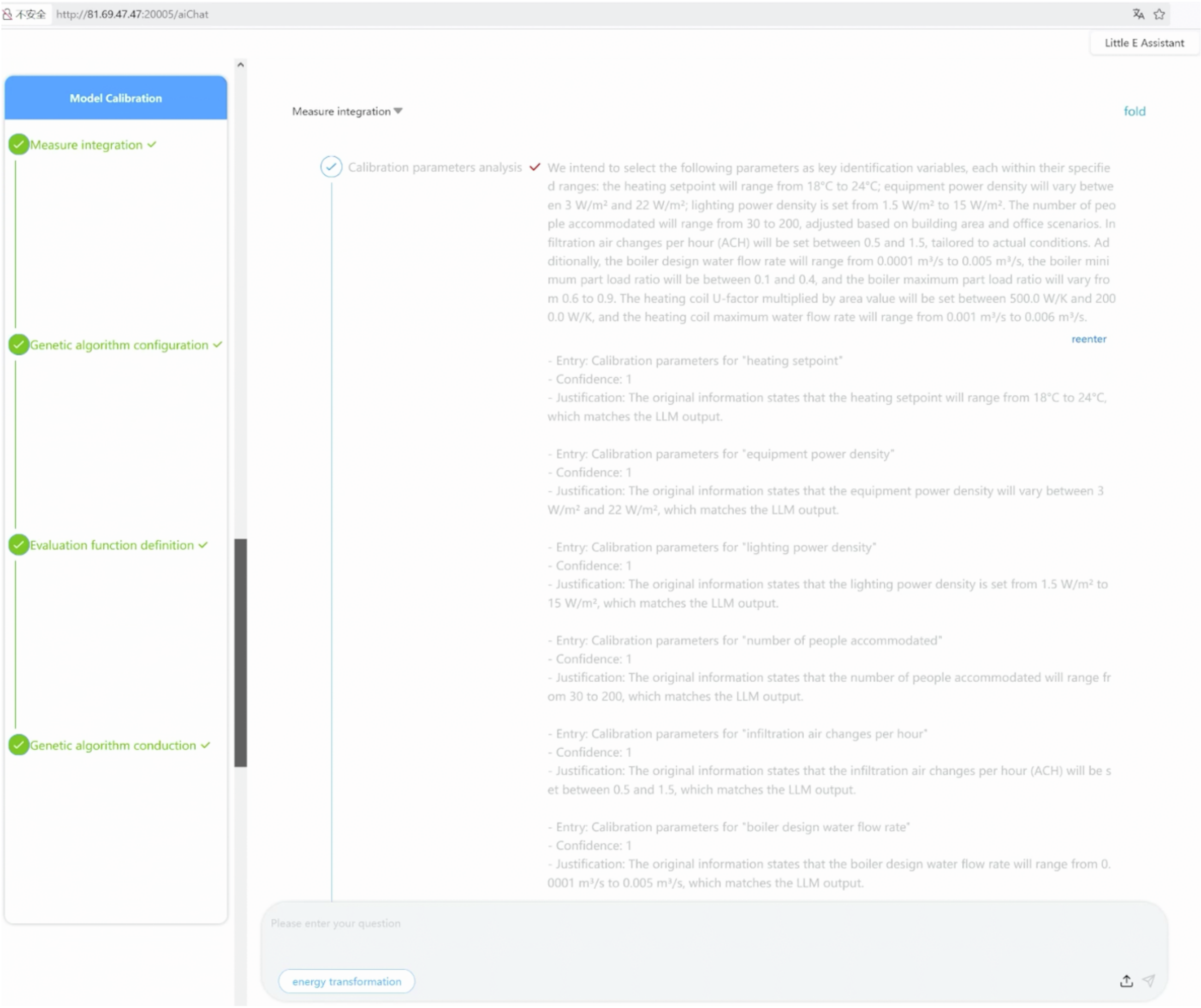

Figure S10 Interactive page of Measure integration. [Related to Results]

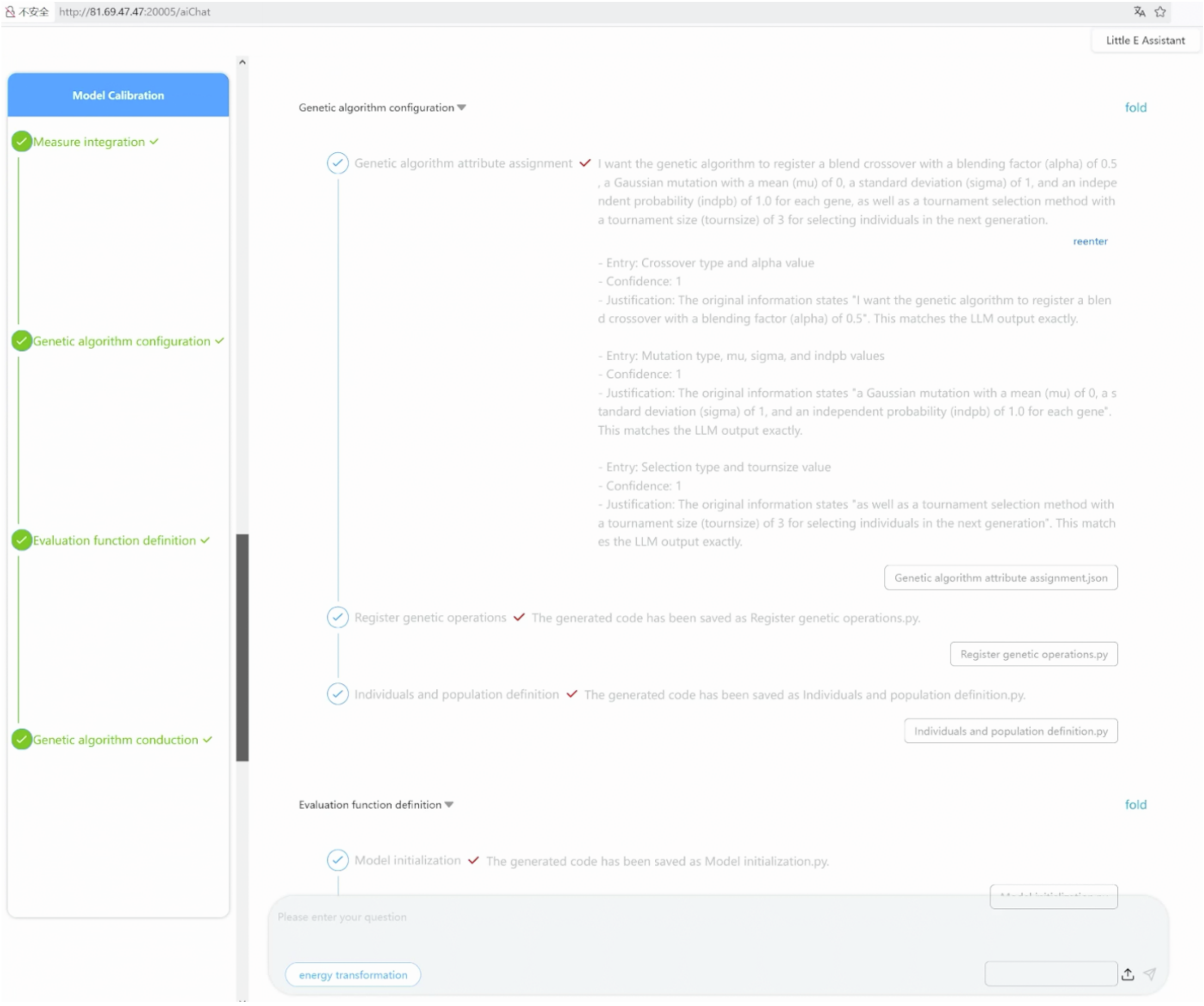

Figure S11 Interactive page of Genetic algorithm configuration. [Related to Results]

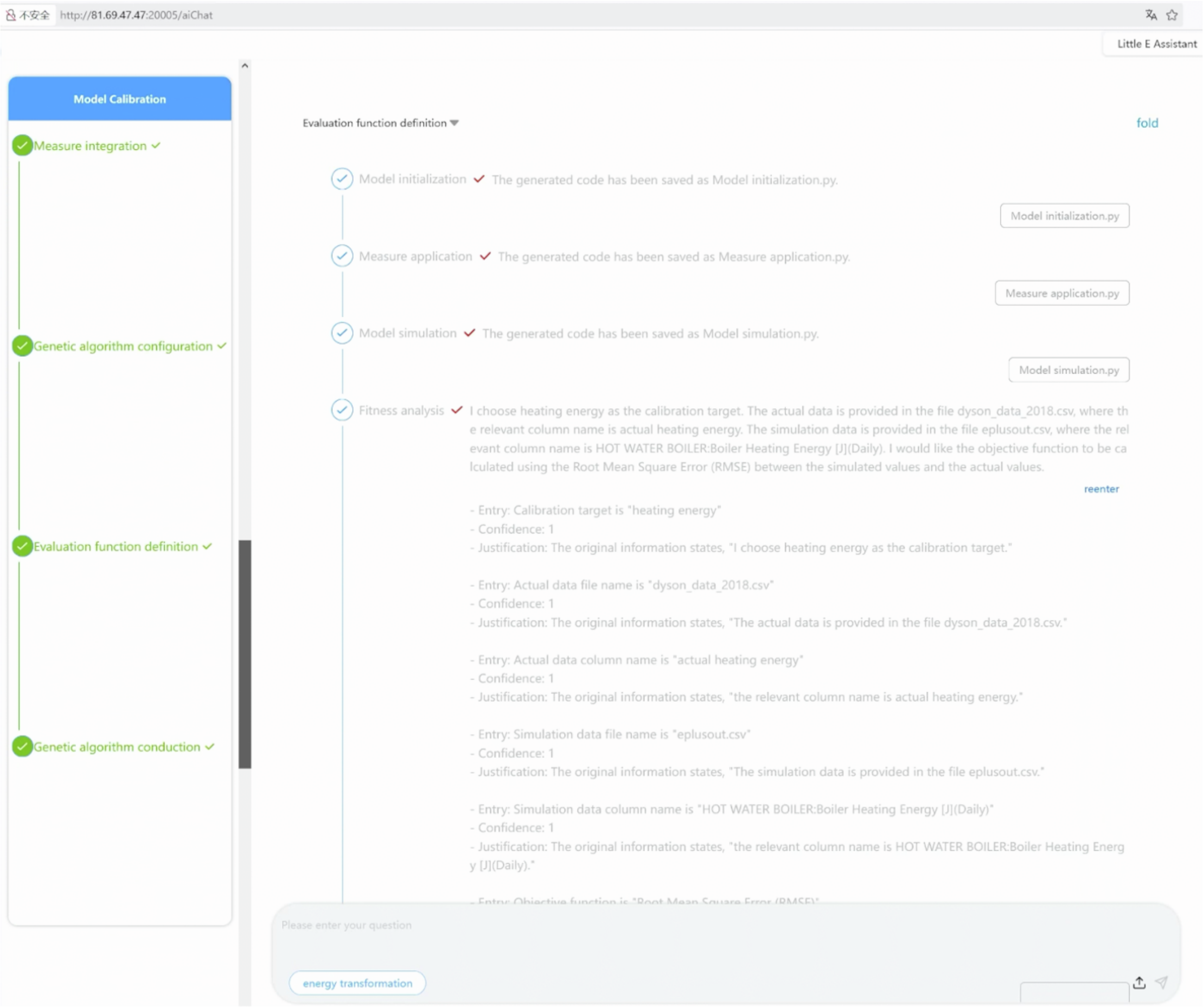

Figure S12 Interactive page of Evaluation function definition. [Related to Results]

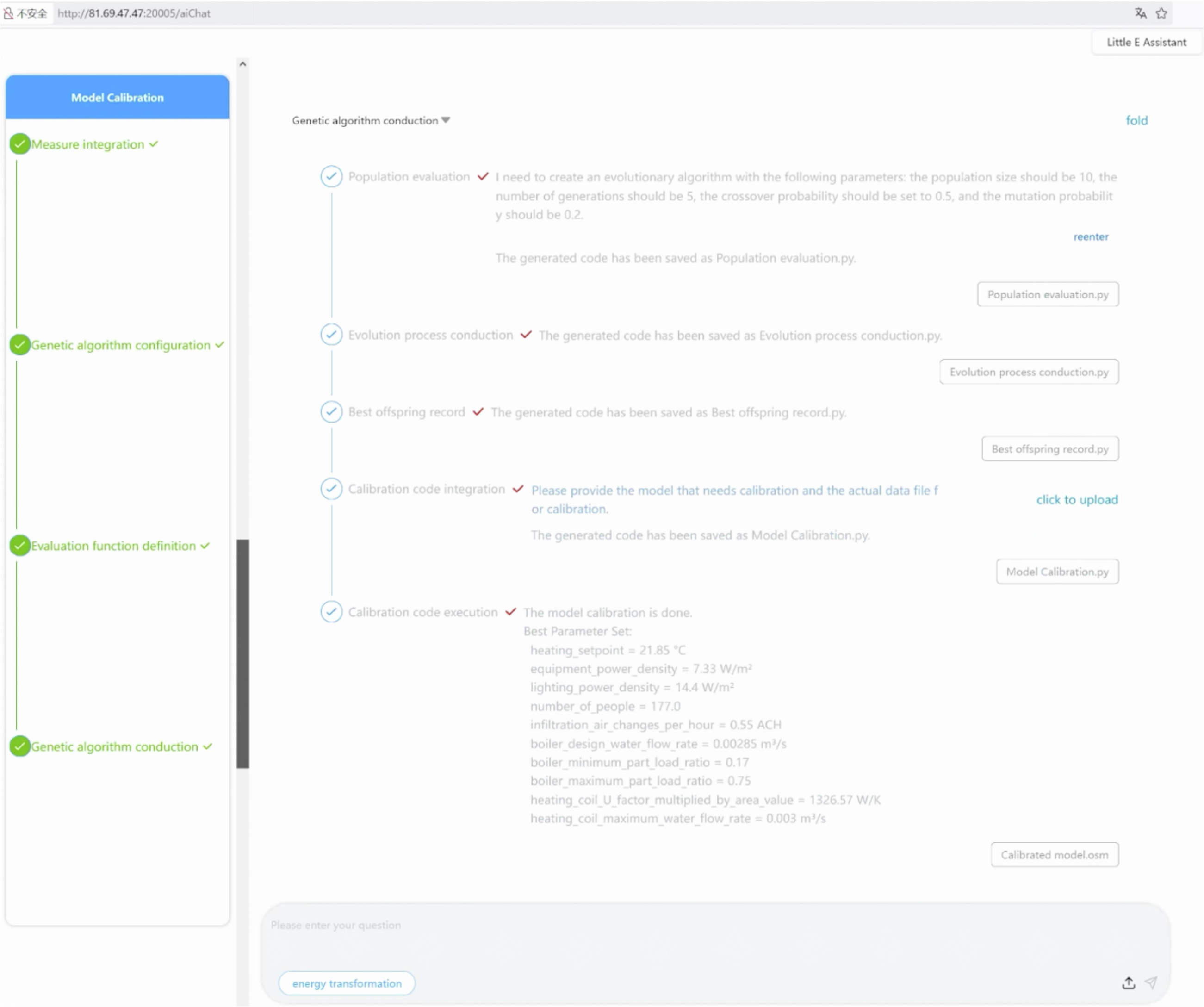

Figure S13 Interactive page of Genetic algorithm conduction. [Related to Results]

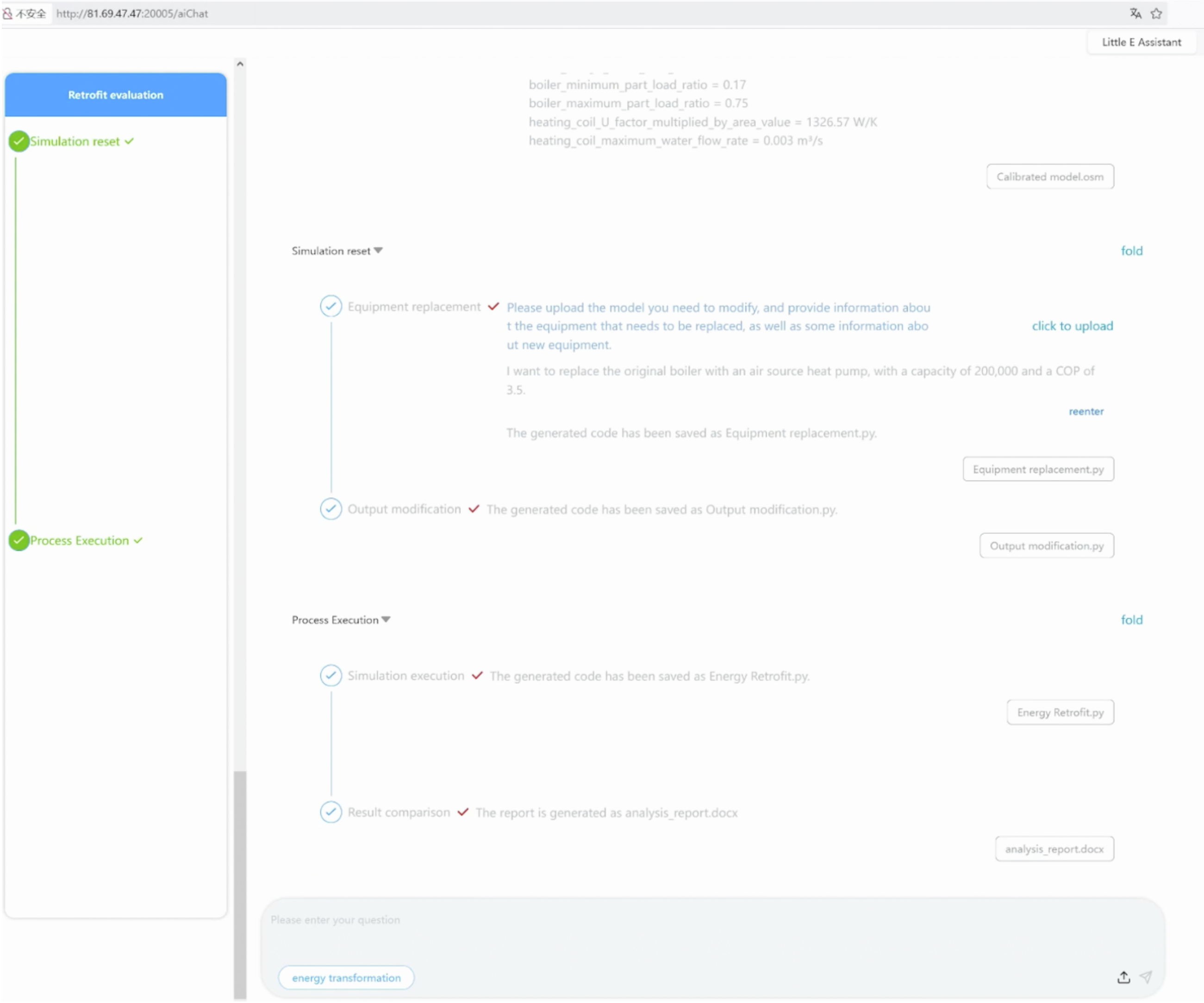

Figure S14 Interactive page of Energy Retrofits. [Related to Results]

Prompt 1

The building's heating system is a centralized hot water-based network that includes a Hot Water Loop for circulating hot water, Hot Water Nodes for flow distribution, and Hot Water Pumps to ensure consistent circulation. A Boiler serves as the primary heat source, …

Answers under Prompt 1

HVAC system composition extraction

```
{
  "HVAC System": {
    "Heating System": "Centralized hot water-based network",
    "Components": {
      "Hot Water Loop": "Circulates hot water",
      "Hot Water Nodes": "Flow distribution",
      "Hot Water Pumps": "Ensure consistent circulation",
      "Boiler": "Primary heat source"}
    }
}
```

Parameter range assignment

```
# Hot Water Loop
hot_water_plant = openstudio.model.PlantLoop(model)
hot_water_plant.setName("Hot Water Loop")
for thermal_zone in model.getThermalZones():
    sizing_zone = openstudio.model.SizingZone(model,
thermal_zone)
    sizing_zone.setZoneHeatingDesignSupplyAirTemperature(24)
    sizing_zone.setHeatingDesignAirFlowMethod("None")
sizing_plant = hot_water_plant.sizingPlant()
sizing_plant.setLoopType("Heating")
sizing_plant.setDesignLoopExitTemperature(75)
sizing_plant.setLoopDesignTemperatureDifference(10)

# Hot Water Node
hot_water_supply_outlet_node =
hot_water_plant.supplyOutletNode()
hot_water_supply_inlet_node = hot_water_plant.supplyInletNode()

# Hot Water Pump
hot_water_pump = openstudio.model.PumpVariableSpeed(model)
hot_water_pump.setName("Hot Water Loop Pump")
hot_water_pump.addToNode(hot_water_supply_inlet_node)

# Boiler
boiler = openstudio.model.BoilerHotWater(model)
boiler.setName("Hot Water Boiler")
boiler.setBoilerFlowMode("LeavingSetpointModulated")
boiler.setNominalThermalEfficiency(1)
boiler.setWaterOutletUpperTemperatureLimit(45)
boiler.setSizingFactor(1.25)
hot_water_plant.addSupplyBranchForComponent(boiler)
boiler.setNominalCapacity(240000)
boiler.setDesignWaterFlowRate(0.005)
boiler.setMinimumPartLoadRatio(0.15)
boiler.setMaximumPartLoadRatio(0.6)
```

Prompt 2

STRUCTURAL COMPONENTS:
Primary heat transfer medium: Pressurized hot water
Circulation backbone: Hot Water Loop infrastructure
Flow control elements: Hot Water Nodes (zone distributors)
Hydraulic drivers: Hot Water Pumps (variable speed)
Thermal generation: High-efficiency Boiler unit…

Answers under Prompt 2

HVAC system composition extraction

```
{
  "HVAC System Composition": {
    "Primary heat transfer medium": "Pressurized hot water",
    "Circulation backbone": "Hot Water Loop infrastructure",
    "Flow control elements": "Hot Water Nodes (zone
distributors)",
    "Hydraulic drivers": "Hot Water Pumps (variable speed)",
    "Thermal generation": "High-efficiency Boiler unit"}
}
```

Parameter range assignment

```
# Hot Water Loop
hot_water_plant = openstudio.model.PlantLoop(model)
hot_water_plant.setName("Hot Water Loop")
for thermal_zone in model.getThermalZones():
    sizing_zone = openstudio.model.SizingZone(model,
thermal_zone)
    sizing_zone.setZoneHeatingDesignSupplyAirTemperature(24)
    sizing_zone.setHeatingDesignAirFlowMethod("None")
sizing_plant = hot_water_plant.sizingPlant()
sizing_plant.setLoopType("Heating")
sizing_plant.setDesignLoopExitTemperature(75)
sizing_plant.setLoopDesignTemperatureDifference(10)

# Hot Water Node
hot_water_supply_outlet_node =
hot_water_plant.supplyOutletNode()
hot_water_supply_inlet_node = hot_water_plant.supplyInletNode()

# Hot Water Pump
hot_water_pump = openstudio.model.PumpVariableSpeed(model)
hot_water_pump.setName("Hot Water Loop Pump")
hot_water_pump.addToNode(hot_water_supply_inlet_node)

# Boiler
boiler = openstudio.model.BoilerHotWater(model)
boiler.setName("Hot Water Boiler")
boiler.setBoilerFlowMode("LeavingSetpointModulated")
boiler.setNominalThermalEfficiency(1)
boiler.setWaterOutletUpperTemperatureLimit(45)
boiler.setSizingFactor(1.25)
hot_water_plant.addSupplyBranchForComponent(boiler)
boiler.setNominalCapacity(240000)
boiler.setDesignWaterFlowRate(0.005)
boiler.setMinimumPartLoadRatio(0.15)
boiler.setMaximumPartLoadRatio(0.6)
```

Figure S15 Comparison of HVAC system information extraction and code generation results under different prompt styles. [Related to Discussion]

Table S1- S2

Table S1 The impact of different types of noise in the prompts on the Information Retriever. [Related to Discussion]

Noise Type	Example Prompt	Information Retriever Output
Different Tone Styles	STRUCTURAL COMPONENTS:<br>Primary heat transfer medium: Pressurized hot water<br>Circulation backbone: Hot Water Loop infrastructure<br>Flow control elements: Hot Water Nodes (zone distributors)<br>Hydraulic drivers: Hot Water Pumps (variable speed)<br>Thermal generation: High-efficiency Boiler unit<br>Control system: Setpoint Manager with PID logic<br>Pressure regulation: Dual bypass system (Supply/Demand Bypass Pipes)<br>Terminal units: Aluminum-finned Radiators	{<br>"HVAC System Composition": {<br>"Primary heat transfer medium": "Pressurized hot water",<br>"Circulation backbone": "Hot Water Loop infrastructure",<br>"Flow control elements": "Hot Water Nodes (zone distributors)",<br>"Hydraulic drivers": "Hot Water Pumps (variable speed)",<br>"Thermal generation": "High-efficiency Boiler unit",<br>"Control system": "Setpoint Manager with PID logic",<br>"Pressure regulation": "Dual bypass system (Supply/Demand Bypass Pipes)",<br>"Terminal units": "Aluminum-finned Radiators"<br>}<br>}
Spelling Mistakes	The buidling's heating sytem is a centrilized hot wateer-based netwrok with a Hot Watre Loop, Hot Watre Ndoes, and Hot Water Pumpps for circultion. A Boiller acts as the primery heat sorce, while the Setpiont Manger controls temprature. The sysstem includes Suplly Bypas Pipes and Demmand Bypas Pipes. Radiattors provide heat to indoor sapces.	{<br>"heating_system": {<br>"type": "centralized hot water-based network",<br>"components": [<br>{<br>"name": "Hot Water Loop",<br>"function": "Distributes hot water throughout the system"<br>},<br>{<br>"name": "Hot Water Nodes",<br>"function": "Connects to the system for distributing heat"<br>},<br>{<br>"name": "Hot Water Pumps",<br>"function": "Circulates hot water within the system"<br>},<br>{<br>"name": "Boiler",

Noise Type	Example Prompt	Information Retriever Output
		"function": "Primary heat source"<br>},<br>{<br>"name": "Setpoint Manager",<br>"function": "Controls temperature"<br>},<br>{<br>"name": "Supply Bypass Pipes",<br>"function": "Redirects water to maintain flow under specific conditions"<br>},<br>{<br>"name": "Demand Bypass Pipes",<br>"function": "Redirects water to balance demand"<br>},<br>{<br>"name": "Radiators",<br>"function": "Provides heat to indoor spaces"<br>}<br>]<br>}<br>}
Extra Words	Simulate a building's sophisticated heating system, which is designed as a centralized, hot water-based network. This intricate system features a Hot Water Loop, which efficiently circulates the hot water throughout the building, ensuring warmth in every corner. The Hot Water Nodes, strategically placed across the structure, serve as the crucial flow distribution elements, carefully directing the flow of the water where it is most needed. To maintain smooth and consistent circulation, the Hot Water Pumps tirelessly ensure the water moves with precision and reliability. At the heart of the system, a high-performance Boiler acts as the primary heat source, providing the necessary thermal energy to keep the	{<br>"HVAC_system": {<br>"Hot_Water_Loop": {<br>"description": "A centralized, hot water-based network that circulates hot water throughout the building."<br>},<br>"Hot_Water_Nodes": {<br>"description": "Strategically placed flow distribution elements that direct the flow of hot water where needed."<br>},<br>"Hot_Water_Pumps": {<br>"description": "Pumps that ensure precise and reliable movement of hot water throughout the system."<br>},<br>"Boiler": {<br>"description": "A high-performance heat source that provides thermal energy to the system."<br>},<br>"Setpoint_Manager": {

Noise Type	Example Prompt	Information Retriever Output
	building comfortable. The Setpoint Manager, an intelligent controller, expertly optimizes the temperature, adjusting dynamically for maximum comfort. To enhance system stability and support the flow of water, the system is equipped with both Supply Bypass Pipes and Demand Bypass Pipes, each playing a pivotal role in maintaining balance and ensuring the proper distribution of hot water. Finally, the Radiators, strategically placed throughout the building, transfer heat into the indoor spaces, ensuring a comfortable and energy-efficient environment. This system works in harmony, providing a seamless and efficient heating solution for the building, ensuring both comfort and operational effectiveness.	"description": "An intelligent controller that dynamically adjusts the temperature for maximum comfort."<br>},<br>"Supply_Bypass_Pipes": {<br>"description": "Pipes that maintain system stability and support water flow."<br>},<br>"Demand_Bypass_Pipes": {<br>"description": "Pipes that help maintain balance and ensure proper distribution of hot water."<br>},<br>"Radiators": {<br>"description": "Devices strategically placed to transfer heat into the indoor spaces for comfort."<br>}<br>}<br>}

Table S2 The impact of different types of noise in the prompts on the Information Retriever. [Related to Discussion]

**Noise Type**	**Example Prompt**	**Information Retriever Output**
Different Tone Styles	We propose to designate the following parameters as key identification variables, within their respective operational ranges:<br>Temperature Settings for Heating Systems: The temperature is maintained between 18°C and 24°C.<br>Power Density of Equipment: The power density ranges from 3 W/m^2 to 22 W/m^2.<br>Power Density of Lighting Systems: The lighting power density is set between 1.5 W/m^2 and 15 W/m^2.<br>Occupancy Levels: Occupancy is adjusted from 30 to 200 people, based on spatial dimensions and office configurations.<br>Hourly Air Change Rate for Infiltration (ACH): The ACH is calibrated between 0.5 and 1.5, according to environmental conditions.<br>Boiler Design Water Flow Rate: The water flow rate for the boiler ranges from 0.0001 m^3/s to 0.005 m^3/s.<br>Boiler Minimum and Maximum Operational Capacity Ratios: The minimum load ratio is between 0.1 and 0.4, while the maximum load ratio spans from 0.6 to 0.9.<br>Heat Transfer Coefficient-Area Product for Heating Coils: This value is set between 500.0 W/K and 2000.0 W/K.<br>Peak Water Flow Capacity for Heating Coils: The maximum water flow rate for the heating coils is between 0.001 m^3/s and 0.006 m^3/s.	{<br>"calibration_parameters": [<br>{<br>"parameter": "temperature settings for heating systems",<br>"range": {"min": 18, "max": 24, "unit": "°C"}<br>},<br>{<br>"parameter": "power density of equipment",<br>"range": {"min": 3, "max": 22, "unit": "W/m^2"}<br>},<br>{<br>"parameter": "power density of lighting systems",<br>"range": {"min": 1.5, "max": 15, "unit": "W/m^2"}<br>},<br>{<br>"parameter": "occupancy levels",<br>"range": {"min": 30, "max": 200, "unit": "persons"}<br>},<br>{<br>"parameter": "hourly air change rate for infiltration",<br>"range": {"min": 0.5, "max": 1.5, "unit": "ACH"}<br>},<br>{<br>"parameter": "design water flow rate for boilers",<br>"range": {"min": 0.0001, "max": 0.005, "unit": "m^3/s"}<br>},<br>{<br>"parameter": "minimum operational capacity ratio for boilers",<br>"range": {"min": 0.1, "max": 0.4, "unit": ""}

Noise Type	Example Prompt	Information Retriever Output

		},
		 {
		 "parameter": "maximum operational capacity ratio for boilers",
		 "range": {"min": 0.6, "max": 0.9, "unit": ""}
		 },
		 {
		 "parameter": "heat transfer coefficient-area product for heating coils",
		 "range": {"min": 500.0, "max": 2000.0, "unit": "W/K"}
		 },
		 {
		 "parameter": "peak water flow capacity for heating coils",
		 "range": {"min": 0.001, "max": 0.006, "unit": "m³/s"}
		 }
		]
		 }

Spelling Mistakes	We intend to select the following parametres as key identificashun variables, each within their spesified rangers: the heating setpoit will range from 18°C to 24°C; equiptment power densoty will vary between 3 W/m² and 22 W/m²; lighting power densoty is set from 1.5 W/m² to 15 W/m². The number of peopple accommadated will range from 30 to 200, ajusted based on bilding area and offcie scenrios. Infiltration air chages per hour (ACH) wil be set between 0.5 and 1.5, tailored to actual conitions. Additionally, the boilder design water flow rate will range from 0.0001 m³/s to 0.005 m³/s, the boiler minimim part load ratio wil be between 0.1 and 0.4, and the boiler maximum part load ratio wil vary from 0.6 to 0.9. The heating coil U-factur mulitplied by area value will be set between 500.0 W/K and 2000.0 W/K, and the heating coil maximuum water flow	{
		 "heating_setpoint_range": {"min": 18, "max": 24},
		 "equipment_power_density_range": {"min": 3, "max": 22},
		 "lighting_power_density_range": {"min": 1.5, "max": 15},
		 "people_accommodation_range": {"min": 30, "max": 200},
		 "infiltration_air_changes_per_hour_range": {"min": 0.5, "max": 1.5},
		 "boiler_design_water_flow_rate_range": {"min": 0.0001, "max": 0.005},
		 "boiler_minimum_part_load_ratio_range": {"min": 0.1, "max": 0.4},
		 "boiler_maximum_part_load_ratio_range": {"min": 0.6, "max": 0.9},
		 "heating_coil_u_factor_area_range": {"min": 500.0, "max": 2000.0},
		 "heating_coil_maximum_water_flow_rate_range": {"min": 0.001, "max": 0.006}
		 }

Noise Type	Example Prompt	Information Retriever Output
	rate wil range from 0.001 m^3/s to 0.006 m^3/s.	
Extra Words	We intend to carefully select the following key parameters as essential identification variables, each to be considered within their specifically defined ranges: The heating setpoint, which is of great importance, will range between 18°C and 24°C; equipment power density, which is crucial to our design, will vary between 3 W/m^2 and 22 W/m^2; lighting power density, set for optimal brightness, will be between 1.5 W/m^2 and 15 W/m^2. The number of people accommodated in the building will vary, ranging from 30 to 200, depending on the size of the building and the specific office scenarios present. Infiltration air changes per hour (ACH), which plays a vital role in air quality, will be set between 0.5 and 1.5, adjusted according to the actual environmental conditions. Additionally, the boiler design water flow rate, a critical factor, will range from 0.0001 m^3/s to 0.005 m^3/s, while the boiler minimum part load ratio, important for efficiency, will be between 0.1 and 0.4. The maximum part load ratio of the boiler will vary between 0.6 and 0.9, to ensure flexibility and performance. The heating coil U-factor multiplied by the area value, essential for heat transfer, will be set between 500.0 W/K and 2000.0 W/K, while the maximum water flow rate through the heating coil will range from 0.001 m^3/s to 0.006 m^3/s to meet the building's thermal requirements effectively.	{<br>"heating_setpoint": {"min": 18, "max": 24},<br>"equipment_power_density": {"min": 3, "max": 22},<br>"lighting_power_density": {"min": 1.5, "max": 15},<br>"number_of_people": {"min": 30, "max": 200},<br>"infiltration_ach": {"min": 0.5, "max": 1.5},<br>"boiler_design_water_flow_rate": {"min": 0.0001, "max": 0.005},<br>"boiler_min_part_load_ratio": {"min": 0.1, "max": 0.4},<br>"boiler_max_part_load_ratio": {"min": 0.6, "max": 0.9},<br>"heating_coil_u_factor_area_value": {"min": 500.0, "max": 2000.0},<br>"heating_coil_max_water_flow_rate": {"min": 0.001, "max": 0.006}<br>}

Data S1-Construction of multi-agent system

Information Retriever

The Information Retriever is designed to extract relevant data systematically and efficiently from specified data sources according to predefined instructions. This agent ensures comprehensive, precise data collection, crucial for building accurate digital twin models. It leverages specified tools from a provided knowledge base, maintaining clarity, consistency, and structural coherence. The retriever's outputs clearly define input sources and output formats, facilitating seamless subsequent computational processes.

Programmer

The Programmer is a specialized AI agent responsible for translating explicit instructions into precise, executable Python code. This agent strictly utilizes predefined tool functions detailed in a knowledge base, ensuring code integrity and adherence to task specifications. Emphasizing conciseness, efficiency, and accuracy, the Programmer produces well-organized, self-contained Python scripts without extraneous explanations or comments, explicitly defining all necessary inputs and outputs to ensure clarity and operational effectiveness.

Result Analyzer

The Result Analyzer generates analytical Python code to systematically evaluate and compare simulation outcomes between original and updated digital twin models. Operating exclusively with predefined tool functions, the agent extracts critical performance metrics, conducts thorough comparative analyses, and summarizes essential findings. The analyzer's final output is structured Python code capable of automating the evaluation process and producing comprehensive summary reports in Word format, facilitating clear and actionable insights.

Reviewer

The Reviewer evaluates the outputs generated by the LLM by comparing them with the original source materials or user inputs. This process involves a critical analysis of each claim, checking whether it is accurately derived from the source and whether it maintains consistency with other relevant information. The Reviewer assigns a confidence score to each output, ranging from 0 to 1, reflecting the degree of alignment with the source material. A score of 1 indicates complete accuracy and fidelity, while a score of 0 suggests a fundamental error or misrepresentation.

The confidence score is influenced by factors such as how well the information matches the original source, whether it is supported by reliable evidence, and the consistency of the information with other verified claims. Additionally, the Reviewer considers any potential discrepancies or errors, adjusting the score accordingly. In cases where the LLM output contains inaccuracies, hallucinations, or unsupported claims, the confidence score is lowered, with clear justifications provided for the adjustments. The Reviewer's ultimate aim is to ensure that the LLM-generated content is reliable, factually correct, and free from misleading or incorrect information.

Data S2-Geometry modeling

Users can interact with the LLM-agent integrated into the geometry modeling module to intuitively solve tasks in this section. The interface for geometric modeling, where users can directly engage with the agent, is displayed in Figure S5. In this section, the agent processes specific design data, such as CAD drawings and construction parameters, to generate key scripts like geometric outlines and envelope component properties.

Geometric information extraction

In this step, the geometric outline of a single building floor is extracted directly from the provided CAD drawing file. The agent processes the .dxf file and outputs a precise list of coordinate points representing the external perimeter of the specified floor. Internal details such as furniture are explicitly excluded from the result, ensuring that only the outer boundary is captured. The extracted contour coordinates can then be directly used for further modeling or analysis tasks.

The dialogue with information retriever agent for extracting the floor geometric information from a CAD drawing:

Agent: Information Retriever (Consensus, gpt-4)

Files:

CAD drawing.dxf

System Prompt:

Extract the coordinate points of the external boundary (outline) of a single floor from the uploaded CAD drawing (.dxf file). The extracted outline should include only the outer perimeter of the floor, excluding all internal features such as rooms, walls, or furniture. Return the coordinates as an ordered list of (x, y) tuples.

Example Response:

[(x1, y1), (x2, y2), …]

Answer:

[(4.003333, 44.385), (20.588333, 44.385), (20.588333, 4.67), (4.003333, 4.67), (4.003333, 11.43), (-5.070669, 11.43), (-5.070669, 23.521099), (-6.759763, 23.521099), (-6.759763, 26.03906), (-3.789287, 26.03906), (-3.789287, 29.034179), (4.003333, 29.034179), (4.003333, 44.385)]

Envelope data extraction

This step involves extracting detailed thermal and optical performance parameters of the building envelope components—including walls, windows, roofs, and floors—from provided construction design documents (Construction information.pdf). The agent tasked with this role is the Information Retriever. Given user-provided specifications such as solar, visible, and thermal absorptance values for wall, roof, and floor components, the agent outputs structured data in a standardized JSON format. The final output contains precise values for thermal resistance, solar heat gain coefficient, visible transmittance, and absorptance properties, ensuring accurate and consistent inputs for subsequent modeling tasks.

The dialogue with Retriever agent for extracting envelope data based on construction design documents:

Agent: Information Retriever (Consensus, gpt-4)

Files:

Construction information.pdf

System Prompt:

Extract information about building envelope components from the design documents, including walls, roofs, floors, and windows. Please include relevant data such as thermal resistance, solar absorption rate, visible light absorption rate, heat absorption rate, and other relevant thermodynamic data.

Example Response:

JSON format

Answer (extracted_information.json):


```
{
    "Wall": {
        "Thermal Resistance": "0.16 W/m²·K",
        "Solar Absorptance": "0.6",
        "Visible Absorptance": "0.6",
        "Thermal Absorptance": "0.9"
    },
    "Window": {
        "Thermal Resistance": "1.8 W/m²·K",
        "Solar Heat Gain Coefficient": "0.4",
        "Visible Transmittance": "66 %"
    },
    "Roof": {
        "Thermal Resistance": "0.12 W/m²·K",
        "Solar Absorptance": "0.7",
        "Visible Absorptance": "0.7",
        "Thermal Absorptance": "0.9"
    },
    "Floor": {
        "Thermal Resistance": "0.22 W/m²·K",
        "Solar Absorptance": "0.83",
        "Visible Absorptance": "0.83",
        "Thermal Absorptance": "0.9"
    }
}
```


In this step, the document referenced is "Construction information.pdf", which provides a detailed breakdown of the building's performance characteristics based on Part L compliance. It includes data on various elements such as thermal performance, ventilation, lighting, and heating systems. The document specifies values for U-values, solar heat gain coefficients, light transmittance, air permeability, and mechanical ventilation efficiency, both at the design stage and as constructed values.

The dialogue with Reviewer agent for evaluating whether the information extracted is accurate:

Agent: Reviewer (Consensus, gpt-4)

File:

Construction information.pdf

extracted_information.json

Answer:

- Entry: Wall U-values (area weighted average) is "0.16 W/m2.K"
- Confidence: 1
- Justification: The original information states "Walls (All vertical solid elements, except where indicated below) U-values (area weighted average)" is "0.16 W/m2.K".
- Entry: Wall Solar Absorptance is "0.6"
- Confidence: 1
- Justification: The original information states "Wall: Solar Absorptance = 0.6".

- Entry: Wall Visible Absorptance is "0.6"
- Confidence: 1
- Justification: The original information states "Wall: Visible Absorptance = 0.6".
- Entry: Wall Thermal Absorptance is "0.9"
- Confidence: 1
- Justification: The original information states "Wall: Thermal Absorptance = 0.9".
- Entry: Roof U-values (area weighted average) is "0.12 W/m2.K"
- Confidence: 1
- Justification: The original information states "Roof U-values (area weighted average)" is "0.12 W/m2.K".
- Entry: Roof Solar Absorptance is "0.7"
- Confidence: 1
- Justification: The original information states "Roof: Solar Absorptance = 0.7".
- Entry: Roof Visible Absorptance is "0.7"
- Confidence: 1
- Justification: The original information states "Roof: Visible Absorptance = 0.7".
- Entry: Roof Thermal Absorptance is "0.9"
- Confidence: 1
- Justification: The original information states "Roof: Thermal Absorptance = 0.9".
- Entry: Floor U-values (area weighted average) is "0.22 W/m2.K"
- Confidence: 1
- Justification: The original information states "Floor U-values (area weighted average)" is "0.22 W/m2.K".
- Entry: Floor Solar Absorptance is "0.83"
- Confidence: 1
- Justification: The original information states "Floor: Solar Absorptance = 0.83".
- Entry: Floor Visible Absorptance is "0.83"
- Confidence: 1
- Justification: The original information states "Floor: Visible Absorptance = 0.83".
- Entry: Floor Thermal Absorptance is "0.9"
- Confidence: 1
- Justification: The original information states "Floor: Thermal Absorptance = 0.9".
- Entry: Windows Overall U-value (including frame) is "1.8 W/m2.K"
- Confidence: 1
- Justification: The original information states "Overall U-value (including frame)" is "1.8 W/m2.K".
- Entry: Windows Solar heat gain coefficient general (g-value EN 410) is "0.4"
- Confidence: 1
- Justification: The original information states "Solar heat gain coefficient general (g-value EN 410)" is "0.4".
- Entry: Windows Light transmittance general is "66 %"
- Confidence: 1

Justification: The original information states "Light transmittance general" is "66 %".

Geometry model creation

This step is dedicated to the automated creation of a 3D building geometry model based on the previously extracted geometric parameters. The designated agent for this task is the Programmer, who utilizes an algorithmic library to systematically process spatial data and generate the building's geometric structure, including floor heights, number of stories, and detailed spatial layout. The agent constructs OpenStudio model objects for each building component, assigns construction sets to envelope surfaces, and ensures that relevant material and structural properties are accurately integrated into the digital model. Input parameters, such as space type, window-to-wall ratio, sill height, and story height, are directly reflected in the code implementation. The resulting script provides a reproducible approach for assembling the building geometry, serving as the foundation for subsequent energy modeling and simulation.

The dialogue with Programmer agent for generating 3D geometric modeling code based on architectural parameters:

Agent: Programmer (Consensus, gpt-4)

User Prompt:

The building is an office with four floors, each having a floor height of 3.5 meters. The window-to-wall ratio is 0.6, and the sill height is 1.0 meter.

System Prompt:

Process the extracted geometric data using algorithm library to construct a 3D building geometric model, defining the spatial structure and layout of the building. Define construction sets for surfaces, integrating material and structural properties to represent the envelope accurately. Please strictly adhere to the format of the example code, including spaces.

Example Response:

```
space_type = CREATE SpaceType IN model
space_type.SET standards_space_type TO ExampleType
floor_height = ExampleHeight
num_floors = ExampleFloor

FOR each idx, ordered_points IN all_ordered_points:
  # Remove the duplicate last point
  os_points = CREATE Point3d FOR each point IN ordered_points except last, with z = 0

  # Create a Point3dVector
  point_vector = CREATE Point3dVector FROM os_points

  FOR each floor IN range(num_floors):
    z_offset = floor MULTIPLIED BY floor_height   # Height offset for each floor
    translated_points = TRANSLATE os_points BY z_offset ALONG z-axis
    point_vector = CREATE Point3dVector FROM translated_points

    # Create space
    space = CREATE Space FROM point_vector, floor_height, model
```

```
IF space IS successfully initialized:
    space = GET space object
    space.SET name TO "Building Space [idx+1] Floor [floor+1]"
    space.SET space_type TO space_type

    space.SET default_schedule_set TO default_schedule_set

    surfaces = GET surfaces FROM space
    FOR each surface IN surfaces:
        IF surface.TYPE IS "Wall":
            surface.SET construction TO wall_construction

            IF surface.OUTSIDE boundary_condition IS "Outdoors":
                wwr = ExampleWindowToWallRatio
                sill_height = ExampleSillHeight

                success = SET window_to_wall_ratio OF surface TO wwr, sill_height, enable_automatic
                IF success:
                    FOR each sub_surface IN surface.sub_surfaces:
                        IF sub_surface.TYPE IS "FixedWindow":
                            sub_surface.SET construction TO window_construction

        ELSE IF surface.TYPE IS "RoofCeiling":
            surface.SET construction TO roof_construction

        ELSE IF surface.TYPE IS "Floor":
            surface.SET construction TO floor_construction

    # Create and assign a thermal zone
    thermal_zone = CREATE ThermalZone IN model
    thermal_zone.SET name TO "Building Thermal Zone [idx+1] Floor [floor+1]"
    space.SET thermal_zone TO thermal_zone

    PRINT "Successfully created building space [idx+1] on floor [floor+1]."

    APPLY thermal_zone_schedules TO thermal_zone IN model

ELSE:
    PRINT "Failed to create building space [idx+1] on floor [floor+1]."
```

Answer:

```
space_type = openstudio.model.SpaceType(model)
```

```python
space_type.setStandardsSpaceType('Office')
floor_height = 3.5
num_floors = 4

for idx, ordered_points in enumerate(all_ordered_points):
    # Remove the duplicate last point
    os_points = [openstudio.Point3d(pt[0], pt[1], 0.0) for pt in ordered_points[:-1]]

    # Create a Point3dVector
    point_vector = openstudio.Point3dVector(os_points)

    for floor in range(num_floors):
        z_offset = floor * floor_height    # Height offset for each floor
        translated_points = [openstudio.Point3d(pt.x(), pt.y(), z_offset) for pt in os_points]
        point_vector = openstudio.Point3dVector(translated_points)

        # Create space
        space = openstudio.model.Space.fromFloorPrint(point_vector, floor_height, model)
        if space.is_initialized():
            space = space.get()
            space.setName(f"Building Space {idx+1} Floor {floor+1}")
            space.setSpaceType(space_type)

            space.setDefaultScheduleSet(default_schedule_set)

            surfaces = space.surfaces()
            for surface in surfaces:
                if surface.surfaceType() == "Wall":
                    surface.setConstruction(wall_construction)

                    if surface.outsideBoundaryCondition() == "Outdoors":
                        wwr = 0.6
                        sill_height = 1.0

                        success = surface.setWindowToWallRatio(wwr, sill_height, True)
                        if success:
                            for sub_surface in surface.subSurfaces():
                                if sub_surface.subSurfaceType() == "FixedWindow":
                                    sub_surface.setConstruction(window_construction)
                elif surface.surfaceType() == "RoofCeiling":
                    surface.setConstruction(roof_construction)
                elif surface.surfaceType() == "Floor":
                    surface.setConstruction(floor_construction)
```

```
            # Create and assign a thermal zone
            thermal_zone = openstudio.model.ThermalZone(model)
            thermal_zone.setName(f"Building Thermal Zone {idx+1} Floor {floor+1}")
            space.setThermalZone(thermal_zone)

            print(f"Successfully created building space {idx+1} on floor {floor+1}.")

            apply_thermal_zone_schedules(thermal_zone, model)

        else:
            print(f"Failed to create building space {idx+1} on floor {floor+1}.")
```

Material property assignment

This step is dedicated to programmatically assigning detailed material properties to each building envelope component—including walls, roofs, floors, and windows—using the extracted and structured data from previous steps. The agent responsible for this operation is the Programmer. Leveraging the JSON file of extracted parameters, the agent retrieves relevant thermal and optical properties (such as thermal resistance, absorptances, U-factor, SHGC, and visible transmittance) for each envelope component. This ensures that the model employs accurate, component-specific material definitions, supporting consistent simulation inputs for subsequent analyses.

The dialogue with the Programmer agent for assigning material property values in building envelope model functions:

Agent: Programmer (Consensus, gpt-4)

Files:

extracted_information.json

System Prompt:

Task Instruction:

Retrieve the relevant material properties for each envelope component (e.g., walls, roof, floor, and windows) from the provided JSON file. These properties should include parameters such as thermal resistance, conductivity, density, solar absorptance, visible absorptance, thermal absorptance, U-factor, solar heat gain coefficient (SHGC), and visible transmittance. Use the retrieved values to directly replace the corresponding placeholders in the envelope function with the actual numeric values, ensuring that the function uses specific numbers rather than variable names.

Example Response:

```
DEFINE function set_building_envelope_with_values(model):

    # For each envelope type (wall, roof, floor, window)
    FOR each envelope_component IN [wall, roof, floor]:
        CREATE a new material for envelope_component
        SET the name for the material
        SET the thermal resistance using value from JSON
        SET the solar absorptance using value from JSON
        SET the visible absorptance using value from JSON
        SET the thermal absorptance using value from JSON
```

        CREATE a construction for envelope_component
        SET the name for the construction
        INSERT the material into the construction

    # For window glazing
    CREATE a window material
    SET the name for the window material
    SET the U-factor using value from JSON
    SET the solar heat gain coefficient using value from JSON
    SET the visible transmittance using value from JSON

    CREATE a construction for window
    SET the name for the window construction
    INSERT the window material into the window construction

    RETURN all constructions for wall, window, roof, and floor

Answer:

```
def set_building_envelope_with_values(model):
    # Wall material and construction
    wall_material = openstudio.model.MasslessOpaqueMaterial(model)
    wall_material.setName("Wall Material")
    wall_material.setThermalResistance(0.16)
    wall_material.setSolarAbsorptance(0.6)
    wall_material.setVisibleAbsorptance(0.6)
    wall_material.setThermalAbsorptance(0.9)

    wall_construction = openstudio.model.Construction(model)
    wall_construction.setName("Wall Construction")
    wall_construction.insertLayer(0, wall_material)

    # Roof material and construction
    roof_material = openstudio.model.MasslessOpaqueMaterial(model)
    roof_material.setName("Roof Material")
    roof_material.setThermalResistance(0.12)
    roof_material.setSolarAbsorptance(0.7)
    roof_material.setVisibleAbsorptance(0.7)
    roof_material.setThermalAbsorptance(0.9)

    roof_construction = openstudio.model.Construction(model)
    roof_construction.setName("Roof Construction")
    roof_construction.insertLayer(0, roof_material)
```

```python
# Floor material and construction
floor_material = openstudio.model.MasslessOpaqueMaterial(model)
floor_material.setName("Floor Material")
floor_material.setThermalResistance(0.22)
floor_material.setSolarAbsorptance(0.83)
floor_material.setVisibleAbsorptance(0.83)
floor_material.setThermalAbsorptance(0.9)

floor_construction = openstudio.model.Construction(model)
floor_construction.setName("Floor Construction")
floor_construction.insertLayer(0, floor_material)

# Window material and construction
window_material = openstudio.model.SimpleGlazing(model)
window_material.setName("Window Glazing")
window_material.setUFactor(1.8)
window_material.setSolarHeatGainCoefficient(0.4)
window_material.setVisibleTransmittance(0.66)

window_construction = openstudio.model.Construction(model)
window_construction.setName("Window Construction")
window_construction.insertLayer(0, window_material)

return wall_construction, window_construction, roof_construction, floor_construction
```

Data S3-Parameter configuration

HVAC system

In this section, the agent processes input data, including system descriptions and component specifications, to extract, develop, and assign key HVAC system parameters. These outputs serve as the foundation for more detailed energy simulations and system analysis in the subsequent steps.

HVAC system composition extraction

This step focuses on extracting a detailed breakdown of the HVAC (Heating, Ventilation, and Air Conditioning) system configuration based on the user's requirements or building design specifications. The Information Retriever agent analyzes the provided input—such as descriptions of heating, circulation, and control elements—and generates a structured summary in JSON format. The output comprehensively lists the HVAC system's main subsystems and key components, ensuring clarity and consistency for downstream modeling or documentation processes.

The dialogue with the Information Retriever agent for extracting the HVAC system composition from user requirements:

Agent: Information Retriever (Consensus, gpt-4)

User Prompt:

The building's heating system is a centralized hot water-based network that includes a Hot Water Loop for circulating hot water, Hot Water Nodes for flow distribution, and Hot Water Pumps to ensure consistent circulation. A Boiler serves as the primary heat source, while the Setpoint Manager optimizes temperature control. The system also features Supply Bypass Pipes and Demand Bypass Pipes to maintain stability and ensure proper water flow. Finally, Radiators transfer heat to indoor spaces, providing a comfortable and efficient heating solution for the building.

System Prompt:

Extract HVAC system composition from user requirement. Please provide the results in a JSON format.

Example Response:

JSON format

Answer (extracted_HVAC_composition.json):


```
{
    "HVAC System": {
        "Heating System": "Centralized hot water-based network",
        "Components": {
            "Hot Water Loop": "Circulates hot water",
            "Hot Water Nodes": "Flow distribution",
            "Hot Water Pumps": "Ensure consistent circulation",
            "Boiler": "Primary heat source",
            "Setpoint Manager": "Optimizes temperature control",
            "Supply Bypass Pipes": "Maintain stability",
            "Demand Bypass Pipes": "Ensure proper water flow",
            "Radiators": "Transfer heat to indoor spaces"
        }
    }
}
```


The dialogue with Reviewer agent for evaluating whether the information extracted is accurate:

Agent: Reviewer (Consensus, gpt-4)

Files:

User Prompt of "HVAC system composition extraction"

extracted_HVAC_composition.json

Answer:

- Entry: "Heating System": "Centralized hot water-based network"
- Confidence: 1
- Justification: The original information states, "The building's heating system is a centralized hot water-based network."

- Entry: "Components": { "Hot Water Loop": "Circulates hot water", "Hot Water Nodes": "Flow distribution", "Hot Water Pumps": "Ensure consistent circulation", "Boiler": "Primary heat source", "Setpoint Manager": "Optimizes temperature control", "Supply Bypass Pipes": "Maintain stability", "Demand Bypass Pipes": "Ensure proper water flow", "Radiators": "Transfer heat to indoor spaces" }
- Confidence: 1

Justification: The original information provides a detailed description of each component and their functions, which matches the LLM output. For example, "Hot Water Loop for circulating hot water," "Hot Water Nodes for flow distribution," "Hot Water Pumps to ensure consistent circulation," "A Boiler serves as the primary heat source," "Setpoint Manager optimizes temperature control," "Supply Bypass Pipes and Demand Bypass Pipes to maintain stability and ensure proper water flow," and "Radiators transfer heat to indoor spaces."

System model development

This step entails the automated development of a complete system-level HVAC equipment model based on previously extracted component information. The designated agent, referred to as the Programmer, receives structured equipment data (extracted_HVAC_composition.json) alongside a model library containing code templates for various HVAC components (Model library.xlsx). The agent's task is to systematically match each HVAC component—such as boilers, pumps, and radiators—to its corresponding code snippet within the library and assemble these snippets into a unified Python script. The resulting output is a comprehensive, well-organized system equipment model that integrates all specified components.

The dialogue with Programmer agent for generating an integrated HVAC system model using extracted component data and model code library:

Agent: Programmer (Consensus, gpt-4)

Files:

extracted_HVAC_composition.json

Model library.xlsx

System Prompt:

Using the equipment data provided in the JSON file, identify the corresponding model code snippets for each HVAC component (e.g., boilers, pumps, radiators) from the model library. Combine these code snippets to generate a complete system equipment model in Python, ensuring that all components are integrated. The output should be a single, well-structured Python script representing the entire HVAC system.

Example Response:

Create hot water loop
CREATE a plant loop for hot water in the model
SET the name of the plant loop
Hot Water Node
GET the supply outlet node of the hot water plant
GET the supply inlet node of the hot water plant
Other component
...
Answer:

```
# Hot Water Loop
hot_water_plant = openstudio.model.PlantLoop(model)
hot_water_plant.setName("Hot Water Loop")
for thermal_zone in model.getThermalZones():
    sizing_zone = openstudio.model.SizingZone(model, thermal_zone)
    sizing_zone.setZoneHeatingDesignSupplyAirTemperature(actual numeric value)
    sizing_zone.setHeatingDesignAirFlowMethod("None")
sizing_plant = hot_water_plant.sizingPlant()
sizing_plant.setLoopType("Heating")
sizing_plant.setDesignLoopExitTemperature(actual numeric value)
sizing_plant.setLoopDesignTemperatureDifference(actual numeric value)

# Hot Water Node
hot_water_supply_outlet_node = hot_water_plant.supplyOutletNode()
hot_water_supply_inlet_node = hot_water_plant.supplyInletNode()

# Hot Water Pump
hot_water_pump = openstudio.model.PumpVariableSpeed(model)
hot_water_pump.setName("Hot Water Loop Pump")
hot_water_pump.addToNode(hot_water_supply_inlet_node)

# Boiler
boiler = openstudio.model.BoilerHotWater(model)
boiler.setName("Hot Water Boiler")
boiler.setBoilerFlowMode("LeavingSetpointModulated")
boiler.setNominalThermalEfficiency(actual numeric value)
boiler.setWaterOutletUpperTemperatureLimit(actual numeric value)
boiler.setSizingFactor(actual numeric value)
hot_water_plant.addSupplyBranchForComponent(boiler)
boiler.setNominalCapacity(actual numeric value)
boiler.setDesignWaterFlowRate(actual numeric value)
boiler.setMinimumPartLoadRatio(actual numeric value)
boiler.setMaximumPartLoadRatio(actual numeric value)

# Setpoint Manager
```

```
setpoint_manager = openstudio.model.SetpointManagerScheduled(model, hot_water_temp_schedule)
setpoint_manager.setControlVariable("Temperature")
setpoint_manager.addToNode(hot_water_supply_outlet_node)

# Supply Bypass Pipe
supply_bypass_pipe = openstudio.model.PipeAdiabatic(model)
hot_water_plant.addSupplyBranchForComponent(supply_bypass_pipe)

# Demand Bypass Pipe
demand_bypass_pipe = openstudio.model.PipeAdiabatic(model)
hot_water_plant.addDemandBranchForComponent(demand_bypass_pipe)

# Radiator
for thermal_zone in model.getThermalZones():
    heating_coil = openstudio.model.CoilHeatingWaterBaseboard(model)
    heating_coil.setName(f"Heating Coil for {thermal_zone.name().get()}")
    heating_coil.setMaximumWaterFlowRate(actual numeric value)
    heating_coil.setUFactorTimesAreaValue(actual numeric value)
    radiator = openstudio.model.ZoneHVACBaseboardConvectiveWater(model, radiator_schedule,
heating_coil)
    radiator.setName(f"Radiator in {thermal_zone.name().get()}")
    radiator.addToThermalZone(thermal_zone)
    hot_water_plant.addDemandBranchForComponent(heating_coil)
```

Component parameter extraction

This step focuses on extracting key system component parameters required for the thermal system model, based on both code variable definitions and the provided design documents. The agent responsible for this task, the Information Retriever, is instructed to map variable names from Code for System model development.py to actual values sourced from Construction information.pdf and Heating information.pdf, or from user-provided specifications. Given that the variable names in the code may not always directly correspond to those in the source documents, the agent interprets their meaning based on context to ensure accurate data mapping. All extracted values are compiled in a standardized JSON format, with variables that cannot be found set to null.

The dialogue with the Retriever agent for extracting component parameters based on project documentation:

Agent: Information Retriever (Consensus, gpt-4)

Files:

Code for System model development.py

Construction information.pdf

Heating information.pdf

System Prompt:

According to the variable names from the Code for System model development.py file where the values are marked as actual numeric value, extract the corresponding real values for these variables from the provided design documents or the user-provided information, depending on the context.

Note that the variable names in the Code for System model development.py file may not exactly match the names in the design documents or the user-provided information. Therefore, interpret the meaning of the variables based on their context and function to identify the correct values.

Compile all the retrieved values into a JSON format file. The JSON file must include all variable names from the Code for System model development.py file where the values are marked as actual numeric value. If a specific variable cannot be found in either the design documents or the user-provided information, just output null.

Example Response:

JSON format

Answer (extracted_parameter.json):


```
{
    "sizing_zone.setZoneHeatingDesignSupplyAirTemperature": 24,
    "sizing_plant.setDesignLoopExitTemperature": 75,
    "sizing_plant.setLoopDesignTemperatureDifference": 10,
    "boiler.setNominalThermalEfficiency": 1,
    "boiler.setWaterOutletUpperTemperatureLimit": 75,
    "boiler.setSizingFactor": 1.25,
    "boiler.setNominalCapacity": 240000,
    "boiler.setDesignWaterFlowRate": 0.005,
    "boiler.setMinimumPartLoadRatio": 0.15,
    "boiler.setMaximumPartLoadRatio": 0.6,
    "heating_coil.setMaximumWaterFlowRate": 0.001,
    "heating_coil.setUFactorTimesAreaValue": 500
```


In this step, the document referenced is "Heating information.pdf", which provides detailed specifications for the underfloor heating system. It includes essential data on heat requirements, maximum temperatures, flow rates, and system design parameters for various zones within the building. The document specifies total heat requirements, average heat requirements per square meter, as well as temperature settings for both primary flow and return water.

The dialogue with Reviewer agent for evaluating whether the information extracted is accurate:

Agent: Reviewer (Consensus, gpt-4)

Files:

User Requirement of " Component parameter extraction"

Construction information.pdf

Heating information.pdf

extracted_parameter.json

Answer:

- Entry: "sizing_zone.setZoneHeatingDesignSupplyAirTemperature": 24
- Confidence: 1
- Justification: The user-provided information includes "Zone Heating Design Supply Air Temperature: 24".

- Entry: "sizing_plant.setDesignLoopExitTemperature": 75

- Confidence: 1
- Justification: The original information includes "Primary Loop Flow Exit LTHW Temperature Limit" and "Primary heat source – Flow 0 C 75 Return 0 C 65".

- Entry: "sizing_plant.setLoopDesignTemperatureDifference": 10
- Confidence: 0
- Justification: The original information does not provide a value for "Design Temperature Difference between Flow and Return".

- Entry: "boiler.setNominalThermalEfficiency": 1
- Confidence: 0
- Justification: The original information does not provide a value for "Boiler Nominal Thermal Efficiency".

- Entry: "boiler.setWaterOutletUpperTemperatureLimit": 75
- Confidence: 1
- Justification: The original information includes "Primary Loop Flow Exit LTHW Temperature Limit" and "Primary heat source – Flow 0 C 75 Return 0 C 65".

- Entry: "boiler.setSizingFactor": 1.25
- Confidence: 1
- Justification: The user-provided information includes "Boiler Sizing Factor: 1.25".

- Entry: "boiler.setNominalCapacity": 240000
- Confidence: 1
- Justification: The user-provided information includes "Boiler Nominal Capacity: 240000".

- Entry: "boiler.setDesignWaterFlowRate": 0.005
- Confidence: 1
- Justification: The user-provided information includes "Boiler Design Water Flow Rate: 0.005".

- Entry: "boiler.setMinimumPartLoadRatio": 0.15
- Confidence: 1
- Justification: The user-provided information includes "Boiler Minimum Part Load Ratio: 0.15".

- Entry: "boiler.setMaximumPartLoadRatio": 0.6
- Confidence: 1
- Justification: The user-provided information includes "Boiler Maximum Part Load Ratio: 0.6".

- Entry: "heating_coil.setMaximumWaterFlowRate": 0.001
- Confidence: 1
- Justification: The user-provided information includes "Heating Coil Maximum Water Flow Rate: 0.001".

- Entry: "heating_coil.setUFactorTimesAreaValue": 500
- Confidence: 1

- Justification: The user-provided information includes "Heating Coil U-Factor Times Area Value: 500".

Component parameter assignment

This step involves programmatically assigning extracted component parameters to the system model code, ensuring that simulation inputs reflect accurate, case-specific values. The agent responsible for this task is the Programmer, who utilizes the parameter values stored in the extracted_parameter.json file. By systematically replacing placeholder values in the development.py script with the corresponding actual numeric values, the agent automates the parameter assignment process. This guarantees consistency, reproducibility, and efficiency in the model setup, as all key system components—such as hot water loops, pumps, boilers, setpoint managers, and radiators—are configured using the extracted data. The resulting code is thus tailored for subsequent energy simulation or system analysis.

The dialogue with Programmer agent for assigning extracted component parameters to the system model code:

Agent: Programmer (Consensus, gpt-4)

Files:

extracted_parameter.json

Code for System model development.py

System Prompt:

Using the extracted parameter values from json document, replace the placeholders marked as actual numeric value in the Code for System model development.py file with the corresponding real values.

Answer:

```
# Hot Water Loop
hot_water_plant = openstudio.model.PlantLoop(model)
hot_water_plant.setName("Hot Water Loop")
for thermal_zone in model.getThermalZones():
    sizing_zone = openstudio.model.SizingZone(model, thermal_zone)
    sizing_zone.setZoneHeatingDesignSupplyAirTemperature(24)
    sizing_zone.setHeatingDesignAirFlowMethod("None")
sizing_plant = hot_water_plant.sizingPlant()
sizing_plant.setLoopType("Heating")
sizing_plant.setDesignLoopExitTemperature(75)
sizing_plant.setLoopDesignTemperatureDifference(10)

# Hot Water Node
hot_water_supply_outlet_node = hot_water_plant.supplyOutletNode()
hot_water_supply_inlet_node = hot_water_plant.supplyInletNode()

# Hot Water Pump
hot_water_pump = openstudio.model.PumpVariableSpeed(model)
hot_water_pump.setName("Hot Water Loop Pump")
hot_water_pump.addToNode(hot_water_supply_inlet_node)

# Boiler
boiler = openstudio.model.BoilerHotWater(model)
```

```
boiler.setName("Hot Water Boiler")
boiler.setBoilerFlowMode("LeavingSetpointModulated")
boiler.setNominalThermalEfficiency(1)
boiler.setWaterOutletUpperTemperatureLimit(45)
boiler.setSizingFactor(1.25)
hot_water_plant.addSupplyBranchForComponent(boiler)
boiler.setNominalCapacity(240000)
boiler.setDesignWaterFlowRate(0.005)
boiler.setMinimumPartLoadRatio(0.15)
boiler.setMaximumPartLoadRatio(0.6)

# Setpoint Manager
setpoint_manager = openstudio.model.SetpointManagerScheduled(model, hot_water_temp_schedule)
setpoint_manager.setControlVariable("Temperature")
setpoint_manager.addToNode(hot_water_supply_outlet_node)

# Supply Bypass Pipe
supply_bypass_pipe = openstudio.model.PipeAdiabatic(model)
hot_water_plant.addSupplyBranchForComponent(supply_bypass_pipe)

# Demand Bypass Pipe
demand_bypass_pipe = openstudio.model.PipeAdiabatic(model)
hot_water_plant.addDemandBranchForComponent(demand_bypass_pipe)

# Radiator
for thermal_zone in model.getThermalZones():
    heating_coil = openstudio.model.CoilHeatingWaterBaseboard(model)
    heating_coil.setName(f"Heating Coil for {thermal_zone.name().get()}")
    heating_coil.setMaximumWaterFlowRate(0.001)
    heating_coil.setUFactorTimesAreaValue(500)
    radiator = openstudio.model.ZoneHVACBaseboardConvectiveWater(model, radiator_schedule,
heating_coil)
    radiator.setName(f"Radiator in {thermal_zone.name().get()}")
    radiator.addToThermalZone(thermal_zone)
    hot_water_plant.addDemandBranchForComponent(heating_coil)
```

Schedule

In this section, the agent processes operational data such as building schedules and internal heat source specifications to extract, create, and assign time-dependent operational profiles for various building systems. These outputs ensure the accurate representation of occupancy, lighting, heating, and ventilation patterns, providing a solid foundation for subsequent simulation and analysis tasks.

Schedule data extraction

This step focuses on extracting operational schedule information from the building's design documentation (Schedule.pdf). The Information Retriever agent processes the text to identify specific time ranges associated with various building areas and categorizes each schedule entry by its function—namely occupancy, lighting, equipment, ventilation, or heating. For each record, the agent outputs a structured JSON object containing the area name, the corresponding time range, and the assigned category. This structured data provides a comprehensive and standardized schedule for different zones within the building.

The dialogue with Retriever agent for extracting area-specific operational schedules from the design documentation:

Agent: Information Retriever (Consensus, gpt-4)

Files:

Schedule.pdf

System Prompt:

Extract time ranges (e.g., 08:00 - 18:00) along with the corresponding area names from the following building design text. Additionally, identify the category of each time range (must be one of the following: occupancy, lighting, equipment, ventilation, or heating). Provide the extracted information in JSON format, where each entry is an object with "area_name", "time_range", and "category" fields.

Example Response:

Provide the extracted information in JSON format, where each entry is an object with the following fields: "area_name", "time_range", "category"

Attention:

The category must be one of the following:
occupancy, lighting, equipment, ventilation, heating

Answer (extracted_schedule_information.json):


```json
[
    {
        "area_name": "Imtech Engineering Services Central Ltd",
        "time_range": "08:00 - 18:00",
        "category": "occupancy"
    },
    {
        "area_name": "University of Cambridge Department of Engineering",
        "time_range": "08:00 - 18:00",
        "category": "lighting"
    },
    {
        "area_name": "Reception",
        "time_range": "08:00 – 18:00",
        "category": "heating"
    },
    {
        "area_name": "Seminar Room G-03",
        "time_range": "08:00 – 18:00",
        "category": "occupancy"
```

```
    },
    {
        "area_name": "Seminar Room G-03",
        "time_range": "03:00 – 07:00",
        "category": "ventilation"
    },
    {
        "area_name": "Baker Teaching Room 2",
        "time_range": "08:00 – 18:00",
        "category": "occupancy"
    },
    {
        "area_name": "Baker Teaching Room 2",
        "time_range": "03:00 – 07:00",
        "category": "ventilation"
    },
    {
        "area_name": "Ground Floor ANVS & BMS Radiator Zones",
        "time_range": "08:00 – 18:00",
        "category": "heating"
    },
    {
        "area_name": "Mezzanine Floor ANVS & BMS Radiator Zones",
        "time_range": "08:00 – 18:00",
        "category": "heating"
    },
    {
        "area_name": "1st Floor ANVS & BMS Radiator Zones",
        "time_range": "08:00 – 18:00",
        "category": "heating"
    },
    {
        "area_name": "2nd Floor ANVS & BMS Radiator Zones",
        "time_range": "08:00 – 18:00",
        "category": "heating"
    },
    {
        "area_name": "3rd Floor ANVS & BMS Radiator Zones",
        "time_range": "08:00 – 18:00",
        "category": "heating"
    },
]
```

The dialogue with Reviewer agent for evaluating whether the information extracted is accurate:

Agent: Reviewer (Consensus, gpt-4)

Files:

Schedule.pdf

extracted_schedule_information.json

Answer:

- Entry: Area 'Reception Underfloor Heating' has a time range of '08:00 – 18:00' and falls under the category 'heating'.
- Confidence: 1
- Justification: The original information includes the line "['Reception Underfloor Heating', '08:00 – 18:00', 'Yes']".

- Entry: Area 'Seminar Room G-03' has a time range of '08:00 – 18:00' and falls under the category 'occupancy'.
- Confidence: 1
- Justification: The original information includes the line "['Seminar Room G-03 Occupied Times', '08:00 – 18:00', 'Yes']".

- Entry: Area 'Seminar Room G-03' has a time range of '03:00 – 07:00' and falls under the category 'ventilation'.
- Confidence: 1
- Justification: The original information includes the line "['Seminar Room G-03 Night Purge Allow Times', '03:00 – 07:00', 'No']".

- Entry: Area 'Baker Teaching Room 2' has a time range of '08:00 – 18:00' and falls under the category 'occupancy'.
- Confidence: 1
- Justification: The original information includes the line "['Baker Teaching Room 2 Occupied Times', '08:00 – 18:00', 'Yes']".

- Entry: Area 'Baker Teaching Room 2' has a time range of '03:00 – 07:00' and falls under the category 'ventilation'.
- Confidence: 1
- Justification: The original information includes the line "['Baker Teaching Room 2 Night Purge Allow Times', '03:00 – 07:00', 'No']".

- Entry: Area 'Ground Floor ANVS & BMS Radiator Zones' has a time range of '08:00 – 18:00' and falls under the category 'heating'.
- Confidence: 1
- Justification: The original information includes the line "['Ground Floor ANVS & BMS Radiator Zones', '08:00 – 18:00', 'Yes']".

- Entry: Area 'Mezzanine Floor ANVS & BMS Radiator Zones' has a time range of '08:00 – 18:00' and falls under the category 'heating'.
- Confidence: 1

- Justification: The original information includes the line "['Mezzanine Floor ANVS & BMS Radiator Zones', '08:00 – 18:00', 'Yes']".

- Entry: Area '1st Floor ANVS & BMS Radiator Zones' has a time range of '08:00 – 18:00' and falls under the category 'heating'.
- Confidence: 1
- Justification: The original information includes the line "['1st Floor ANVS & BMS Radiator Zones', '08:00 – 18:00', 'Yes']".

- Entry: Area '2nd Floor ANVS & BMS Radiator Zones' has a time range of '08:00 – 18:00' and falls under the category 'heating'.
- Confidence: 1
- Justification: The original information includes the line "['2nd Floor ANVS & BMS Radiator Zones', '08:00 – 18:00', 'Yes']".

- Entry: Area '3rd Floor ANVS & BMS Radiator Zones' has a time range of '08:00 – 18:00' and falls under the category 'heating'.
- Confidence: 1
- Justification: The original information includes the line "['3rd Floor ANVS & BMS Radiator Zones', '08:00 – 18:00', 'Yes']".

- Entry: Area 'Department of Engineering' has a time range of '08:00 – 18:00' and falls under the category 'lighting'.
- Confidence: 1

Justification: The original information includes the line "['Department of Engineering lighting time', '08:00 – 18:00', 'Yes']".

In this step, the document referenced is "Schedule.pdf", which outlines the operational time schedules for various equipment and systems within the building. It includes specific time zones for the operation of underfloor heating, seminar rooms, and ventilation systems, as well as the general operational hours for different zones. The document specifies how systems are activated based on occupancy, and details the start and stop times for different devices, such as underfloor heating and ventilation units, ensuring efficient energy usage and comfort control.

Schedule model creation

This step focuses on creating the schedule model that governs the operation of key building systems, including occupancy, lighting, equipment, heating, and ventilation. The agent responsible for this task is the Programmer, who receives retrieved schedule information in JSON format (merged_schedule_info.json) and generates Python code for OpenStudio to define the default daily schedules. The resulting schedules specify the time-dependent operation profiles for each system.

The dialogue with Programmer agent for generating Python code to create default schedule models using retrieved schedule information:

Agent: Programmer (Consensus, gpt-4)

Files:

merged_schedule_info.json

System Prompt:

Generate Python code that sets default schedules for occupancy, lighting, equipment, and heating using the retrieved schedule information in JSON. Use the provided pseudocode and retrieved information to generate executable Python code.

Example Response:

```
DEFINE a function to set default schedule set for the model
    CREATE a default schedule set object for the model
    FOR each schedule type in [occupancy, lighting, equipment, heating]:
        CREATE a schedule ruleset for the schedule type
        SET the name for the schedule ruleset
        GET the default day schedule from the ruleset
        FOR each time-value pair retrieved from the schedule information:
            ADD the time and corresponding value to the default day schedule
        ASSIGN the completed schedule ruleset to the appropriate field in the schedule set
    RETURN the completed schedule set
```

Answer:

```python
import openstudio
def set_default_schedule_set(model):
    schedule_set = openstudio.model.DefaultScheduleSet(model)

    occupancy_schedule = openstudio.model.ScheduleRuleset(model)
    occupancy_schedule.setName("Building Occupancy Schedule")
    occupancy_day_schedule = occupancy_schedule.defaultDaySchedule()
    occupancy_day_schedule.addValue(openstudio.Time(0, 8, 0), 0.0)
    occupancy_day_schedule.addValue(openstudio.Time(0, 18, 0), 1.0)
    occupancy_day_schedule.addValue(openstudio.Time(0, 24, 0), 0.0)
    schedule_set.setNumberofPeopleSchedule(occupancy_schedule)

    lighting_schedule = openstudio.model.ScheduleRuleset(model)
    lighting_schedule.setName("Building Lighting Schedule")
    lighting_day_schedule = lighting_schedule.defaultDaySchedule()
    lighting_day_schedule.addValue(openstudio.Time(0, 8, 0), 0.0)
    lighting_day_schedule.addValue(openstudio.Time(0, 18, 0), 1.0)
    lighting_day_schedule.addValue(openstudio.Time(0, 24, 0), 0.0)
    schedule_set.setLightingSchedule(lighting_schedule)

    heating_schedule = openstudio.model.ScheduleRuleset(model)
    heating_schedule.setName("Building Heating Schedule")
    heating_day_schedule = heating_schedule.defaultDaySchedule()
    heating_day_schedule.addValue(openstudio.Time(0, 8, 0), 0.0)
    heating_day_schedule.addValue(openstudio.Time(0, 18, 0), 1.0)
```

```
    heating_day_schedule.addValue(openstudio.Time(0, 24, 0), 0.0)
    schedule_set.setHeatingSchedule(heating_schedule)

    ventilation_schedule = openstudio.model.ScheduleRuleset(model)
    ventilation_schedule.setName("Building Ventilation Schedule")
    ventilation_day_schedule = ventilation_schedule.defaultDaySchedule()
    ventilation_day_schedule.addValue(openstudio.Time(0, 3, 0), 0.0)
    ventilation_day_schedule.addValue(openstudio.Time(0, 7, 0), 1.0)
    ventilation_day_schedule.addValue(openstudio.Time(0, 24, 0), 0.0)
    schedule_set.setVentilationSchedule(ventilation_schedule)

    return schedule_set
```

Internal heat source assignment

This step is dedicated to the detailed specification and allocation of internal heat sources within the building model, including occupants, lighting, equipment, and infiltration rates. These parameter values—such as number of people, activity levels, lighting and equipment power densities, and air change rates—are incorporated directly into the OpenStudio model through tailored scripts. The process ensures that internal heat gains and infiltration schedules accurately reflect the actual usage patterns and operational requirements of the modeled space.

Dialogue with Programmer agent for the assignment of internal heat source parameters based on user requirements:

Agent: Programmer (Consensus, gpt-4)

User Prompt:

The office area accommodates 73 people, with an activity level set to 80 watts per person from 08:00 to 17:00 and 20 watts per person from 17:00 to 20:00. The lighting power is configured at 6.56 watts per square meter, and the equipment power is set to 12.34 watts per square meter. The air change rate is established at 1.49 times per hour. Additionally, the infiltration schedule is programmed to perform infiltration from 03:00 to 20:00 daily, with no infiltration during other time periods.

System Prompt:

Use the provided pseudocode and user requirements to generate executable Python code.

Example Response:

```
# Define activity level schedule type limits
CREATE a schedule type limits object for activity level
SET the name, lower and upper limit values, numeric type, and unit type

# Define activity level schedule and its values based on user requirements
CREATE a ruleset schedule for activity level
SET the name and assign the schedule type limits
GET the default day schedule from the ruleset
FOR each time and activity value pair from user requirements:
    ADD a value to the day schedule at the specified time with the specified activity level
```

```
# For each space in the model, set up internal loads
FOR each space in the model:
    # People definition
    CREATE a people definition object for the space
    SET the name and number of people based on user requirements
    CREATE a people object from the people definition
    ASSIGN the space, number of people schedule, and activity level schedule to the people object

    # Lighting definition
    CREATE a lighting definition object for the space
    SET the name
    CREATE a lighting object from the lighting definition
    ASSIGN the space and lighting schedule to the lighting object
    SET the lighting power per area based on user requirements

    # Equipment definition
    CREATE an equipment definition object for the space
    SET the name
    CREATE an equipment object from the equipment definition
    ASSIGN the space and equipment schedule to the equipment object
    SET the equipment power per area based on user requirements

    PRINT a message indicating that internal loads are added for the space

# Set air change rate value based on user requirements

# Define infiltration schedule and its values based on user requirements
CREATE a ruleset schedule for infiltration
SET the name
GET the default day schedule from the ruleset
FOR each time and infiltration value pair from user requirements:
    ADD a value to the day schedule at the specified time with the specified infiltration setting

# For each space, assign infiltration parameters
FOR each space in the model:
    CREATE an infiltration object for the space
    SET the name
    ASSIGN the space to the infiltration object
    SET the air change rate using the defined value
    ASSIGN the infiltration schedule to the infiltration object
```

Answer:

```python
activity_level_schedule_type_limits = openstudio.model.ScheduleTypeLimits(model)
activity_level_schedule_type_limits.setName("Activity Level Schedule Type Limits")
activity_level_schedule_type_limits.setLowerLimitValue(0.0)
activity_level_schedule_type_limits.setUpperLimitValue(1000.0)
activity_level_schedule_type_limits.setNumericType("Continuous")
activity_level_schedule_type_limits.setUnitType("ActivityLevel")

activity_level_schedule = openstudio.model.ScheduleRuleset(model)
activity_level_schedule.setName("Activity Level Schedule")
activity_level_schedule.setScheduleTypeLimits(activity_level_schedule_type_limits)
activity_day_schedule = activity_level_schedule.defaultDaySchedule()
activity_day_schedule.addValue(openstudio.Time(0, 8, 0), 0.0)
activity_day_schedule.addValue(openstudio.Time(0, 17, 0), 80)
activity_day_schedule.addValue(openstudio.Time(0, 20, 0), 20)
activity_day_schedule.addValue(openstudio.Time(0, 24, 0), 0.0)

for space in model.getSpaces():

    people_def = openstudio.model.PeopleDefinition(model)
    people_def.setName(f"People Definition for {space.name().get()}")
    people_def.setNumberofPeople(73)
    people = openstudio.model.People(people_def)
    people.setSpace(space)
    people.setNumberofPeopleSchedule(default_schedule_set.numberofPeopleSchedule().get())
    people.setActivityLevelSchedule(activity_level_schedule)

    lights_def = openstudio.model.LightsDefinition(model)
    lights_def.setName(f"Lights Definition for {space.name().get()}")
    lights = openstudio.model.Lights(lights_def)
    lights.setSpace(space)
    lights.setSchedule(default_schedule_set.lightingSchedule().get())
    lights_def.setWattsperSpaceFloorArea(6.56)

    equip_def = openstudio.model.ElectricEquipmentDefinition(model)
    equip_def.setName(f"Equipment Definition for {space.name().get()}")
    equip = openstudio.model.ElectricEquipment(equip_def)
    equip.setSpace(space)
    equip.setSchedule(default_schedule_set.electricEquipmentSchedule().get())
    equip_def.setWattsperSpaceFloorArea(12.34)

    print(f"Internal loads added to space: {space.name().get()}")

ach_value = 1.49
```

```
infiltration_schedule = openstudio.model.ScheduleRuleset(model)
infiltration_schedule.setName("Infiltration Schedule")
day_schedule = infiltration_schedule.defaultDaySchedule()
day_schedule.addValue(openstudio.Time(0, 3, 0), 0.0)
day_schedule.addValue(openstudio.Time(0, 20, 0), 1)
day_schedule.addValue(openstudio.Time(0, 24, 0), 0.0)

for space in model.getSpaces():
    infiltration = openstudio.model.SpaceInfiltrationDesignFlowRate(model)
    infiltration.setName(f"Infiltration_{space.nameString()}")
    infiltration.setSpace(space)
    infiltration.setAirChangesperHour(ach_value)
    infiltration.setSchedule(infiltration_schedule)
```

Heating schedule correction

This step addresses the correction and refinement of the heating system's operational schedule based on actual activation time records provided in “Heating active time.csv.” The agent assigned to this task is the Programmer. The process involves programmatically parsing the recorded activation times for each date, extracting the first and last active time entries, and then reconstructing accurate daily heating schedules for model calibration. By mapping these corrected activation periods into the building simulation environment, the agent ensures that the heating system operation reflects real-world usage.

The dialogue with Programmer agent for generating corrected heating schedules based on actual activation time records:

Agent: Programmer (Consensus, gpt-4)

Files:

Heating active time.csv

System Prompt:

Use the provided pseudocode and information to generate executable Python code.

Example Response:

INITIALIZE an empty dictionary to store activation times

FOR each row in the data table:

    GET the date value from the appropriate column

    GET the first activation time value from the appropriate column

    GET the last activation time value from the appropriate column

    IF both the first and last activation time values are valid:

        PARSE the first activation time into hours and minutes

        PARSE the last activation time into hours and minutes

        CALCULATE the numerical representation of the first activation time

        CALCULATE the numerical representation of the last activation time

        STORE the activation time range for the date in the dictionary

Answer:

```
activation_times = {}
```

```python
for _, row in data.iterrows():
    date_str = row['Date']
    first_time_str = row['First Active Time']
    last_time_str = row['Last Active Time']
    if pd.notna(first_time_str) and pd.notna(last_time_str):
        first_hour, first_minute, _ = map(int, first_time_str.split(':'))
        last_hour, last_minute, _ = map(int, last_time_str.split(':'))
        first_time = first_hour - 1 + first_minute / 60.0
        last_time = last_hour + last_minute / 60.0
        activation_times[date_str] = (first_time, last_time)

default_day_schedule = radiator_schedule.defaultDaySchedule()
default_day_schedule.setName("All Other Days")
default_day_schedule.addValue(openstudio.Time(0, 24, 0), 0.0)

for date_str, (first_hour, last_hour) in activation_times.items():

    # Create schedule for correction
    daily_schedule = openstudio.model.ScheduleDay(model)
    daily_schedule.setName(f"Schedule for {date_str}")

    # Define schedule
    daily_schedule.addValue(openstudio.Time(0, int(first_hour), 0), 0.0)
    daily_schedule.addValue(openstudio.Time(0, int(last_hour), 0), 1.0)
    daily_schedule.addValue(openstudio.Time(0, 24, 0), 0.0)

    # Create ScheduleRule to apply the schedule to correction date
    date_rule = openstudio.model.ScheduleRule(radiator_schedule, daily_schedule)
    date_rule.setApplySunday(True)     # Apply rule to Sunday
    date_rule.setApplySaturday(True) # Apply rule to Saturday
    date_rule.setApplyMonday(True)     # Apply rule to Monday
    date_rule.setApplyTuesday(True)    # Apply rule to Tuesday
    date_rule.setApplyWednesday(True) # Apply rule to Wednesday
    date_rule.setApplyThursday(True)   # Apply rule to Thursday
    date_rule.setApplyFriday(True)      # Apply rule to Friday

    # Create Date object using year, month, day
    year, month, day = map(int, date_str.split('/'))
    rule_date = openstudio.Date(openstudio.MonthOfYear(month), day, year)
    date_rule.setStartDate(rule_date)
    date_rule.setEndDate(rule_date)
```

In this step, the file referenced is "Heating active time.csv", which provides a detailed schedule of the activation and deactivation times for the building's heating system over the course of a year. This document includes

specific data on the first active time and the last active time for each day, detailing the operational hours for the heating system.

Weather

In this section, the agent processes user-provided weather files, including both local climate data and design day specifications, to extract and ensure the use of accurate meteorological information. This enables precise simulation of building energy performance, incorporating location-specific and design-critical weather parameters.

Weather file extraction

This step involves identifying and specifying the appropriate local weather data file required for building energy simulations. The selected weather file, typically in .epw format, contains essential meteorological information such as temperature, humidity, solar radiation, wind speed, and location-specific data (e.g., latitude, longitude, calendar year). The agent responsible for this task, the Programmer, receives the user-provided weather file name and generates the absolute file path to ensure accurate referencing during simulation. This process guarantees that the model utilizes precise climatic inputs corresponding to the building's geographic context.

The dialogue with the Programmer agent for extracting and referencing the weather file:

Agent: Programmer (Consensus, gpt-4)

User Prompt:

The weather file name I provided is Weather.epw.

System Prompt:

This is a local weather file (e.g., .epw files) to incorporate geographical information such as latitude and longitude, and calendar year weather data. Please replace the Example name with the actual information based on the User requirement.

Example Response:

epw_file_path = os.path.abspath('ExampleName.epw')

Answer:

epw_file_path = os.path.abspath('Weather.epw')

Design day data extraction

This step focuses on selecting and specifying the design day weather file necessary for building performance simulations. The design day file, typically in .ddy format, contains representative climate data for critical days, including parameters such as temperature, humidity, and solar radiation, which are used for sizing HVAC systems and peak load calculations. The agent performing this task, the Programmer, receives the user-specified design day file name and outputs the file path, ensuring that the simulation references accurate and relevant climatic conditions for design purposes.

The dialogue with the Programmer agent for extracting and referencing the design day data file:

Agent: Programmer (Consensus, gpt-4)

User Prompt:

The weather file name I provided is DesignDay.ddy.

System Prompt:

This is a design day file (e.g., .ddy files) to incorporate climate data on the design days such as temperature, humidity, and solar radiation. Please replace the Example name with the actual information based on the User requirement.

Example Response:

ddy_file_path = 'ExampleName.ddy'

Answer:

```
ddy_file_path = 'DesignDay.ddy'
```

Simulation setting

In this section, the agent processes user-defined simulation parameters such as time steps, output variables, and reporting intervals and configures the simulation model accordingly. The outputs are integrated to generate a complete, executable simulation code, and the final .osm file is created, ensuring that all essential data for building energy simulations is accurately represented and ready for analysis.

Simulation model creation

This step involves translating user-defined simulation requirements into a structured simulation model within the building energy simulation environment. The Programmer agent interprets the specified outputs, such as desired output variables, time steps, and reporting intervals, and configures the simulation parameters accordingly. The agent generates programmatic instructions to set up the simulation environment, including timestep configuration, output variable selection, and output file settings, ensuring that the simulation aligns with the user's objectives.

The dialogue with the Programmer agent for simulation model creation based on user-defined requirements:

Agent: Programmer (Consensus, gpt-4)

User Prompt:

I want the simulation timestep to be one hour, and I want the output variable to be Boiler Heating Energy.

System Prompt:

Analyze user-defined requirements, including simulation outputs. Set up simulation parameters, such as output variables, time periods, and timestep intervals, to align with user requirements. Please only replace words with 'Example'. For example, only replace ExampleComponent. Do not delete .name().get()

Example Response:

SET the simulation timestep interval using ExampleValue

CREATE an output variable for 'Example_output' in the model

SET the reporting frequency for the output variable to 'Timestep'

SET the key value for the output variable using ExampleComponent.name().get()

CONFIGURE the output control files to generate output in CSV format

Answer:

```
timestep = model.getTimestep()
timestep.setNumberOfTimestepsPerHour(1)

output_variable = openstudio.model.OutputVariable('Boiler Heating Energy', model)
output_variable.setReportingFrequency('Timestep')
output_variable.setKeyValue(boiler.name().get())
```

```
output_control_files = model.getOutputControlFiles()
output_control_files.setOutputCSV(True)
```

Simulation code integration

In this section, the outputs generated by the agents are processed and integrated to produce a complete, executable simulation code. The integration step ensures that the generated code is not only syntactically correct but also operationally coherent, enabling the user to run simulations without encountering issues. The following shows the integrated code.

```
import ezdxf
import matplotlib.pyplot as plt
from collections import defaultdict
import openstudio
import datetime
import os
import subprocess
import json
import pandas as pd

model = openstudio.model.Model()
import ezdxf
from collections import defaultdict

filename = "60286028_E_DST_001"
doc = ezdxf.readfile(f"{filename}.dxf")

def round_point(point, precision=6):
    return tuple(round(coord, precision) for coord in point)

def calculate_signed_area(points):
    area = 0.0
    n = len(points)
    for i in range(n - 1):
        x1, y1 = points[i][0], points[i][1]
        x2, y2 = points[i + 1][0], points[i + 1][1]
        area += (x1 * y2 - x2 * y1)
    return area / 2.0

msp = doc.modelspace()
outline_entities = [entity for entity in msp if entity.dxf.layer == 'Outbound']
segments = []

for entity in outline_entities:
    if entity.dxftype() == 'LINE':
        start = tuple(entity.dxf.start[:2])
```

```
            end = tuple(entity.dxf.end[:2])
            segments.append((start, end))
        elif entity.dxftype() in ('LWPOLYLINE', 'POLYLINE'):
            if entity.dxftype() == 'LWPOLYLINE':
                points = [tuple(point[:2]) for point in entity.get_points()]
                is_closed = entity.closed
            else:   # POLYLINE
                points = [tuple(vertex.dxf.location[:2]) for vertex in entity.vertices()]
                is_closed = entity.is_closed

            for i in range(len(points) - 1):
                segments.append((points[i], points[i + 1]))
            if is_closed:
                segments.append((points[-1], points[0]))

point_map = {}
index = 0
adjacency = defaultdict(list)

for start, end in segments:
    start_rounded = round_point(start)
    end_rounded = round_point(end)

    if start_rounded not in point_map:
        point_map[start_rounded] = index
        index += 1
    if end_rounded not in point_map:
        point_map[end_rounded] = index
        index += 1

    idx_start = point_map[start_rounded]
    idx_end = point_map[end_rounded]

    adjacency[idx_start].append(idx_end)
    adjacency[idx_end].append(idx_start)

inverse_point_map = {v: k for k, v in point_map.items()}

def traverse_component(start_idx, adjacency, visited):
    ordered_indices = []
    current_idx = start_idx
    prev_idx = None
    while True:
        ordered_indices.append(current_idx)
```

```
            visited.add(current_idx)
            neighbors = adjacency[current_idx]
            next_idx = None
            for neighbor in neighbors:
                if neighbor != prev_idx:
                    next_idx = neighbor
                    break
            if next_idx is None or next_idx in visited:
                break
            prev_idx = current_idx
            current_idx = next_idx
        return ordered_indices

visited = set()
all_ordered_points = []

for idx in adjacency.keys():
    if idx not in visited:
        ordered_indices = traverse_component(idx, adjacency, visited)
        if ordered_indices[0] != ordered_indices[-1]:
            ordered_indices.append(ordered_indices[0])
        ordered_points = [inverse_point_map[i] for i in ordered_indices]

        area = calculate_signed_area(ordered_points)
        if area > 0:
            ordered_points.reverse()
            print("Point sequence reversed to ensure the normal points downward.")

        all_ordered_points.append(ordered_points)
import json
import openstudio

def set_building_envelope_with_values(model):
    # Wall material and construction
    wall_material = openstudio.model.MasslessOpaqueMaterial(model)
    wall_material.setName("Wall Material")
    wall_material.setThermalResistance(0.16)   # U-values (area weighted average)
    wall_material.setSolarAbsorptance(0.6)   # Solar Absorptance
    wall_material.setVisibleAbsorptance(0.6)   # Visible Absorptance
    wall_material.setThermalAbsorptance(0.9)   # Thermal Absorptance

    wall_construction = openstudio.model.Construction(model)
    wall_construction.setName("Wall Construction")
    wall_construction.insertLayer(0, wall_material)
```

```python
    # Roof material and construction
    roof_material = openstudio.model.MasslessOpaqueMaterial(model)
    roof_material.setName("Roof Material")
    roof_material.setThermalResistance(0.12)   # U-values (area weighted average)
    roof_material.setSolarAbsorptance(0.7)   # Solar Absorptance
    roof_material.setVisibleAbsorptance(0.7)   # Visible Absorptance
    roof_material.setThermalAbsorptance(0.9)   # Thermal Absorptance

    roof_construction = openstudio.model.Construction(model)
    roof_construction.setName("Roof Construction")
    roof_construction.insertLayer(0, roof_material)

    # Floor material and construction
    floor_material = openstudio.model.MasslessOpaqueMaterial(model)
    floor_material.setName("Floor Material")
    floor_material.setThermalResistance(0.22)   # U-values (area weighted average)
    floor_material.setSolarAbsorptance(0.83)   # Solar Absorptance
    floor_material.setVisibleAbsorptance(0.83)   # Visible Absorptance
    floor_material.setThermalAbsorptance(0.9)   # Thermal Absorptance

    floor_construction = openstudio.model.Construction(model)
    floor_construction.setName("Floor Construction")
    floor_construction.insertLayer(0, floor_material)

    # Window material and construction
    window_material = openstudio.model.SimpleGlazing(model)
    window_material.setName("Window Glazing")
    window_material.setUFactor(1.8)   # Overall U-value (including frame)
    window_material.setSolarHeatGainCoefficient(0.4)   # Solar heat gain coefficient general (g-value EN
410)
    window_material.setVisibleTransmittance(0.66)   # Light transmittance general

    window_construction = openstudio.model.Construction(model)
    window_construction.setName("Window Construction")
    window_construction.insertLayer(0, window_material)

    return wall_construction, window_construction, roof_construction, floor_construction
wall_construction, window_construction, roof_construction, floor_construction =
set_building_envelope_with_values(model)
def set_default_schedule_set(model):
    schedule_set = openstudio.model.DefaultScheduleSet(model)

    occupancy_schedule = openstudio.model.ScheduleRuleset(model)
```

```python
    occupancy_schedule.setName("Building Occupancy Schedule")
    occupancy_day_schedule = occupancy_schedule.defaultDaySchedule()
    occupancy_day_schedule.addValue(openstudio.Time(0, 8, 0), 0.0)
    occupancy_day_schedule.addValue(openstudio.Time(0, 18, 0), 1.0)
    occupancy_day_schedule.addValue(openstudio.Time(0, 24, 0), 0.0)
    schedule_set.setNumberofPeopleSchedule(occupancy_schedule)

    lighting_schedule = openstudio.model.ScheduleRuleset(model)
    lighting_schedule.setName("Building Lighting Schedule")
    lighting_day_schedule = lighting_schedule.defaultDaySchedule()
    lighting_day_schedule.addValue(openstudio.Time(0, 8, 0), 0.0)
    lighting_day_schedule.addValue(openstudio.Time(0, 18, 0), 1.0)
    lighting_day_schedule.addValue(openstudio.Time(0, 24, 0), 0.0)
    schedule_set.setLightingSchedule(lighting_schedule)

    equipment_schedule = openstudio.model.ScheduleRuleset(model)
    equipment_schedule.setName("Building Equipment Schedule")
    equipment_day_schedule = equipment_schedule.defaultDaySchedule()
    equipment_day_schedule.addValue(openstudio.Time(0, 8, 0), 0.0)    # Equipment off
    equipment_day_schedule.addValue(openstudio.Time(0, 18, 0), 1.0)   # Equipment on
    equipment_day_schedule.addValue(openstudio.Time(0, 24, 0), 0.0)   # Equipment off
    schedule_set.setElectricEquipmentSchedule(equipment_schedule)   # Set equipment schedule

    return schedule_set

def apply_thermal_zone_schedules(thermal_zone, model):
    # Create ScheduleTypeLimits for temperature setpoints
    temperature_schedule_type_limits = openstudio.model.ScheduleTypeLimits(model)
    temperature_schedule_type_limits.setName("Temperature Schedule Type Limits")
    temperature_schedule_type_limits.setLowerLimitValue(-60.0)
    temperature_schedule_type_limits.setUpperLimitValue(200.0)
    temperature_schedule_type_limits.setNumericType("Continuous")
    temperature_schedule_type_limits.setUnitType("Temperature")

    # Heating schedule
    heating_setpoint_schedule = openstudio.model.ScheduleRuleset(model)
    heating_setpoint_schedule.setName("Heating Setpoint Schedule")
    heating_setpoint_schedule.setScheduleTypeLimits(temperature_schedule_type_limits)
    heating_day_schedule = heating_setpoint_schedule.defaultDaySchedule()
    heating_day_schedule.addValue(openstudio.Time(0, 24, 0), 24)   # Heating setpoint

    # Cooling schedule
    ventilation_setpoint_schedule = openstudio.model.ScheduleRuleset(model)
    ventilation_setpoint_schedule.setName("Building Ventilation Schedule")
```

```
    ventilation_day_schedule = ventilation_setpoint_schedule.defaultDaySchedule()
    ventilation_day_schedule.addValue(openstudio.Time(0, 24, 0), 25)  # Ventilation setpoint

    # Assign heating and cooling schedules to the thermal zone
    thermostat_setpoint = openstudio.model.ThermostatSetpointDualSetpoint(model)
    thermostat_setpoint.setHeatingSchedule(heating_setpoint_schedule)
    thermostat_setpoint.setCoolingSchedule(ventilation_setpoint_schedule)
    thermal_zone.setThermostatSetpointDualSetpoint(thermostat_setpoint)
default_schedule_set = set_default_schedule_set(model)

space_type = openstudio.model.SpaceType(model)
space_type.setStandardsSpaceType('Office')
floor_height = 3.5
num_floors = 4

for idx, ordered_points in enumerate(all_ordered_points):
    # Remove the duplicate last point
    os_points = [openstudio.Point3d(pt[0], pt[1], 0.0) for pt in ordered_points[:-1]]

    # Create a Point3dVector
    point_vector = openstudio.Point3dVector(os_points)

    for floor in range(num_floors):
        z_offset = floor * floor_height  # Height offset for each floor
        translated_points = [openstudio.Point3d(pt.x(), pt.y(), z_offset) for pt in os_points]
        point_vector = openstudio.Point3dVector(translated_points)

        # Create space
        space = openstudio.model.Space.fromFloorPrint(point_vector, floor_height, model)
        if space.is_initialized():
            space = space.get()
            space.setName(f"Building Space {idx+1} Floor {floor+1}")
            space.setSpaceType(space_type)

            space.setDefaultScheduleSet(default_schedule_set)

            surfaces = space.surfaces()
            for surface in surfaces:
                if surface.surfaceType() == "Wall":
                    surface.setConstruction(wall_construction)

                    if surface.outsideBoundaryCondition() == "Outdoors":
                        wwr = 0.6
                        sill_height = 1.0
```

```
                        success = surface.setWindowToWallRatio(wwr, sill_height, True)
                        if success:
                            for sub_surface in surface.subSurfaces():
                                if sub_surface.subSurfaceType() == "FixedWindow":
                                    sub_surface.setConstruction(window_construction)
                elif surface.surfaceType() == "RoofCeiling":
                    surface.setConstruction(roof_construction)
                elif surface.surfaceType() == "Floor":
                    surface.setConstruction(floor_construction)



            # Create and assign a thermal zone
            thermal_zone = openstudio.model.ThermalZone(model)
            thermal_zone.setName(f"Building Thermal Zone {idx+1} Floor {floor+1}")
            space.setThermalZone(thermal_zone)

            print(f"Successfully created building space {idx+1} on floor {floor+1}.")

            apply_thermal_zone_schedules(thermal_zone, model)

        else:
            print(f"Failed to create building space {idx+1} on floor {floor+1}.")
activity_level_schedule_type_limits = openstudio.model.ScheduleTypeLimits(model)
activity_level_schedule_type_limits.setName("Activity Level Schedule Type Limits")
activity_level_schedule_type_limits.setLowerLimitValue(0.0)
activity_level_schedule_type_limits.setUpperLimitValue(1000.0)
activity_level_schedule_type_limits.setNumericType("Continuous")
activity_level_schedule_type_limits.setUnitType("ActivityLevel")

activity_level_schedule = openstudio.model.ScheduleRuleset(model)
activity_level_schedule.setName("Activity Level Schedule")
activity_level_schedule.setScheduleTypeLimits(activity_level_schedule_type_limits)
activity_day_schedule = activity_level_schedule.defaultDaySchedule()
activity_day_schedule.addValue(openstudio.Time(0, 8, 0), 0.0)
activity_day_schedule.addValue(openstudio.Time(0, 17, 0), 80)
activity_day_schedule.addValue(openstudio.Time(0, 20, 0), 20)
activity_day_schedule.addValue(openstudio.Time(0, 24, 0), 0.0)

for space in model.getSpaces():

    people_def = openstudio.model.PeopleDefinition(model)
    people_def.setName(f"People Definition for {space.name().get()}")
```

```python
    people_def.setNumberofPeople(73)
    people = openstudio.model.People(people_def)
    people.setSpace(space)
    people.setNumberofPeopleSchedule(default_schedule_set.numberofPeopleSchedule().get())
    people.setActivityLevelSchedule(activity_level_schedule)

    lights_def = openstudio.model.LightsDefinition(model)
    lights_def.setName(f"Lights Definition for {space.name().get()}")
    lights = openstudio.model.Lights(lights_def)
    lights.setSpace(space)
    lights.setSchedule(default_schedule_set.lightingSchedule().get())
    lights_def.setWattsperSpaceFloorArea(6.56)

    equip_def = openstudio.model.ElectricEquipmentDefinition(model)
    equip_def.setName(f"Equipment Definition for {space.name().get()}")
    equip = openstudio.model.ElectricEquipment(equip_def)
    equip.setSpace(space)
    equip.setSchedule(default_schedule_set.electricEquipmentSchedule().get())
    equip_def.setWattsperSpaceFloorArea(12.34)

    print(f"Internal loads added to space: {space.name().get()}")

ach_value = 1.49

infiltration_schedule = openstudio.model.ScheduleRuleset(model)
infiltration_schedule.setName("Infiltration Schedule")
day_schedule = infiltration_schedule.defaultDaySchedule()
day_schedule.addValue(openstudio.Time(0, 3, 0), 0.0)
day_schedule.addValue(openstudio.Time(0, 20, 0), 1)
day_schedule.addValue(openstudio.Time(0, 24, 0), 0.0)

for space in model.getSpaces():
    infiltration = openstudio.model.SpaceInfiltrationDesignFlowRate(model)
    infiltration.setName(f"Infiltration_{space.nameString()}")
    infiltration.setSpace(space)
    infiltration.setAirChangesperHour(ach_value)
    infiltration.setSchedule(infiltration_schedule)


hot_water_temp_schedule = openstudio.model.ScheduleRuleset(model)
hot_water_temp_schedule.setName("Hot Water Supply Temperature Schedule")
hot_water_temp_schedule.defaultDaySchedule().setName("Hot Water Supply Temp Default")
hot_water_temp_schedule.defaultDaySchedule().addValue(openstudio.Time(0, 24, 0), 75.0)
```

```python
radiator_schedule = openstudio.model.ScheduleRuleset(model)
radiator_schedule.setName("Radiator Operation Schedule")

# Hot Water Loop
hot_water_plant = openstudio.model.PlantLoop(model)
hot_water_plant.setName("Hot Water Loop")
for thermal_zone in model.getThermalZones():
	sizing_zone = openstudio.model.SizingZone(model, thermal_zone)
	sizing_zone.setZoneHeatingDesignSupplyAirTemperature(24)
	sizing_zone.setHeatingDesignAirFlowMethod("None")
sizing_plant = hot_water_plant.sizingPlant()
sizing_plant.setLoopType("Heating")
sizing_plant.setDesignLoopExitTemperature(75)
sizing_plant.setLoopDesignTemperatureDifference(10)

# Hot Water Nodes
hot_water_supply_outlet_node = hot_water_plant.supplyOutletNode()
hot_water_supply_inlet_node = hot_water_plant.supplyInletNode()

# Hot Water Pumps
hot_water_pump = openstudio.model.PumpVariableSpeed(model)
hot_water_pump.setName("Hot Water Loop Pump")
hot_water_pump.addToNode(hot_water_supply_inlet_node)

# Boiler
boiler = openstudio.model.BoilerHotWater(model)
boiler.setName("Hot Water Boiler")
boiler.setBoilerFlowMode("LeavingSetpointModulated")
boiler.setNominalThermalEfficiency(1)
boiler.setWaterOutletUpperTemperatureLimit(75)
boiler.setSizingFactor(1.25)
hot_water_plant.addSupplyBranchForComponent(boiler)
boiler.setNominalCapacity(240000)
boiler.setDesignWaterFlowRate(0.005)
boiler.setMinimumPartLoadRatio(0.15)
boiler.setMaximumPartLoadRatio(0.6)

# Setpoint Manager
setpoint_manager = openstudio.model.SetpointManagerScheduled(model, hot_water_temp_schedule)
setpoint_manager.setControlVariable("Temperature")
setpoint_manager.addToNode(hot_water_supply_outlet_node)

# Supply Bypass Pipes
supply_bypass_pipe = openstudio.model.PipeAdiabatic(model)
```

```
hot_water_plant.addSupplyBranchForComponent(supply_bypass_pipe)

# Demand Bypass Pipes
demand_bypass_pipe = openstudio.model.PipeAdiabatic(model)
hot_water_plant.addDemandBranchForComponent(demand_bypass_pipe)

# Radiators
for thermal_zone in model.getThermalZones():
    heating_coil = openstudio.model.CoilHeatingWaterBaseboard(model)
    heating_coil.setName(f"Heating Coil for {thermal_zone.name().get()}")
    heating_coil.setMaximumWaterFlowRate(0.001)
    heating_coil.setUFactorTimesAreaValue(500)
    radiator = openstudio.model.ZoneHVACBaseboardConvectiveWater(model, radiator_schedule,
heating_coil)
    radiator.setName(f"Radiator in {thermal_zone.name().get()}")
    radiator.addToThermalZone(thermal_zone)
    hot_water_plant.addDemandBranchForComponent(heating_coil)


data = pd.read_csv('model_generation_data.csv')

activation_times = {}
for _, row in data.iterrows():
    date_str = row['Date']
    first_time_str = row['First Active Time']
    last_time_str = row['Last Active Time']
    if pd.notna(first_time_str) and pd.notna(last_time_str):
        first_hour, first_minute, _ = map(int, first_time_str.split(':'))
        last_hour, last_minute, _ = map(int, last_time_str.split(':'))
        first_time = first_hour - 1 + first_minute / 60.0
        last_time = last_hour + last_minute / 60.0
        activation_times[date_str] = (first_time, last_time)

default_day_schedule = radiator_schedule.defaultDaySchedule()
default_day_schedule.setName("All Other Days")
default_day_schedule.addValue(openstudio.Time(0, 24, 0), 0.0)

for date_str, (first_hour, last_hour) in activation_times.items():

    # Create schedule for correction
    daily_schedule = openstudio.model.ScheduleDay(model)
    daily_schedule.setName(f"Schedule for {date_str}")

    # Define schedule
```

```
    daily_schedule.addValue(openstudio.Time(0, int(first_hour), 0), 0.0)
    daily_schedule.addValue(openstudio.Time(0, int(last_hour), 0), 1.0)
    daily_schedule.addValue(openstudio.Time(0, 24, 0), 0.0)

    # Create ScheduleRule to apply the schedule to correction date
    date_rule = openstudio.model.ScheduleRule(radiator_schedule, daily_schedule)
    date_rule.setApplySunday(True)     # Apply rule to Sunday
    date_rule.setApplySaturday(True) # Apply rule to Saturday
    date_rule.setApplyMonday(True)     # Apply rule to Monday
    date_rule.setApplyTuesday(True)    # Apply rule to Tuesday
    date_rule.setApplyWednesday(True) # Apply rule to Wednesday
    date_rule.setApplyThursday(True)   # Apply rule to Thursday
    date_rule.setApplyFriday(True)      # Apply rule to Friday

    # Create Date object using year, month, day
    year, month, day = map(int, date_str.split('/'))
    rule_date = openstudio.Date(openstudio.MonthOfYear(month), day, year)
    date_rule.setStartDate(rule_date)
    date_rule.setEndDate(rule_date)

epw_file_path = os.path.abspath('Weather.epw')

epw_path = openstudio.path(epw_file_path)
epw_file_option = openstudio.EpwFile.load(epw_path)

if epw_file_option.is_initialized():
    epw_file = epw_file_option.get()

    # Set Site Information First
    # Retrieve site information from the EPW file
    latitude = epw_file.latitude()
    longitude = epw_file.longitude()
    time_zone = epw_file.timeZone()
    elevation = epw_file.elevation()
    city = epw_file.city()
    stateProvinceRegion = epw_file.stateProvinceRegion()
    country = epw_file.country()

    # Set the Site information in the model
    site = model.getSite()

    # Use the city name as the Site name to avoid encoding issues
    site_name = city if city else 'Default Site Name'
    site.setName(site_name)
```

```python
site.setLatitude(latitude)
site.setLongitude(longitude)
site.setTimeZone(time_zone)
site.setElevation(elevation)
print(f"Site information has been set: {site.name().get()}")

# Set Weather File
# Set the weather file in the model
weather_file_option = openstudio.model.WeatherFile.setWeatherFile(model, epw_file)
if weather_file_option.is_initialized():
    weather_file = weather_file_option.get()
    print(f"Weather file '{epw_file_path}' has been successfully set.")

    # Update the YearDescription object
    year_description = model.getYearDescription()
    year_description.setCalendarYear(2018)

    # Retrieve the start day of the week from the EPW file
    epw_start_day_of_week = epw_file.startDayOfWeek()
    epw_start_day_of_week_str = epw_start_day_of_week.valueName()

    # Valid day names expected by OpenStudio
    valid_days = ["Sunday", "Monday", "Tuesday", "Wednesday", "Thursday", "Friday", "Saturday"]

    # Check if the day name is valid
    if epw_start_day_of_week_str in valid_days:
        year_description.setDayofWeekforStartDay(epw_start_day_of_week_str)
    else:
        print(f"Invalid start day '{epw_start_day_of_week_str}' in EPW file. Defaulting to 'Sunday'.")
        year_description.setDayofWeekforStartDay("Sunday")

    # Update the RunPeriod object
    run_period = model.getRunPeriod()
    run_period.setBeginMonth(epw_file.startDate().monthOfYear().value())
    run_period.setBeginDayOfMonth(epw_file.startDate().dayOfMonth())
    run_period.setEndMonth(epw_file.endDate().monthOfYear().value())
    run_period.setEndDayOfMonth(epw_file.endDate().dayOfMonth())

    # Use holidays and daylight saving time from the weather file
    run_period.setUseWeatherFileHolidays(True)
    run_period.setUseWeatherFileDaylightSavings(True)

    # Check the WeatherFile object in the model
```

```
        weather_file = model.getWeatherFile()
        url = weather_file.url()

        if url.is_initialized():
            print("WeatherFile object exists in the model.")
            print(f"Weather file URL: {url.get()}")
        else:
            print("Warning: No WeatherFile object found in the model.")
    else:
        print(f"Failed to set weather file in the model.")
else:
    print(f"Failed to load EPW file '{epw_file_path}'.")

ddy_file_path = 'DesignDay.ddy'

ddy_workspace = openstudio.Workspace.load(openstudio.path(ddy_file_path))
if ddy_workspace.is_initialized():
    ddy_workspace = ddy_workspace.get()

    # Use ReverseTranslator to convert the workspace into an OpenStudio model
    rt = openstudio.energyplus.ReverseTranslator()
    ddy_model = rt.translateWorkspace(ddy_workspace)

    # Retrieve the design day objects from the translated model
    design_days = ddy_model.getDesignDays()

    # Add the design day objects to the main model
    for design_day in design_days:
        model.addObject(design_day.clone())
    print(f"Design days from '{ddy_file_path}' have been added to the model.")
else:
    print(f"Failed to load design days from '{ddy_file_path}'.")

timestep = model.getTimestep()
timestep.setNumberOfTimestepsPerHour(1)

boiler_heating_energy_output = openstudio.model.OutputVariable('Boiler Heating Energy', model)
boiler_heating_energy_output.setReportingFrequency('Daily')
boiler_heating_energy_output.setKeyValue(boiler.name().get())

output_control_files = model.getOutputControlFiles()
output_control_files.setOutputCSV(True)

# Set timestep
```

```python
timestep = model.getTimestep()
timestep.setNumberOfTimestepsPerHour(1)

# Set output
Boiler_Heating_Energy_output = openstudio.model.OutputVariable('Boiler Heating Energy', model)
Boiler_Heating_Energy_output.setReportingFrequency('Daily')
Boiler_Heating_Energy_output.setKeyValue(boiler.name().get())

# Other Output...
# Set OutputControl:Files
output_control_files = model.getOutputControlFiles()
output_control_files.setOutputCSV(True)

if not model.isValid():
    print("Model validation error: The model is invalid and may contain incomplete or incorrect objects. 
Please check the model.")
else:
    # Get the current time and format it as a string
    current_time = datetime.datetime.now().strftime("%Y%m%d_%H%M%S")

    # Construct a filename with a timestamp
    save_path = f'Openstudiomodel.osm'

    # Save the model
    model.save(openstudio.path(save_path), True)
    print(f"Model has been saved to {save_path}")

    # Save the model path to a file for use by another script
    with open('model_info.txt', 'w') as info_file:
        info_file.write(f"{save_path}")
        info_file.write(f"{current_time}")
```

OSM file generation

After executing the integrated simulation code, an OpenStudio .osm file is generated. This file serves as the foundation for building energy simulation and can be opened and analyzed using the OpenStudio software. The .osm file includes all the necessary data about the building geometry, systems, schedules, and materials, which are essential for running simulations in EnergyPlus or other compatible simulation engines.

Data S4-Model calibration

Calibration parameters integration

In this section, the agent processes user-defined calibration parameters, extracts necessary data, and creates parameter range assignments, ensuring that all critical variables such as heating setpoints, equipment power densities, and infiltration rates are accurately structured. This provides a solid foundation for subsequent calibration and energy modeling tasks.

Calibration parameters analysis

This step focuses on extracting and organizing key calibration parameters and their respective ranges from user requirements to support building energy model calibration. The agent responsible for this task, the Information Retriever, systematically identifies and structures essential variables such as heating setpoint, equipment and lighting power density, occupancy, infiltration rate, boiler characteristics, and heating coil performance. The output is presented in a standardized JSON format.

The dialogue with the Information Retriever agent for extracting calibration parameter ranges based on user requirements:

Agent: Information Retriever (Consensus, gpt-4)

User Prompt:

We intend to select the following parameters as key identification variables, each within their specified ranges: the heating setpoint will range from 18°C to 24°C; equipment power density will vary between 3 W/m² and 22 W/m²; lighting power density is set from 1.5 W/m² to 15 W/m². The number of people accommodated will range from 30 to 200, adjusted based on building area and office scenarios. Infiltration air changes per hour (ACH) will be set between 0.5 and 1.5, tailored to actual conditions. Additionally, the boiler design water flow rate will range from 0.0001 m³/s to 0.005 m³/s, the boiler minimum part load ratio will be between 0.1 and 0.4, and the boiler maximum part load ratio will vary from 0.6 to 0.9. The heating coil U-factor multiplied by area value will be set between 500.0 W/K and 2000.0 W/K, and the heating coil maximum water flow rate will range from 0.001 m³/s to 0.006 m³/s.

System Prompt:

Extract information on calibration parameters and their ranges from User requirement. Please provide the results in a JSON format.

Example Response:

JSON format

Answer:


```
{
    "calibration_parameters": [
        {
            "name": "heating setpoint",
            "range": {
                "min": 18,
                "max": 24,
                "unit": "°C"
            }
        },
        {
            "name": "equipment power density",
```

```
        "range": {
            "min": 3,
            "max": 22,
            "unit": "W/m²"
        }
    },
    {
        "name": "lighting power density",
        "range": {
            "min": 1.5,
            "max": 15,
            "unit": "W/m²"
        }
    },
    {
        "name": "number of people accommodated",
        "range": {
            "min": 30,
            "max": 200
        }
    },
    {
        "name": "infiltration air changes per hour",
        "range": {
            "min": 0.5,
            "max": 1.5,
            "unit": "ACH"
        }
    },
    {
        "name": "boiler design water flow rate",
        "range": {
            "min": 0.0001,
            "max": 0.005,
            "unit": "m³/s"
        }
    },
    {
        "name": "boiler minimum part load ratio",
        "range": {
            "min": 0.1,
            "max": 0.4
        }
    },
```

```
        {
            "name": "boiler maximum part load ratio",
            "range": {
                "min": 0.6,
                "max": 0.9
            }
        },
        {
            "name": "heating coil U-factor multiplied by area value",
            "range": {
                "min": 500.0,
                "max": 2000.0,
                "unit": "W/K"
            }
        },
        {
            "name": "heating coil maximum water flow rate",
            "range": {
                "min": 0.001,
                "max": 0.006,
                "unit": "m³/s"
            }
        }
    ]
}
```


The dialogue with Reviewer agent for evaluating whether the information extracted is accurate:
Agent: Reviewer (Consensus, gpt-4)

Files:

User Requirement of "Calibration parameters analysis"
extracted_calibration_parameter_information.json

Answer:

- Entry: Calibration parameters for "heating setpoint"
- Confidence: 1
- Justification: The original information states that the heating setpoint will range from 18°C to 24°C, which matches the LLM output.

- Entry: Calibration parameters for "equipment power density"
- Confidence: 1
- Justification: The original information states that the equipment power density will vary between 3 W/m² and 22 W/m², which matches the LLM output.

- Entry: Calibration parameters for "lighting power density"

- Confidence: 1
- Justification: The original information states that the lighting power density is set from 1.5 W/m² to 15 W/m², which matches the LLM output.

- Entry: Calibration parameters for "number of people accommodated"
- Confidence: 1
- Justification: The original information states that the number of people accommodated will range from 30 to 200, which matches the LLM output.

- Entry: Calibration parameters for "infiltration air changes per hour"
- Confidence: 1
- Justification: The original information states that the infiltration air changes per hour (ACH) will be set between 0.5 and 1.5, which matches the LLM output.

- Entry: Calibration parameters for "boiler design water flow rate"
- Confidence: 1
- Justification: The original information states that the boiler design water flow rate will range from 0.0001 m³/s to 0.005 m³/s, which matches the LLM output.

- Entry: Calibration parameters for "boiler minimum part load ratio"
- Confidence: 1
- Justification: The original information states that the boiler minimum part load ratio will be between 0.1 and 0.4, which matches the LLM output.

- Entry: Calibration parameters for "boiler maximum part load ratio"
- Confidence: 1
- Justification: The original information states that the boiler maximum part load ratio will vary from 0.6 to 0.9, which matches the LLM output.

- Entry: Calibration parameters for "heating coil U-factor multiplied by area value"
- Confidence: 1
- Justification: The original information states that the heating coil U-factor multiplied by area value will be set between 500.0 W/K and 2000.0 W/K, which matches the LLM output.

- Entry: Calibration parameters for "heating coil maximum water flow rate"
- Confidence: 1

Justification: The original information states that the heating coil maximum water flow rate will range from 0.001 m³/s to 0.006 m³/s, which matches the LLM output.

Calibration parameters creation

This step focuses on the automated creation of calibration parameters required for building energy model calibration. The Programmer agent takes as input a JSON file containing extracted calibration parameter information and a model library in Excel format. Guided by system prompts, the agent identifies and selects the appropriate measure code snippets for each calibration parameter directly from the model library, and then combines these snippets to generate a complete code set. The process ensures that all relevant measures are

correctly integrated according to the input parameters, without modifying the original code, defining extracted content, or importing libraries.

Dialogue with Programmer agent for generating calibration parameter code from extracted data and model library:

Agent: Programmer (Consensus, gpt-4)

Files:

extracted_calibration_parameter_information.json

Model library.xlsx

System Prompt:

Using the calibration parameter data provided in the JSON file, select the corresponding measure code snippets for each measure from the model library. Combine these code snippets to generate a complete code, ensuring that all measures are integrated.

Example Response:

FOR each calibration measure required:

SET measure_name to the name of the ExampleMeasure

SET measure_path to the path for ExampleMeasure's code file

CREATE a spec object from the measure path and measure name

CREATE a measure module object from the spec

LOAD the module using the loader

GET the ExampleMeasure class from the loaded module

Answer:

```
# Code to generate the complete code by combining the measure code snippets for each measure

measure_name_hsp = "AdjustHeatingSetpointMeasure"
measure_path_hsp = pathlib.Path(__file__).parent / "measures" / measure_name_hsp / "measure.py"
spec_hsp = importlib.util.spec_from_file_location(measure_name_hsp, measure_path_hsp)
measure_module_hsp = importlib.util.module_from_spec(spec_hsp)
spec_hsp.loader.exec_module(measure_module_hsp)
AdjustHeatingSetpointMeasure = measure_module_hsp.AdjustHeatingSetpointMeasure

measure_name_epd = "AdjustEquipmentPowerDensityMeasure"
measure_path_epd = pathlib.Path(__file__).parent / "measures" / measure_name_epd / "measure.py"
spec_epd = importlib.util.spec_from_file_location(measure_name_epd, measure_path_epd)
measure_module_epd = importlib.util.module_from_spec(spec_epd)
spec_epd.loader.exec_module(measure_module_epd)
AdjustEquipmentPowerDensityMeasure = measure_module_epd.AdjustEquipmentPowerDensityMeasure

measure_name_lpd = "AdjustLightingPowerDensityMeasure"
measure_path_lpd = pathlib.Path(__file__).parent / "measures" / measure_name_lpd / "measure.py"
spec_lpd = importlib.util.spec_from_file_location(measure_name_lpd, measure_path_lpd)
measure_module_lpd = importlib.util.module_from_spec(spec_lpd)
spec_lpd.loader.exec_module(measure_module_lpd)
```

```
AdjustLightingPowerDensityMeasure = measure_module_lpd.AdjustLightingPowerDensityMeasure

measure_name_pn = "AdjustPeopleNumberMeasure"
measure_path_pn = pathlib.Path(__file__).parent / "measures" / measure_name_pn / "measure.py"
spec_pn = importlib.util.spec_from_file_location(measure_name_pn, measure_path_pn)
measure_module_pn = importlib.util.module_from_spec(spec_pn)
spec_pn.loader.exec_module(measure_module_pn)
AdjustPeopleNumberMeasure = measure_module_pn.AdjustPeopleNumberMeasure

measure_name_infiltration = "AdjustInfiltrationRateMeasure"
measure_path_infiltration = pathlib.Path(__file__).parent / "measures" / measure_name_infiltration /
"measure.py"
spec_infiltration = importlib.util.spec_from_file_location(measure_name_infiltration, measure_path_infiltration)
measure_module_infiltration = importlib.util.module_from_spec(spec_infiltration)
spec_infiltration.loader.exec_module(measure_module_infiltration)
AdjustInfiltrationRateMeasure = measure_module_infiltration.AdjustInfiltrationRateMeasure

measure_name_boiler_flow = "AdjustBoilerDesignWaterFlowRateMeasure"
measure_path_boiler_flow = pathlib.Path(__file__).parent / "measures" / measure_name_boiler_flow /
"measure.py"
spec_boiler_flow = importlib.util.spec_from_file_location(measure_name_boiler_flow,
measure_path_boiler_flow)
measure_module_boiler_flow = importlib.util.module_from_spec(spec_boiler_flow)
spec_boiler_flow.loader.exec_module(measure_module_boiler_flow)
AdjustBoilerDesignWaterFlowRateMeasure =
measure_module_boiler_flow.AdjustBoilerDesignWaterFlowRateMeasure

measure_name_boiler_min_plr = "AdjustBoilerMinimumPartLoadRatioMeasure"
measure_path_boiler_min_plr = pathlib.Path(__file__).parent / "measures" / measure_name_boiler_min_plr /
"measure.py"
spec_boiler_min_plr = importlib.util.spec_from_file_location(measure_name_boiler_min_plr,
measure_path_boiler_min_plr)
measure_module_boiler_min_plr = importlib.util.module_from_spec(spec_boiler_min_plr)
spec_boiler_min_plr.loader.exec_module(measure_module_boiler_min_plr)
AdjustBoilerMinimumPartLoadRatioMeasure =
measure_module_boiler_min_plr.AdjustBoilerMinimumPartLoadRatioMeasure

measure_name_boiler_max_plr = "AdjustBoilerMaximumPartLoadRatioMeasure"
measure_path_boiler_max_plr = pathlib.Path(__file__).parent / "measures" / measure_name_boiler_max_plr /
"measure.py"
spec_boiler_max_plr = importlib.util.spec_from_file_location(measure_name_boiler_max_plr,
measure_path_boiler_max_plr)
measure_module_boiler_max_plr = importlib.util.module_from_spec(spec_boiler_max_plr)
spec_boiler_max_plr.loader.exec_module(measure_module_boiler_max_plr)
```

```
AdjustBoilerMaximumPartLoadRatioMeasure =
measure_module_boiler_max_plr.AdjustBoilerMaximumPartLoadRatioMeasure

measure_name_coil_ua = "AdjustHeatingCoilUFactorTimesAreaMeasure"
measure_path_coil_ua = pathlib.Path(__file__).parent / "measures" / measure_name_coil_ua / "measure.py"
spec_coil_ua = importlib.util.spec_from_file_location(measure_name_coil_ua, measure_path_coil_ua)
measure_module_coil_ua = importlib.util.module_from_spec(spec_coil_ua)
spec_coil_ua.loader.exec_module(measure_module_coil_ua)
AdjustHeatingCoilUFactorTimesAreaMeasure =
measure_module_coil_ua.AdjustHeatingCoilUFactorTimesAreaMeasure

measure_name_coil_max_flow = "AdjustHeatingCoilMaximumWaterFlowRateMeasure"
measure_path_coil_max_flow = pathlib.Path(__file__).parent / "measures" / measure_name_coil_max_flow /
"measure.py"
spec_coil_max_flow = importlib.util.spec_from_file_location(measure_name_coil_max_flow,
measure_path_coil_max_flow)
measure_module_coil_max_flow = importlib.util.module_from_spec(spec_coil_max_flow)
spec_coil_max_flow.loader.exec_module(measure_module_coil_max_flow)
AdjustHeatingCoilMaximumWaterFlowRateMeasure =
measure_module_coil_max_flow.AdjustHeatingCoilMaximumWaterFlowRateMeasure
```

Parameter range assignment

This step focuses on assigning appropriate value ranges to critical calibration parameters required for subsequent building performance simulations. The Programmer agent utilizes extracted parameter range data from the structured JSON file (extracted_calibration_parameter_information.json) and translates these into code-ready variable assignments. The system prompt instructs the agent to write parameter range definitions according to a specified format, omitting file loading, library imports, and iterative constructs, thereby producing clean and reusable code snippets. The resulting parameter assignments serve as standardized constraints for further calibration and modeling processes.

The dialogue with Programmer agent for parameter range assignment based on extracted calibration parameter information:

Agent: Programmer (Consensus, gpt-4)

Files:

extracted_calibration_parameter_information.json

System Prompt:

Using the extracted parameter ranges from json document, write the code according to the example response.

Example Response:

parameter_name_range = (lower_bound_value, upper_bound_value)

Answer:

```
# Define the parameter ranges
heating_setpoint_range = (18, 24)
equipment_power_density_range = (3, 22)
lighting_power_density_range = (1.5, 15)
```

```
number_of_people_accommodated_range = (30, 200)
infiltration_air_changes_per_hour_range = (0.5, 1.5)
boiler_design_water_flow_rate_range = (0.0001, 0.005)
boiler_minimum_part_load_ratio_range = (0.1, 0.4)
boiler_maximum_part_load_ratio_range = (0.6, 0.9)
heating_coil_U_factor_multiplied_by_area_value_range = (500.0, 2000.0)
heating_coil_maximum_water_flow_rate_range = (0.001, 0.006)
```

Optimization algorithm configuration

In this section, the agent processes user-defined genetic algorithm attributes, such as crossover, mutation, and selection strategies, to extract, organize, and register these settings into a structured format.

Genetic algorithm attribute assignment

This step focuses on extracting and organizing the specific configuration attributes of the genetic algorithm, as required by the user, from the provided input. The Information Retriever agent processes the user's requirements related to genetic algorithm operations, such as crossover, mutation, and selection strategies, along with their associated parameters. The extracted information is then formatted into a structured JSON output, ensuring clarity and consistency for subsequent computational procedures. This step guarantees that all relevant genetic algorithm settings, including crossover type and factors, mutation parameters, and selection methods, are clearly documented and readily accessible for further model implementation.

The dialogue with the Retriever agent for extracting genetic algorithm attributes from user specifications:

Agent: Information Retriever (Consensus, gpt-4)

User Prompt:

I require the genetic algorithm to register a blend crossover with a blending factor (alpha) of 0.5, a Gaussian mutation with a mean (mu) of 0, a standard deviation (sigma) of 1, and an independent probability (indpb) of 1.0 for each gene, as well as a tournament selection method with a tournament size (tournsize) of 3 for selecting individuals in the next generation.

System Prompt:

Extract the corresponding genetic algorithm attributes from the user-provided information. Compile all the retrieved attributes into a JSON format file.

Example Response:

JSON format

Answer:


```
{
    "genetic_algorithm": {
        "crossover": {
            "type": "blend",
            "alpha": 0.5
        },
        "mutation": {
            "type": "Gaussian",
            "mu": 0,
            "sigma": 1,
```

```
            "indpb": 1.0
        },
        "selection": {
            "method": "tournament",
            "tournsize": 3
        }
    }
}
```

The dialogue with Reviewer agent for evaluating whether the information extracted is accurate:
Agent: Reviewer (Consensus, gpt-4)

Files:

User Requirement of "Genetic algorithm attribute assignment"
extracted_genetic_algorithm_attribute.json
Answer:

- Entry: Crossover type and alpha value
- Confidence: 1
- Justification: The original information states that the user requires a blend crossover with a blending factor (alpha) of 0.5. This matches the LLM output.

- Entry: Mutation type, mu, sigma, and indpb values
- Confidence: 1
- Justification: The original information specifies a Gaussian mutation with a mean (mu) of 0, a standard deviation (sigma) of 1, and an independent probability (indpb) of 1.0 for each gene. This is accurately reflected in the LLM output.

- Entry: Selection type and tournsize value
- Confidence: 1

Justification: The original information mentions a tournament selection method with a tournament size (tournsize) of 3. This is correctly represented in the LLM output.

Register genetic operations

This step focuses on the automated registration of genetic algorithm operations required for parameter optimization tasks. The Programmer agent parses the attribute information defined in the provided script (Code for Parameter range assignment.py), ensuring each variable is correctly linked to its corresponding range and random generation method. The agent follows the structure outlined in the example response, carefully matching naming conventions and maintaining consistency with the parameter definitions. The output consists of Python code segments that register both the attribute generators and the initialization of individuals for the evolutionary algorithm.

The dialogue with Programmer agent for registering genetic operations based on parameter range assignment:

Agent: Programmer (Consensus, gpt-4)

Files:

Code for Parameter range assignment.py

System Prompt:

Based on the attribute information provided in the Code for Parameter range assignment.py, complete the corresponding code for in Example Response section. Ensure the code follows the exact structure and format of the Example Response. Maintain consistency in naming conventions between those in Parameter range assignment and the generated code.

Example Response:

FOR each attribute in the parameter range assignment:

    REGISTER the attribute with the toolbox using a random function and the parameter range

REGISTER 'individual' with the toolbox, using an initialization method,

    passing creator.Individual and a tuple of all registered attribute functions,

    with n set to a given value

Answer:

```
toolbox = base.Toolbox()
toolbox.register("clone", copy.deepcopy)

if not hasattr(creator, "FitnessMin"):
    creator.create("FitnessMin", base.Fitness, weights=(-1.0,))
if not hasattr(creator, "Individual"):
    creator.create("Individual", list, fitness=creator.FitnessMin)

# Registering the attributes with their respective ranges
toolbox.register('heating_setpoint', random.uniform, *heating_setpoint_range)
toolbox.register('equipment_power_density', random.uniform, *equipment_power_density_range)
toolbox.register('lighting_power_density', random.uniform, *lighting_power_density_range)
toolbox.register('number_of_people_accommodated', random.randint,
*number_of_people_accommodated_range)
toolbox.register('infiltration_air_changes_per_hour', random.uniform,
*infiltration_air_changes_per_hour_range)
toolbox.register('boiler_design_water_flow_rate', random.uniform, *boiler_design_water_flow_rate_range)
toolbox.register('boiler_minimum_part_load_ratio', random.uniform, *boiler_minimum_part_load_ratio_range)
toolbox.register('boiler_maximum_part_load_ratio', random.uniform, *boiler_maximum_part_load_ratio_range)
toolbox.register('heating_coil_U_factor_multiplied_by_area_value', random.uniform,
*heating_coil_U_factor_multiplied_by_area_value_range)
toolbox.register('heating_coil_maximum_water_flow_rate', random.uniform,
*heating_coil_maximum_water_flow_rate_range)

# Registering the individual with the attributes
toolbox.register('individual', tools.initCycle, creator.Individual,
                 (toolbox.heating_setpoint,
                  toolbox.equipment_power_density,
                  toolbox.lighting_power_density,
                  toolbox.number_of_people_accommodated,
```

```
                  toolbox.infiltration_air_changes_per_hour,
                  toolbox.boiler_design_water_flow_rate,
                  toolbox.boiler_minimum_part_load_ratio,
                  toolbox.boiler_maximum_part_load_ratio,
                  toolbox.heating_coil_U_factor_multiplied_by_area_value,
                  toolbox.heating_coil_maximum_water_flow_rate), n=1)
```

Individuals and population definition

This step focuses on defining the individuals and population for the genetic algorithm implementation. The Programmer agent utilizes extracted attribute values from the genetic algorithm configuration JSON file (extracted_genetic_algorithm_attribute.json) to set up the initialization and genetic operations. Key functions, such as population creation, evaluation, crossover (mate), mutation, and selection strategies, are registered using the extracted parameter values. This ensures that the genetic algorithm is initialized consistently with the intended design specifications, providing a reliable foundation for evolutionary optimization in subsequent modeling tasks.

The dialogue with Programmer agent for defining individuals and population based on extracted genetic algorithm attributes:

Agent: Programmer (Consensus, gpt-4)

Files:

extracted_genetic_algorithm_attribute.json

System Prompt:

Replace the "extracted actual numeric value" part with the extracted attribute values from json document in the example response.

Example Response:

REGISTER 'population' using the initialization function with the individual definition

REGISTER 'evaluate' using an evaluation function for individual

REGISTER 'mate' using a crossover method, setting method parameters to the extracted attribute values

REGISTER 'mutate' using a mutation method, setting method parameters to the extracted attribute values

REGISTER 'select' using a selection method, setting method parameters to the extracted attribute values

Answer:

```
toolbox.register('population', tools.initRepeat, list, toolbox.individual)
toolbox.register('evaluate', lambda ind: evaluate(ind))
toolbox.register('mate', tools.cxBlend, alpha=0.5)
toolbox.register('mutate', tools.mutGaussian, mu=0, sigma=1, indpb=1.0)
toolbox.register('select', tools.selTournament, tournsize=3)
```

Evaluation function definition

Users can interact with the LLM-agent integrated into the evaluation function definition module to intuitively solve tasks in this section. The interface for evaluation function setup, where users directly engage with the agent, is shown in Figure S21. In this section, the agent processes user-defined calibration parameters, applies building calibration measures, and calculates fitness by comparing simulation results with actual measured data.

Model initialization

This step initiates the model setup process by programmatically mapping extracted calibration parameter names from the input JSON file to explicit variables within the simulation code. The Programmer agent is responsible for replacing the placeholder names in the template code with the actual calibration parameter names, ensuring that all relevant parameters are correctly referenced line by line. No additional code beyond the required substitutions is introduced, and parameters are not defined or initialized within this step. This guarantees consistency and reproducibility when initializing model simulations with precise parameter assignments.

The dialogue with Programmer agent for initializing the model using extracted calibration parameters:

Agent: Programmer (Consensus, gpt-4)

Files:

extracted_calibration_parameter_information.json

System Prompt:

Using the calibration parameter data provided in the JSON file, replace the placeholders in the example response with the corresponding real names.

Example Response:

```
calibration_parameter_1 = individual[0]
calibration_parameter_2 = individual[1]
calibration_parameter_3 = individual[2]
...
sim_output_dir = join the output directory path with a generated unique simulation identifier
IF the simulation output directory does not exist:
    CREATE the simulation output directory

param_str = (
    f'calibration_parameter_1_' with its formatted value
    f'calibration_parameter_2_' with its formatted value
    f'calibration_parameter_3_' with its formatted value
    ...
)
```

Answer:

```
heating_setpoint = individual[0]
equipment_power_density = individual[1]
lighting_power_density = individual[2]
number_of_people_accommodated = individual[3]
infiltration_air_changes_per_hour = individual[4]
boiler_design_water_flow_rate = individual[5]
boiler_minimum_part_load_ratio = individual[6]
boiler_maximum_part_load_ratio = individual[7]
heating_coil_U_factor_multiplied_by_area_value = individual[8]
heating_coil_maximum_water_flow_rate = individual[9]

sim_output_dir = os.path.join(output_directory, f'sim_{uuid.uuid4().hex}')
if not os.path.exists(sim_output_dir):
```

```
    os.makedirs(sim_output_dir)
param_str = (
    f'heating_setpoint_{round(heating_setpoint, 2)}_'
    f'equipment_power_density_{round(equipment_power_density, 2)}_'
    f'lighting_power_density_{round(lighting_power_density, 2)}_'
    f'number_of_people_accommodated_{round(number_of_people_accommodated, 2)}_'
    f'infiltration_air_changes_per_hour_{round(infiltration_air_changes_per_hour, 2)}_'
    f'boiler_design_water_flow_rate_{round(boiler_design_water_flow_rate, 2)}_'
    f'boiler_minimum_part_load_ratio_{round(boiler_minimum_part_load_ratio, 2)}_'
    f'boiler_maximum_part_load_ratio_{round(boiler_maximum_part_load_ratio, 2)}_'

f'heating_coil_U_factor_multiplied_by_area_value_{round(heating_coil_U_factor_multiplied_by_area_value,
2)}_'
    f'heating_coil_maximum_water_flow_rate_{round(heating_coil_maximum_water_flow_rate, 2)}_'
)
```

Measure application

This step involves the automated application of building calibration measures based on previously extracted parameter data. The Programmer agent is responsible for selecting the relevant measure code snippets from the model library, as indicated by the calibration parameters provided in the extracted_calibration_parameter_information.json file. Each measure (e.g., heating setpoint adjustment, equipment and lighting power density, infiltration rate, boiler settings) is directly mapped to its corresponding code without modification or iteration. The agent integrates these code snippets to form a complete, executable workflow, ensuring all measures are properly configured and ready for subsequent simulation.

The dialogue with Programmer agent for automated integration of building calibration measures using extracted parameter data:

Agent: Programmer (Consensus, gpt-4)

Files:

extracted_calibration_parameter_information.json

System Prompt:

Using the calibration parameter data provided in the JSON file, select the corresponding measure code snippets for each measure from the model library. Combine these code snippets to generate a complete code, ensuring that all measures are integrated. Please directly take the measure code from the model library, without modifying or writing iterations.

Example Response:

CREATE an instance of ExampleMeasure

GET the arguments for ExampleMeasure using the model

CREATE an argument map for ExampleMeasure

CLONE the required argument for ExampleMeasure

SET the value of the cloned argument using the corresponding calibration parameter

ASSIGN the cloned argument to the argument map with the correct key

APPEND the tuple of (ExampleMeasure, argument map) to the list of measures

Answer:

```
runner = openstudio.measure.OSRunner(openstudio.WorkflowJSON())
measures = []

# Code Snippets for each measure

measure_hsp = AdjustHeatingSetpointMeasure()
args_hsp = measure_hsp.arguments(model)
arg_map_hsp = openstudio.measure.OSArgumentMap()
hsp_arg = args_hsp[0].clone()
hsp_arg.setValue(heating_setpoint)
arg_map_hsp["heating_setpoint"] = hsp_arg
measures.append((measure_hsp, arg_map_hsp))

measure_epd = AdjustEquipmentPowerDensityMeasure()
args_epd = measure_epd.arguments(model)
arg_map_epd = openstudio.measure.OSArgumentMap()
epd_arg = args_epd[0].clone()
epd_arg.setValue(equipment_power_density)
arg_map_epd["epd_value"] = epd_arg
measures.append((measure_epd, arg_map_epd))

measure_lpd = AdjustLightingPowerDensityMeasure()
args_lpd = measure_lpd.arguments(model)
arg_map_lpd = openstudio.measure.OSArgumentMap()
lpd_arg = args_lpd[0].clone()
lpd_arg.setValue(lighting_power_density)
arg_map_lpd["lpd_value"] = lpd_arg
measures.append((measure_lpd, arg_map_lpd))

measure_pn = AdjustPeopleNumberMeasure()
args_pn = measure_pn.arguments(model)
arg_map_pn = openstudio.measure.OSArgumentMap()
number_of_people_arg = args_pn[0].clone()
number_of_people_arg.setValue(number_of_people_int)
arg_map_pn["number_of_people"] = number_of_people_arg
measures.append((measure_pn, arg_map_pn))

measure_infiltration = AdjustInfiltrationRateMeasure()
args_infiltration = measure_infiltration.arguments(model)
arg_map_infiltration = openstudio.measure.OSArgumentMap()
infiltration_ach_arg = args_infiltration[0].clone()
infiltration_ach_arg.setValue(infiltration_ach)
arg_map_infiltration["ach_value"] = infiltration_ach_arg
measures.append((measure_infiltration, arg_map_infiltration))
```

```
measure_boiler_flow = AdjustBoilerDesignWaterFlowRateMeasure()
args_boiler_flow = measure_boiler_flow.arguments(model)
arg_map_boiler_flow = openstudio.measure.OSArgumentMap()
design_water_flow_rate_arg = args_boiler_flow[0].clone()
design_water_flow_rate_arg.setValue(design_water_flow_rate)
arg_map_boiler_flow["design_water_flow_rate"] = design_water_flow_rate_arg
measures.append((measure_boiler_flow, arg_map_boiler_flow))

measure_boiler_min_plr = AdjustBoilerMinimumPartLoadRatioMeasure()
args_boiler_min_plr = measure_boiler_min_plr.arguments(model)
arg_map_boiler_min_plr = openstudio.measure.OSArgumentMap()
min_plr_arg = args_boiler_min_plr[0].clone()
min_plr_arg.setValue(minimum_part_load_ratio)
arg_map_boiler_min_plr["minimum_part_load_ratio"] = min_plr_arg
measures.append((measure_boiler_min_plr, arg_map_boiler_min_plr))

measure_boiler_max_plr = AdjustBoilerMaximumPartLoadRatioMeasure()
args_boiler_max_plr = measure_boiler_max_plr.arguments(model)
arg_map_boiler_max_plr = openstudio.measure.OSArgumentMap()
max_plr_arg = args_boiler_max_plr[0].clone()
max_plr_arg.setValue(maximum_part_load_ratio)
arg_map_boiler_max_plr["maximum_part_load_ratio"] = max_plr_arg
measures.append((measure_boiler_max_plr, arg_map_boiler_max_plr))

measure_coil_ua = AdjustHeatingCoilUFactorTimesAreaMeasure()
args_coil_ua = measure_coil_ua.arguments(model)
arg_map_coil_ua = openstudio.measure.OSArgumentMap()
ua_value_arg = args_coil_ua[0].clone()
ua_value_arg.setValue(ufactor_times_area_value)
arg_map_coil_ua["ufactor_times_area_value"] = ua_value_arg
measures.append((measure_coil_ua, arg_map_coil_ua))

measure_coil_max_flow = AdjustHeatingCoilMaximumWaterFlowRateMeasure()
args_coil_max_flow = measure_coil_max_flow.arguments(model)
arg_map_coil_max_flow = openstudio.measure.OSArgumentMap()
max_water_flow_rate_arg = args_coil_max_flow[0].clone()
max_water_flow_rate_arg.setValue(maximum_water_flow_rate)
arg_map_coil_max_flow["maximum_water_flow_rate"] = max_water_flow_rate_arg
measures.append((measure_coil_max_flow, arg_map_coil_max_flow))

for measure, arg_map in measures:
    measure.run(model, runner, arg_map)
    result = runner.result()
```

Fitness analysis

This step focuses on compiling all necessary attributes required for the model calibration process, specifically targeting the evaluation of simulation accuracy against actual measured data. The agent, Information Retriever, extracts relevant information from the user-provided inputs, such as the calibration target variable, the actual data file and corresponding column, the simulation data file and column, and the specified objective function for fitness analysis. All these attributes are then structured into a standardized JSON format, facilitating automated and transparent calibration workflows. This structured output ensures that the calibration procedure is reproducible and all essential information is explicitly defined for further analysis.

The dialogue with the Information Retriever agent for extracting calibration and fitness analysis attributes from user inputs:

Agent: Information Retriever (Consensus, gpt-4)

User Prompt:

I choose heating energy as the calibration target. The actual data is provided in the file dyson_data_2018.csv, where the relevant column name is actual heating energy. The simulation data is provided in the file eplusout.csv, where the relevant column name is HOT WATER BOILER:Boiler Heating Energy [J](Daily). I would like the objective function to be calculated using the Root Mean Square Error (RMSE) between the simulated values and the actual values.

System Prompt:

Extract the corresponding information from the user-provided information, including the calibration target, actual data file name, column name, objective function. Compile all the retrieved attributes into a JSON format file.

Example Response:

Please provide the results in a JSON format

Answer:


```json
{
    "calibration_target": "heating energy",
    "actual_data_file_name": "dyson_data_2018.csv",
    "actual_data_column_name": "actual heating energy",
    "simulation_data_file_name": "eplusout.csv",
    "simulation_data_column_name": "HOT WATER BOILER:Boiler Heating Energy [J](Daily)",
    "objective_function": "Root Mean Square Error (RMSE)"
}
```


The dialogue with Reviewer agent for evaluating whether the information extracted is accurate:

Agent: Reviewer (Consensus, gpt-4)

Files:

User Requirement of "Fitness analysis"

extracted_fitness_information.json

Answer:

- Entry: Calibration target is "heating energy"
- Confidence: 1

- Justification: The original information states "I choose heating energy as the calibration target."

- Entry: Actual data file name is "dyson_data_2018.csv"
- Confidence: 1
- Justification: The original information states "The actual data is provided in the file dyson_data_2018.csv."

- Entry: Actual data column name is "actual heating energy"
- Confidence: 1
- Justification: The original information states "the relevant column name is actual heating energy."

- Entry: Simulation data file name is "eplusout.csv"
- Confidence: 1
- Justification: The original information states "The simulation data is provided in the file eplusout.csv."

- Entry: Simulation data column name is "HOT WATER BOILER:Boiler Heating Energy J"
- Confidence: 1
- Justification: The original information states "the relevant column name is HOT WATER BOILER:Boiler Heating Energy J."

- Entry: Objective function is "Root Mean Square Error (RMSE)"
- Confidence: 1

Justification: The original information states "I would like the objective function to be calculated using the Root Mean Square Error (RMSE)."

Fitness calculation

This step focuses on evaluating the accuracy of simulation results by comparing them against actual measured data, using a fitness or error calculation method. The agent responsible for this task is the Programmer. Provided with calibration parameter data in JSON format, the agent systematically updates code placeholders with the real data file and column names, ensuring the workflow is tailored to the specific dataset. The process includes loading both measured and simulated data, preprocessing the datasets, aligning their lengths, and computing the objective function—typically a statistical error metric such as root mean square error (RMSE)—to quantify the agreement between the simulation and real-world observations. This calculated error serves as a key metric for model calibration and validation in subsequent analyses.

Dialogue with Programmer agent for calculating fitness using calibration data and simulation outputs:

Agent: Programmer (Consensus, gpt-4)

Files:

extracted_calibration_parameter_content.json

System Prompt:

Using the calibration data provided in the JSON file, replace the placeholders in the example response with the corresponding real names. For the objective function, you should write the calculation equation.

Example Response:

READ actual data from 'CalibrationDataFile.csv', with dates parsed and first column as index

EXTRACT the actual data series using 'CalibrationDataColumnName'

SET the simulation data path by joining the simulation output directory, 'run', and 'CalibrationSimulationFile.csv'

IF the simulation data file does not exist:
    PRINT a message indicating the file is missing and simulation should be checked
    RETURN infinity as error

TRY to read simulation data from the simulation data path
IF there is an error while reading:
    PRINT the error message
    RETURN infinity as error

REMOVE rows with missing values from simulation data
SET the variable column name for simulation data as 'CalibrationSimulationVariableName'

IF the variable column name exists in simulation data columns:
    CREATE a 'simulated' column by dividing the selected simulation variable by a scaling factor
ELSE:
    PRINT a message that the required simulation column is missing
    PRINT the available columns in the simulation output
    RETURN infinity as error

COPY the actual data to a new object
CALCULATE the minimum length between simulation data and actual data
TRIM both data series to the minimum length
ASSIGN the 'simulated' values from simulation data to the copied actual data

CALCULATE error as the sum of the squared differences between actual and simulated data

PRINT the evaluation completion message with the error value

SET the path for saving the processed simulation results as 'final_simulated.csv' in the simulation output directory
SAVE the combined actual and simulated data to CSV
PRINT a message confirming results have been saved

Answer:

```
# Load actual data
actual_data = pd.read_csv('dyson_data_2018.csv', parse_dates=True, index_col=0)
actual_data_series = actual_data['actual heating energy']

# Define simulation data path
simulation_data_path = os.path.join(sim_output_dir, 'run', 'eplusout.csv')
```

```
# Check if simulation data file exists
if not os.path.exists(simulation_data_path):
    print(f"File {simulation_data_path} does not exist. Please ensure the simulation completed successfully and the file was generated.")
    return (float('inf'),)

# Try to load simulation data
try:
    simulation_data = pd.read_csv(simulation_data_path)
except Exception as e:
    print(f"An error occurred while reading the CSV file: {e}")
    return (float('inf'),)

# Preprocess simulation data
simulation_data.dropna(how='any', inplace=True)
simulation_variable_column_name = 'HOT WATER BOILER:Boiler Heating Energy [J](Daily)'
if simulation_variable_column_name in simulation_data.columns:
    simulation_data['simulated'] = simulation_data[simulation_variable_column_name] / 3600000
else:
    print(f"The column '{simulation_variable_column_name}' does not exist in eplusout.csv.")
    print("Available columns in eplusout.csv:", simulation_data.columns.tolist())
    return (float('inf'),)

# Prepare data for error calculation
data_copy = actual_data.copy()
min_length = min(len(simulation_data), len(data_copy))
data_copy = data_copy.iloc[:min_length]
simulation_data = simulation_data.iloc[:min_length]
data_copy['simulated'] = simulation_data['simulated'].values

# Calculate error
error = np.sqrt(((data_copy['simulated'] - data_copy['actual heating energy']) ** 2).mean())
print(f"\nEvaluation completed, error: {error}")

# Save final simulated data
modified_csv_path = os.path.join(sim_output_dir, 'final_simulated.csv')
data_copy.to_csv(modified_csv_path, index=True)
print(f"Data processing complete. Results saved to {modified_csv_path}")
```

Optimization execution

In this section, the agent configures population evaluation, conducts the evolution process, and records the best offspring through systematic parameter adjustments. The process culminates in the integration and execution

of calibration code, optimizing the model based on real-world data and improving the accuracy of simulation results for energy consumption and system performance.

Population evaluation

This step focuses on initializing and evaluating the population within the evolutionary algorithm, based on user-defined parameters. The Programmer agent is responsible for configuring key settings, including population size, number of generations, crossover probability, and mutation probability, to align with user requirements. After setting these parameters, the agent generates an initial population, calculates fitness values for each individual, and records the best fitness score in each generation.

The dialogue with Programmer agent for setting and evaluating population parameters in the evolutionary algorithm:

Agent: Programmer (Consensus, gpt-4)

User Prompt:

I need to create an evolutionary algorithm with the following parameters: the population size should be 100, the number of generations should be 20, the crossover probability should be set to 0.5, and the mutation probability should be 0.2.

System Prompt:

Set up evolutionary algorithm parameters, to align with user requirements.

Example Response:

population = toolbox.population(n=ExampleValue)

NGEN = ExampleValue

CXPB = ExampleValue

MUTPB = ExampleValue

Answer:

```
population = toolbox.population(n=100)
NGEN = 20
CXPB = 0.5
MUTPB = 0.2

best_fitness_values = []
fitnesses = list(map(toolbox.evaluate, population))
for ind, fit in zip(population, fitnesses):
    ind.fitness.values = fit
fits = [ind.fitness.values[0] for ind in population if np.isfinite(ind.fitness.values[0])]
if fits:
    best_fitness = min(fits)
    best_fitness_values.append(best_fitness)
else:
    best_fitness_values.append(float('inf'))
```

Evolution process conduction

This step details the execution of the evolutionary optimization process for model parameter calibration. The Programmer agent is responsible for implementing the evolution logic, utilizing calibration parameter ranges extracted from the Code for Parameter range assignment.py file. The final code ensures that, during crossover and mutation, each individual's gene is constrained within its designated parameter range, thereby maintaining valid candidate solutions throughout the optimization process.

Dialogue with Programmer agent for conducting the evolution process and applying parameter range constraints in the evolutionary algorithm:

Agent: Programmer (Consensus, gpt-4)

Files:

Code for Parameter range assignment.py

System Prompt:

According to the calibration parameter range names from the Code for Parameter range assignment.py file, replace the placeholders in the example response with the corresponding range names.

Example Response:

```
FOR each pair of children (child1, child2) in the offspring:
    IF a random condition for crossover is met:
        APPLY the crossover operation to child1 and child2
        SET the first parameter of child1 and child2 within the bounds of CalibrationRangeName1
        SET the second parameter of child1 and child2 within the bounds of CalibrationRangeName2
        SET the third parameter of child1 and child2 within the bounds of CalibrationRangeName3
        ...
        DELETE the fitness values of child1 and child2

FOR each mutant individual in the offspring:
    IF a random condition for mutation is met:
        APPLY the mutation operation to the mutant
        SET the first parameter of the mutant within the bounds of CalibrationRangeName1
        SET the second parameter of the mutant within the bounds of CalibrationRangeName2
        SET the third parameter of the mutant within the bounds of CalibrationRangeName3
        ...
        DELETE the fitness values of the mutant
```

Answer:

```
for gen in range(NGEN):
        print(f"Starting generation {gen + 1} of evolution")
        offspring = toolbox.select(population, len(population))
        offspring = list(map(toolbox.clone, offspring))

for child1, child2 in zip(offspring[::2], offspring[1::2]):
    if np.random.rand() < CXPB:
        toolbox.mate(child1, child2)
        for i in range(len(child1)):
```

```
            if i == 0:
                child1[i] = np.clip(child1[i], *heating_setpoint_range)
                child2[i] = np.clip(child2[i], *heating_setpoint_range)
            elif i == 1:
                child1[i] = np.clip(child1[i], *equipment_power_density_range)
                child2[i] = np.clip(child2[i], *equipment_power_density_range)
            elif i == 2:
                child1[i] = np.clip(child1[i], *lighting_power_density_range)
                child2[i] = np.clip(child2[i], *lighting_power_density_range)
            elif i == 3:
                child1[i] = np.clip(child1[i], *number_of_people_accommodated_range)
                child2[i] = np.clip(child2[i], *number_of_people_accommodated_range)
            elif i == 4:
                child1[i] = np.clip(child1[i], *infiltration_air_changes_per_hour_range)
                child2[i] = np.clip(child2[i], *infiltration_air_changes_per_hour_range)
            elif i == 5:
                child1[i] = np.clip(child1[i], *boiler_design_water_flow_rate_range)
                child2[i] = np.clip(child2[i], *boiler_design_water_flow_rate_range)
            elif i == 6:
                child1[i] = np.clip(child1[i], *boiler_minimum_part_load_ratio_range)
                child2[i] = np.clip(child2[i], *boiler_minimum_part_load_ratio_range)
            elif i == 7:
                child1[i] = np.clip(child1[i], *boiler_maximum_part_load_ratio_range)
                child2[i] = np.clip(child2[i], *boiler_maximum_part_load_ratio_range)
            elif i == 8:
                child1[i] = np.clip(child1[i], *heating_coil_U_factor_multiplied_by_area_value_range)
                child2[i] = np.clip(child2[i], *heating_coil_U_factor_multiplied_by_area_value_range)
            elif i == 9:
                child1[i] = np.clip(child1[i], *heating_coil_maximum_water_flow_rate_range)
                child2[i] = np.clip(child2[i], *heating_coil_maximum_water_flow_rate_range)
        del child1.fitness.values
        del child2.fitness.values

for mutant in offspring:
    if np.random.rand() < MUTPB:
        toolbox.mutate(mutant)
        for i in range(len(mutant)):
            if i == 0:
                mutant[i] = np.clip(mutant[i], *heating_setpoint_range)
            elif i == 1:
                mutant[i] = np.clip(mutant[i], *equipment_power_density_range)
            elif i == 2:
                mutant[i] = np.clip(mutant[i], *lighting_power_density_range)
            elif i == 3:
```

```
                mutant[i] = np.clip(mutant[i], *number_of_people_accommodated_range)
            elif i == 4:
                mutant[i] = np.clip(mutant[i], *infiltration_air_changes_per_hour_range)
            elif i == 5:
                mutant[i] = np.clip(mutant[i], *boiler_design_water_flow_rate_range)
            elif i == 6:
                mutant[i] = np.clip(mutant[i], *boiler_minimum_part_load_ratio_range)
            elif i == 7:
                mutant[i] = np.clip(mutant[i], *boiler_maximum_part_load_ratio_range)
            elif i == 8:
                mutant[i] = np.clip(mutant[i], *heating_coil_U_factor_multiplied_by_area_value_range)
            elif i == 9:
                mutant[i] = np.clip(mutant[i], *heating_coil_maximum_water_flow_rate_range)
        del mutant.fitness.values

invalid_ind = [ind for ind in offspring if not ind.fitness.valid]
fitnesses = map(toolbox.evaluate, invalid_ind)
for ind, fit in zip(invalid_ind, fitnesses):
    ind.fitness.values = fit

population[:] = offspring
fits = [ind.fitness.values[0] for ind in population if np.isfinite(ind.fitness.values[0])]

if fits:
    best_fitness = min(fits)
    best_fitness_values.append(best_fitness)
else:
    best_fitness_values.append(float('inf'))
    print("All individuals have invalid fitness values (inf).")
```

Best offspring record

This step focuses on programmatically recording the optimal set of calibration parameters, referred to as the "best offspring", identified during the model calibration process. The Programmer agent receives a JSON file containing the extracted calibration parameter information. The agent is instructed to systematically substitute placeholder variable names in the provided example code with the actual parameter names found in the JSON, ensuring no structural changes or unnecessary code modifications are introduced. This automated replacement generates Python code that explicitly records each calibration parameter, assembles a unique identifier string, and saves the best parameter set into a designated output file.

Dialogue with the Programmer agent for generating code to record the best offspring parameter set based on extracted calibration parameters:

Agent: Programmer (Consensus, gpt-4)

Files:

extracted_calibration_parameter_information.json

System Prompt:

Using the calibration parameter data provided in the JSON file, replace the placeholders in the example response with the corresponding real names.

Example Response:

best_ind = select the best individual from the population

calibration_parameter_1 = best_ind[0]
calibration_parameter_2 = best_ind[1]
calibration_parameter_3 = best_ind[2]
...
calibration_parameter_N = best_ind[N]

best_param_str = (
    f"calibration_parameter_1_" with the formatted value
    f"calibration_parameter_2_" with the formatted value
    f"calibration_parameter_3_" with the formatted value
    ...
    f"calibration_parameter_N_" with the formatted value
)

best_params_file = join the output directory path with 'best_parameters.txt'
with open(best_params_file, 'w') as file_handle:
    write "Best Parameter Set:\n" to the file
    write the formatted value of calibration_parameter_1 to the file
    write the formatted value of calibration_parameter_2 to the file
    write the formatted value of calibration_parameter_3 to the file
    ...
    write the formatted value of calibration_parameter_N to the file

Answer:

```
best_ind = tools.selBest(population, 1)[0]
heating_setpoint = best_ind[0]
equipment_power_density = best_ind[1]
lighting_power_density = best_ind[2]
number_of_people_accommodated = best_ind[3]
infiltration_air_changes_per_hour = best_ind[4]
boiler_design_water_flow_rate = best_ind[5]
boiler_minimum_part_load_ratio = best_ind[6]
boiler_maximum_part_load_ratio = best_ind[7]
heating_coil_U_factor_multiplied_by_area_value = best_ind[8]
heating_coil_maximum_water_flow_rate = best_ind[9]

best_param_str = (
```

```
    f"hsp_{round(heating_setpoint, 2)}_"
    f"epd_{round(equipment_power_density, 2)}_"
    f"lpd_{round(lighting_power_density, 2)}_"
    f"npa_{round(number_of_people_accommodated, 2)}_"
    f"iach_{round(infiltration_air_changes_per_hour, 2)}_"
    f"bdwfr_{round(boiler_design_water_flow_rate, 2)}_"
    f"bmp_lr_{round(boiler_minimum_part_load_ratio, 2)}_"
    f"bmp_lr_{round(boiler_maximum_part_load_ratio, 2)}_"
    f"hcu_factor_{round(heating_coil_U_factor_multiplied_by_area_value, 2)}_"
    f"hcmwfr_{round(heating_coil_maximum_water_flow_rate, 2)}"
)

best_params_file = os.path.join(output_directory, 'best_parameters.txt')
with open(best_params_file, 'w') as f:
    f.write("Best Parameter Set:\n")
    f.write(f"    heating setpoint = {round(heating_setpoint, 2)} °C\n")
    f.write(f"    equipment power density = {round(equipment_power_density, 2)} W/m²\n")
    f.write(f"    lighting power density = {round(lighting_power_density, 2)} W/m²\n")
    f.write(f"    number of people accommodated = {round(number_of_people_accommodated, 2)}\n")
    f.write(f"    infiltration air changes per hour = {round(infiltration_air_changes_per_hour, 2)} ACH\n")
    f.write(f"    boiler design water flow rate = {round(boiler_design_water_flow_rate, 2)} m³/s\n")
    f.write(f"    boiler minimum part load ratio = {round(boiler_minimum_part_load_ratio, 2)}\n")
    f.write(f"    boiler maximum part load ratio = {round(boiler_maximum_part_load_ratio, 2)}\n")
    f.write(f"    heating coil U-factor multiplied by area value = 
{round(heating_coil_U_factor_multiplied_by_area_value, 2)} W/K\n")
    f.write(f"    heating coil maximum water flow rate = {round(heating_coil_maximum_water_flow_rate, 2)} 
m³/s\n")

sim_output_dir = parameter_output_dirs.get(best_param_str)
```

Calibration code integration

In this section, the codes developed are integrated and assembled into a complete, executable calibration script. This consolidated code brings together all essential parameter identification, data handling, and calibration routines required for the model. The resulting script can be run directly to perform the calibration process on the OpenStudio model, ensuring that all model parameters are efficiently tuned based on the provided data.

```
import os
import subprocess
import json
import pandas as pd
import numpy as np
from deap import base, creator, tools
import openstudio
import uuid
from datetime import datetime
```

```python
import pathlib
import importlib.util
import copy
import matplotlib.pyplot as plt
from sklearn.model_selection import train_test_split

# Read model path and timestamp from file
with open('model_info.txt', 'r') as info_file:
    lines = info_file.readlines()
    save_path = lines[0].strip()
    model_time = lines[1].strip()

# Convert model path to absolute path
save_path = os.path.abspath(save_path)
print(f"Model file path: {save_path}")

# Get the current timestamp
current_time = datetime.now().strftime('%Y%m%d_%H%M%S')

# Set output directory, including model timestamp and current timestamp
output_directory = f'output_{model_time}_{current_time}'

# Create output directory (if not exists)
if not os.path.exists(output_directory):
    os.makedirs(output_directory)

# Define OpenStudio CLI path
openstudio_cli_path = "/usr/local/bin/openstudio"  # Adjust based on actual setup

# Load actual heating energy consumption data
actual_energy_data = pd.read_csv('dyson-gas-2018-process-1d-correct.csv', parse_dates=True, index_col=0)
actual_energy_series = actual_energy_data['actual heating energy']  # Adjust column name

train_data, temp_data = train_test_split(
    actual_energy_data,
    test_size=0.2,
    random_state=42,
    shuffle=True
)
val_data, test_data = train_test_split(
    temp_data,
    test_size=0.5,
    random_state=42,
    shuffle=True
```

```
)

# Define parameter ranges
heating_setpoint_range = (18, 24)
equipment_power_density_range = (3, 22)
lighting_power_density_range = (1.5, 15)
number_of_people_range = (30, 200)   # Adjust based on building area and office scenario
infiltration_ach_range = (0.5, 1.5)   # Adjust based on actual conditions

# Additional parameter ranges
design_water_flow_rate_range = (0.0001, 0.005)   # m³/s
minimum_part_load_ratio_range = (0.1, 0.4)
maximum_part_load_ratio_range = (0.6, 0.9)
ufactor_times_area_value_range = (500.0, 2000.0)   # W/K
maximum_water_flow_rate_range = (0.001, 0.006)   # m³/s

# Create a global dictionary to map parameter combinations to simulation output directories
parameter_output_dirs = {}

# Import all Measures

# AdjustHeatingSetpointMeasure
measure_name_hsp = "AdjustHeatingSetpointMeasure"
measure_path_hsp = pathlib.Path(__file__).parent / "measures" / measure_name_hsp / "measure.py"
spec_hsp = importlib.util.spec_from_file_location(measure_name_hsp, measure_path_hsp)
measure_module_hsp = importlib.util.module_from_spec(spec_hsp)
spec_hsp.loader.exec_module(measure_module_hsp)
AdjustHeatingSetpointMeasure = measure_module_hsp.AdjustHeatingSetpointMeasure

# AdjustEquipmentPowerDensityMeasure
measure_name_epd = "AdjustEquipmentPowerDensityMeasure"
measure_path_epd = pathlib.Path(__file__).parent / "measures" / measure_name_epd / "measure.py"
spec_epd = importlib.util.spec_from_file_location(measure_name_epd, measure_path_epd)
measure_module_epd = importlib.util.module_from_spec(spec_epd)
spec_epd.loader.exec_module(measure_module_epd)
AdjustEquipmentPowerDensityMeasure = measure_module_epd.AdjustEquipmentPowerDensityMeasure

# AdjustLightingPowerDensityMeasure
measure_name_lpd = "AdjustLightingPowerDensityMeasure"
measure_path_lpd = pathlib.Path(__file__).parent / "measures" / measure_name_lpd / "measure.py"
spec_lpd = importlib.util.spec_from_file_location(measure_name_lpd, measure_path_lpd)
measure_module_lpd = importlib.util.module_from_spec(spec_lpd)
spec_lpd.loader.exec_module(measure_module_lpd)
AdjustLightingPowerDensityMeasure = measure_module_lpd.AdjustLightingPowerDensityMeasure
```

```
# AdjustPeopleNumberMeasure
measure_name_pn = "AdjustPeopleNumberMeasure"
measure_path_pn = pathlib.Path(__file__).parent / "measures" / measure_name_pn / "measure.py"
spec_pn = importlib.util.spec_from_file_location(measure_name_pn, measure_path_pn)
measure_module_pn = importlib.util.module_from_spec(spec_pn)
spec_pn.loader.exec_module(measure_module_pn)
AdjustPeopleNumberMeasure = measure_module_pn.AdjustPeopleNumberMeasure

# AdjustInfiltrationRateMeasure
measure_name_infiltration = "AdjustInfiltrationRateMeasure"
measure_path_infiltration = pathlib.Path(__file__).parent / "measures" / measure_name_infiltration /
"measure.py"
spec_infiltration = importlib.util.spec_from_file_location(measure_name_infiltration, measure_path_infiltration)
measure_module_infiltration = importlib.util.module_from_spec(spec_infiltration)
spec_infiltration.loader.exec_module(measure_module_infiltration)
AdjustInfiltrationRateMeasure = measure_module_infiltration.AdjustInfiltrationRateMeasure

# Import additional Measures

# AdjustBoilerDesignWaterFlowRateMeasure
measure_name_boiler_flow = "AdjustBoilerDesignWaterFlowRateMeasure"
measure_path_boiler_flow = pathlib.Path(__file__).parent / "measures" / measure_name_boiler_flow /
"measure.py"
spec_boiler_flow = importlib.util.spec_from_file_location(measure_name_boiler_flow,
measure_path_boiler_flow)
measure_module_boiler_flow = importlib.util.module_from_spec(spec_boiler_flow)
spec_boiler_flow.loader.exec_module(measure_module_boiler_flow)
AdjustBoilerDesignWaterFlowRateMeasure =
measure_module_boiler_flow.AdjustBoilerDesignWaterFlowRateMeasure

# AdjustBoilerMinimumPartLoadRatioMeasure
measure_name_boiler_min_plr = "AdjustBoilerMinimumPartLoadRatioMeasure"
measure_path_boiler_min_plr = pathlib.Path(__file__).parent / "measures" / measure_name_boiler_min_plr /
"measure.py"
spec_boiler_min_plr = importlib.util.spec_from_file_location(measure_name_boiler_min_plr,
measure_path_boiler_min_plr)
measure_module_boiler_min_plr = importlib.util.module_from_spec(spec_boiler_min_plr)
spec_boiler_min_plr.loader.exec_module(measure_module_boiler_min_plr)
AdjustBoilerMinimumPartLoadRatioMeasure =
measure_module_boiler_min_plr.AdjustBoilerMinimumPartLoadRatioMeasure

# AdjustBoilerMaximumPartLoadRatioMeasure
measure_name_boiler_max_plr = "AdjustBoilerMaximumPartLoadRatioMeasure"
```

```
measure_path_boiler_max_plr = pathlib.Path(__file__).parent / "measures" / measure_name_boiler_max_plr /
"measure.py"
spec_boiler_max_plr = importlib.util.spec_from_file_location(measure_name_boiler_max_plr,
measure_path_boiler_max_plr)
measure_module_boiler_max_plr = importlib.util.module_from_spec(spec_boiler_max_plr)
spec_boiler_max_plr.loader.exec_module(measure_module_boiler_max_plr)
AdjustBoilerMaximumPartLoadRatioMeasure =
measure_module_boiler_max_plr.AdjustBoilerMaximumPartLoadRatioMeasure

# AdjustHeatingCoilUFactorTimesAreaMeasure
measure_name_coil_ua = "AdjustHeatingCoilUFactorTimesAreaMeasure"
measure_path_coil_ua = pathlib.Path(__file__).parent / "measures" / measure_name_coil_ua / "measure.py"
spec_coil_ua = importlib.util.spec_from_file_location(measure_name_coil_ua, measure_path_coil_ua)
measure_module_coil_ua = importlib.util.module_from_spec(spec_coil_ua)
spec_coil_ua.loader.exec_module(measure_module_coil_ua)
AdjustHeatingCoilUFactorTimesAreaMeasure =
measure_module_coil_ua.AdjustHeatingCoilUFactorTimesAreaMeasure

# AdjustHeatingCoilMaximumWaterFlowRateMeasure
measure_name_coil_max_flow = "AdjustHeatingCoilMaximumWaterFlowRateMeasure"
measure_path_coil_max_flow = pathlib.Path(__file__).parent / "measures" / measure_name_coil_max_flow /
"measure.py"
spec_coil_max_flow = importlib.util.spec_from_file_location(measure_name_coil_max_flow,
measure_path_coil_max_flow)
measure_module_coil_max_flow = importlib.util.module_from_spec(spec_coil_max_flow)
spec_coil_max_flow.loader.exec_module(measure_module_coil_max_flow)
AdjustHeatingCoilMaximumWaterFlowRateMeasure =
measure_module_coil_max_flow.AdjustHeatingCoilMaximumWaterFlowRateMeasure

# Register custom clone function
toolbox = base.Toolbox()
toolbox.register("clone", copy.deepcopy)

# Set genetic algorithm parameters
if not hasattr(creator, "FitnessMin"):
    creator.create("FitnessMin", base.Fitness, weights=(-1.0,))  # Minimize objective function
if not hasattr(creator, "Individual"):
    creator.create("Individual", list, fitness=creator.FitnessMin)

# Register attributes
toolbox.register("attr_heating_setpoint", np.random.uniform, *heating_setpoint_range)
toolbox.register("attr_equipment_power_density", np.random.uniform, *equipment_power_density_range)
toolbox.register("attr_lighting_power_density", np.random.uniform, *lighting_power_density_range)
toolbox.register("attr_number_of_people", np.random.uniform, *number_of_people_range)
```

```
toolbox.register("attr_infiltration_ach", np.random.uniform, *infiltration_ach_range)

# Register additional attributes
toolbox.register("attr_design_water_flow_rate", np.random.uniform, *design_water_flow_rate_range)
toolbox.register("attr_minimum_part_load_ratio", np.random.uniform, *minimum_part_load_ratio_range)
toolbox.register("attr_maximum_part_load_ratio", np.random.uniform, *maximum_part_load_ratio_range)
toolbox.register("attr_ufactor_times_area_value", np.random.uniform, *ufactor_times_area_value_range)
toolbox.register("attr_maximum_water_flow_rate", np.random.uniform, *maximum_water_flow_rate_range)

# Create an individual using the specified attributes
toolbox.register("individual", tools.initCycle, creator.Individual,
                 (toolbox.attr_heating_setpoint,
                  toolbox.attr_equipment_power_density,
                  toolbox.attr_lighting_power_density,
                  toolbox.attr_number_of_people,
                  toolbox.attr_infiltration_ach,
                  toolbox.attr_design_water_flow_rate,
                  toolbox.attr_minimum_part_load_ratio,
                  toolbox.attr_maximum_part_load_ratio,
                  toolbox.attr_ufactor_times_area_value,
                  toolbox.attr_maximum_water_flow_rate
                ), n=1)

# Create a population
toolbox.register("population", tools.initRepeat, list, toolbox.individual)

toolbox.register("evaluate", lambda ind: evaluate(ind))
toolbox.register("mate", tools.cxBlend, alpha=0.5)
toolbox.register("mutate", tools.mutGaussian, mu=0, sigma=1, indpb=1.0)
toolbox.register("select", tools.selTournament, tournsize=3)

# Define the evaluation function
def evaluate(individual):
    # Extract individual parameters
    heating_setpoint = individual[0]
    equipment_power_density = individual[1]
    lighting_power_density = individual[2]
    number_of_people = individual[3]
    infiltration_ach = individual[4]
    design_water_flow_rate = individual[5]
    minimum_part_load_ratio = individual[6]
    maximum_part_load_ratio = individual[7]
    ufactor_times_area_value = individual[8]
    maximum_water_flow_rate = individual[9]
```

```
    # Round the number of people to the nearest integer
    number_of_people_int = int(round(number_of_people))

    # Create a unique output directory with a simplified name
    sim_output_dir = os.path.join(output_directory, f"sim_{uuid.uuid4().hex}")
    if not os.path.exists(sim_output_dir):
        os.makedirs(sim_output_dir)

    # Store parameter combinations and output directory in a global dictionary
    param_str = f"hsp_{round(heating_setpoint, 2)}_epd_{round(equipment_power_density,
2)}_lpd_{round(lighting_power_density, 2)}_pn_{number_of_people_int}_ach_{round(infiltration_ach,
2)}_flow_{round(design_water_flow_rate, 4)}_minplr_{round(minimum_part_load_ratio,
2)}_maxplr_{round(maximum_part_load_ratio, 2)}_ua_{round(ufactor_times_area_value,
2)}_maxflow_{round(maximum_water_flow_rate, 4)}"
    parameter_output_dirs[param_str] = sim_output_dir

    # Print information about the evaluated individual
    print("\n" + "-"*50)
    print(f"Evaluating individual:")
    print(f"   Heating setpoint = {round(heating_setpoint, 2)} °C")
    print(f"   Equipment power density = {round(equipment_power_density, 2)} W/m²")
    print(f"   Lighting power density = {round(lighting_power_density, 2)} W/m²")
    print(f"   Number of people = {number_of_people_int} persons")
    print(f"   Infiltration rate = {round(infiltration_ach, 2)} ACH")
    print(f"   Boiler design water flow rate = {round(design_water_flow_rate, 4)} m³/s")
    print(f"   Boiler minimum part load ratio = {round(minimum_part_load_ratio, 2)}")
    print(f"   Boiler maximum part load ratio = {round(maximum_part_load_ratio, 2)}")
    print(f"   Heating coil U-factor times area = {round(ufactor_times_area_value, 2)} W/K")
    print(f"   Heating coil maximum water flow rate = {round(maximum_water_flow_rate, 4)} m³/s")
    print("-"*50)

    # Check if the model file exists
    if not os.path.exists(save_path):
        print(f"Model file does not exist: {save_path}")
        return (float('inf'),)

    # Load the model
    try:
        translator = openstudio.osversion.VersionTranslator()
        model_path = openstudio.path(save_path)
        model = translator.loadModel(model_path)
        if model.is_initialized():
            model = model.get()
```

```
        else:
            print("Failed to load the model. It might be corrupted or incompatible.")
            return (float('inf'),)
    except Exception as e:
        print(f"An exception occurred while loading the model: {e}")
        return (float('inf'),)

    # Create a runner
    runner = openstudio.measure.OSRunner(openstudio.WorkflowJSON())

    # Create Measure instances and argument mappings
    measures = []

    # AdjustPeopleNumberMeasure
    measure_pn = AdjustPeopleNumberMeasure()
    args_pn = measure_pn.arguments(model)
    arg_map_pn = openstudio.measure.OSArgumentMap()
    number_of_people_arg = args_pn[0].clone()
    number_of_people_arg.setValue(number_of_people_int)
    arg_map_pn["number_of_people"] = number_of_people_arg
    measures.append((measure_pn, arg_map_pn))

    # AdjustEquipmentPowerDensityMeasure
    measure_epd = AdjustEquipmentPowerDensityMeasure()
    args_epd = measure_epd.arguments(model)
    arg_map_epd = openstudio.measure.OSArgumentMap()
    epd_arg = args_epd[0].clone()
    epd_arg.setValue(equipment_power_density)
    arg_map_epd["epd_value"] = epd_arg
    measures.append((measure_epd, arg_map_epd))

    # AdjustLightingPowerDensityMeasure
    measure_lpd = AdjustLightingPowerDensityMeasure()
    args_lpd = measure_lpd.arguments(model)
    arg_map_lpd = openstudio.measure.OSArgumentMap()
    lpd_arg = args_lpd[0].clone()
    lpd_arg.setValue(lighting_power_density)
    arg_map_lpd["lpd_value"] = lpd_arg
    measures.append((measure_lpd, arg_map_lpd))

    # AdjustHeatingSetpointMeasure
    measure_hsp = AdjustHeatingSetpointMeasure()
    args_hsp = measure_hsp.arguments(model)
    arg_map_hsp = openstudio.measure.OSArgumentMap()
```

```python
hsp_arg = args_hsp[0].clone()
hsp_arg.setValue(heating_setpoint)
arg_map_hsp["heating_setpoint"] = hsp_arg
measures.append((measure_hsp, arg_map_hsp))

# AdjustInfiltrationRateMeasure
measure_infiltration = AdjustInfiltrationRateMeasure()
args_infiltration = measure_infiltration.arguments(model)
arg_map_infiltration = openstudio.measure.OSArgumentMap()
infiltration_ach_arg = args_infiltration[0].clone()
infiltration_ach_arg.setValue(infiltration_ach)
arg_map_infiltration["ach_value"] = infiltration_ach_arg
measures.append((measure_infiltration, arg_map_infiltration))

# AdjustBoilerDesignWaterFlowRateMeasure
measure_boiler_flow = AdjustBoilerDesignWaterFlowRateMeasure()
args_boiler_flow = measure_boiler_flow.arguments(model)
arg_map_boiler_flow = openstudio.measure.OSArgumentMap()
design_water_flow_rate_arg = args_boiler_flow[0].clone()
design_water_flow_rate_arg.setValue(design_water_flow_rate)
arg_map_boiler_flow["design_water_flow_rate"] = design_water_flow_rate_arg
measures.append((measure_boiler_flow, arg_map_boiler_flow))

# AdjustBoilerMinimumPartLoadRatioMeasure
measure_boiler_min_plr = AdjustBoilerMinimumPartLoadRatioMeasure()
args_boiler_min_plr = measure_boiler_min_plr.arguments(model)
arg_map_boiler_min_plr = openstudio.measure.OSArgumentMap()
min_plr_arg = args_boiler_min_plr[0].clone()
min_plr_arg.setValue(minimum_part_load_ratio)
arg_map_boiler_min_plr["minimum_part_load_ratio"] = min_plr_arg
measures.append((measure_boiler_min_plr, arg_map_boiler_min_plr))

# AdjustBoilerMaximumPartLoadRatioMeasure
measure_boiler_max_plr = AdjustBoilerMaximumPartLoadRatioMeasure()
args_boiler_max_plr = measure_boiler_max_plr.arguments(model)
arg_map_boiler_max_plr = openstudio.measure.OSArgumentMap()
max_plr_arg = args_boiler_max_plr[0].clone()
max_plr_arg.setValue(maximum_part_load_ratio)
arg_map_boiler_max_plr["maximum_part_load_ratio"] = max_plr_arg
measures.append((measure_boiler_max_plr, arg_map_boiler_max_plr))

# AdjustHeatingCoilUFactorTimesAreaMeasure
measure_coil_ua = AdjustHeatingCoilUFactorTimesAreaMeasure()
args_coil_ua = measure_coil_ua.arguments(model)
```

```python
arg_map_coil_ua = openstudio.measure.OSArgumentMap()
ua_value_arg = args_coil_ua[0].clone()
ua_value_arg.setValue(ufactor_times_area_value)
arg_map_coil_ua["ufactor_times_area_value"] = ua_value_arg
measures.append((measure_coil_ua, arg_map_coil_ua))

# AdjustHeatingCoilMaximumWaterFlowRateMeasure
measure_coil_max_flow = AdjustHeatingCoilMaximumWaterFlowRateMeasure()
args_coil_max_flow = measure_coil_max_flow.arguments(model)
arg_map_coil_max_flow = openstudio.measure.OSArgumentMap()
max_water_flow_rate_arg = args_coil_max_flow[0].clone()
max_water_flow_rate_arg.setValue(maximum_water_flow_rate)
arg_map_coil_max_flow["maximum_water_flow_rate"] = max_water_flow_rate_arg
measures.append((measure_coil_max_flow, arg_map_coil_max_flow))

# Apply Measures sequentially
for measure, arg_map in measures:
    measure.run(model, runner, arg_map)
    result = runner.result()

    # Check if Measure executed successfully
    if result.value().valueName() != 'Success':
        print("Measure execution failed. Stopping simulation.")
        return (float('inf'),)

# Save the modified model to sim_output_dir
modified_model_path = os.path.join(sim_output_dir, 'modified_model.osm')
model.save(modified_model_path, True)

# Create the .osw file with relative paths and omit the run_directory
osw_content = {
    "seed_file": "modified_model.osm",   # Use relative path
    "measure_paths": [os.path.abspath('measures')],
    "steps": []
}

osw_path = os.path.join(sim_output_dir, 'in.osw')
with open(osw_path, 'w') as osw_file:
    json.dump(osw_content, osw_file)

# Run simulation using OpenStudio CLI
command = [
    openstudio_cli_path,
    "run",
```

```
        "-w",   # Use workflow file
        osw_path
    ]

    print(f"\nStarting simulation with parameter set: {param_str}")

    # Execute the command
    result = subprocess.run(command, capture_output=True, text=True)

    # Extract boiler energy consumption data from simulation
    csv_output_path = os.path.join(sim_output_dir, 'run', 'eplusout.csv')

    # Check if eplusout.csv exists
    if not os.path.exists(csv_output_path):
        print(f"File {csv_output_path} does not exist. Please ensure the simulation completed successfully
and generated this file.")
        return (float('inf'),)

    # Read eplusout.csv
    try:
        simulation_data = pd.read_csv(csv_output_path)
    except Exception as e:
        print(f"Error reading the CSV file: {e}")
        return (float('inf'),)

    # Drop rows with missing values
    simulation_data.dropna(how='any', inplace=True)

    # Extract the target column and perform unit conversion
    variable_name = 'HOT WATER BOILER:Boiler Heating Energy [J](Daily)'   # Adjust based on actual data
    if variable_name in simulation_data.columns:
        simulation_data['simulated'] = simulation_data[variable_name] / 3600000   # Convert to kWh
    else:
        print(f"The column '{variable_name}' does not exist in eplusout.csv.")
        print("Columns in eplusout.csv:", simulation_data.columns.tolist())
        return (float('inf'),)

    # Use global actual_energy_data
    dyson_df = test_data.copy()

    # Ensure data lengths are consistent
    min_length = min(len(simulation_data), len(dyson_df))
    dyson_df = dyson_df.iloc[:min_length]
    simulation_data = simulation_data.iloc[:min_length]
```

```
    # Add simulation data as the 'simulated' column
    dyson_df['simulated'] = simulation_data['simulated'].values

    # Calculate error (RMSE)
    error = np.sqrt(np.mean((dyson_df['simulated'] - dyson_df['actual heating energy']) ** 2))

    # Print error information after evaluation
    print(f"\nEvaluation completed, RMSE error: {error}")

    # Save results to a CSV file
    modified_csv_path = os.path.join(sim_output_dir, 'final_dyson_with_simulated.csv')
    dyson_df.to_csv(modified_csv_path, index=True)
    print(f"Data processing completed. Results saved to {modified_csv_path}")

    return (error,)

def post_process(sim_output_dir):
    """
    Post-processing function for calculating monthly data and saving it to a new Excel file.

    Args:
        sim_output_dir (str): Path to the simulation output directory.
    """
    # Define input and output file paths
    input_csv_path = os.path.join(sim_output_dir, 'final_dyson_with_simulated.csv')
    output_excel_path = os.path.join(sim_output_dir, 'processed_results.xlsx')

    # Check if the input file exists
    if not os.path.exists(input_csv_path):
        print(f"Input file {input_csv_path} does not exist. Post-processing cannot proceed.")
        return

    try:
        # Read daily data
        daily_data = pd.read_csv(input_csv_path, parse_dates=True)

        # Check if the necessary columns exist in the data
        required_columns = ['actual heating energy', 'simulated']
        if not all(col in daily_data.columns for col in required_columns):
            print(f"Input file is missing required columns: {required_columns}")
            return

        # Add date-related columns
```

```
    daily_data['Date'] = pd.to_datetime(daily_data['Date'])
    daily_data['Month'] = daily_data['Date'].dt.to_period('M')

    # Aggregate data by month
    monthly_data = daily_data.groupby('Month')[['actual heating energy', 'simulated']].sum().reset_index()

    # Calculate daily and monthly metrics
    metrics = {}

    # Daily metrics calculation
    daily_rmse = ((daily_data['simulated'] - daily_data['actual heating energy']) ** 2).mean() ** 0.5
    daily_cvrmse = daily_rmse / daily_data['actual heating energy'].mean()
    daily_nmse = ((daily_data['simulated'] - daily_data['actual heating energy']) ** 2).mean() /
daily_data['actual heating energy'].var()
    daily_r2 = 1 - daily_nmse

    metrics['Daily Metrics'] = {
        'CVRMSE': daily_cvrmse,
        'R2': daily_r2,
        'NMSE': daily_nmse
    }

    # Monthly metrics calculation
    monthly_rmse = ((monthly_data['simulated'] - monthly_data['actual heating energy']) ** 2).mean() **
0.5
    monthly_cvrmse = monthly_rmse / monthly_data['actual heating energy'].mean()
    monthly_nmse = ((monthly_data['simulated'] - monthly_data['actual heating energy']) ** 2).mean() /
monthly_data['actual heating energy'].var()
    monthly_r2 = 1 - monthly_nmse

    metrics['Monthly Metrics'] = {
        'CVRMSE': monthly_cvrmse,
        'R2': monthly_r2,
        'NMSE': monthly_nmse
    }

    # Save results to Excel
    with pd.ExcelWriter(output_excel_path) as writer:
        daily_data.to_excel(writer, sheet_name='Daily Data', index=False)
        monthly_data.to_excel(writer, sheet_name='Monthly Data', index=False)

        # Save daily and monthly metrics
        metrics_df = pd.DataFrame.from_dict(metrics, orient='index')
        metrics_df.to_excel(writer, sheet_name='Metrics')
```

```
        print(f"Post-processing completed. Results have been saved to {output_excel_path}")

    except Exception as e:
        print(f"An error occurred during post-processing: {e}")

def main():
    population = toolbox.population(n=100)   # Population size
    NGEN = 20   # Number of generations
    CXPB = 0.5   # Crossover probability
    MUTPB = 0.2   # Mutation probability

    print("Starting evolution")

    # Initialize a list to store the best fitness value for each generation
    best_fitness_values = []

    # Evaluate the initial population
    fitnesses = list(map(toolbox.evaluate, population))
    for ind, fit in zip(population, fitnesses):
        ind.fitness.values = fit

    print(f"\nEvaluated {len(population)} individuals")

    # Collect the best fitness value from the initial population
    fits = [ind.fitness.values[0] for ind in population if np.isfinite(ind.fitness.values[0])]
    if fits:
        best_fitness = min(fits)
        best_fitness_values.append(best_fitness)
    else:
        best_fitness_values.append(float('inf'))

    # Start evolution
    for gen in range(NGEN):
        print("\n" + "=" * 60)
        print(f"Starting generation {gen + 1}")
        print("=" * 60)

        # Select the next generation individuals
        offspring = toolbox.select(population, len(population))
        offspring = list(map(toolbox.clone, offspring))   # Use the custom clone function

        # Crossover and mutation
        for child1, child2 in zip(offspring[::2], offspring[1::2]):
```

```
        if np.random.rand() < CXPB:
            toolbox.mate(child1, child2)
            # Ensure parameters are within range
            for i in range(len(child1)):
                if i == 0:   # Heating setpoint
                    child1[i] = np.clip(child1[i], *heating_setpoint_range)
                    child2[i] = np.clip(child2[i], *heating_setpoint_range)
                elif i == 1:   # Equipment power density
                    child1[i] = np.clip(child1[i], *equipment_power_density_range)
                    child2[i] = np.clip(child2[i], *equipment_power_density_range)
                elif i == 2:   # Lighting power density
                    child1[i] = np.clip(child1[i], *lighting_power_density_range)
                    child2[i] = np.clip(child2[i], *lighting_power_density_range)
                elif i == 3:   # Number of people
                    child1[i] = np.clip(child1[i], *number_of_people_range)
                    child2[i] = np.clip(child2[i], *number_of_people_range)
                elif i == 4:   # Infiltration rate
                    child1[i] = np.clip(child1[i], *infiltration_ach_range)
                    child2[i] = np.clip(child2[i], *infiltration_ach_range)
                elif i == 5:   # Boiler design water flow rate
                    child1[i] = np.clip(child1[i], *design_water_flow_rate_range)
                    child2[i] = np.clip(child2[i], *design_water_flow_rate_range)
                elif i == 6:   # Boiler minimum part-load ratio
                    child1[i] = np.clip(child1[i], *minimum_part_load_ratio_range)
                    child2[i] = np.clip(child2[i], *minimum_part_load_ratio_range)
                elif i == 7:   # Boiler maximum part-load ratio
                    child1[i] = np.clip(child1[i], *maximum_part_load_ratio_range)
                    child2[i] = np.clip(child2[i], *maximum_part_load_ratio_range)
                elif i == 8:   # Heating coil U-value times area
                    child1[i] = np.clip(child1[i], *ufactor_times_area_value_range)
                    child2[i] = np.clip(child2[i], *ufactor_times_area_value_range)
                elif i == 9:   # Heating coil maximum water flow rate
                    child1[i] = np.clip(child1[i], *maximum_water_flow_rate_range)
                    child2[i] = np.clip(child2[i], *maximum_water_flow_rate_range)
            del child1.fitness.values
            del child2.fitness.values

    for mutant in offspring:
        if np.random.rand() < MUTPB:
            toolbox.mutate(mutant)
            # Ensure parameters are within range
            for i in range(len(mutant)):
                if i == 0:   # Heating setpoint
                    mutant[i] = np.clip(mutant[i], *heating_setpoint_range)
```

```
                elif i == 1:   # Equipment power density
                    mutant[i] = np.clip(mutant[i], *equipment_power_density_range)
                elif i == 2:   # Lighting power density
                    mutant[i] = np.clip(mutant[i], *lighting_power_density_range)
                elif i == 3:   # Number of people
                    mutant[i] = np.clip(mutant[i], *number_of_people_range)
                elif i == 4:   # Infiltration rate
                    mutant[i] = np.clip(mutant[i], *infiltration_ach_range)
                elif i == 5:   # Boiler design water flow rate
                    mutant[i] = np.clip(mutant[i], *design_water_flow_rate_range)
                elif i == 6:   # Boiler minimum part-load ratio
                    mutant[i] = np.clip(mutant[i], *minimum_part_load_ratio_range)
                elif i == 7:   # Boiler maximum part-load ratio
                    mutant[i] = np.clip(mutant[i], *maximum_part_load_ratio_range)
                elif i == 8:   # Heating coil U-value times area
                    mutant[i] = np.clip(mutant[i], *ufactor_times_area_value_range)
                elif i == 9:   # Heating coil maximum water flow rate
                    mutant[i] = np.clip(mutant[i], *maximum_water_flow_rate_range)
            del mutant.fitness.values

    # Evaluate invalid individuals
    invalid_ind = [ind for ind in offspring if not ind.fitness.valid]
    print(f"\nEvaluating {len(invalid_ind)} individuals")
    fitnesses = map(toolbox.evaluate, invalid_ind)
    for ind, fit in zip(invalid_ind, fitnesses):
        ind.fitness.values = fit

    # Update the population
    population[:] = offspring

    # Collect fitness values
    fits = [ind.fitness.values[0] for ind in population if np.isfinite(ind.fitness.values[0])]

    if fits:
        best_fitness = min(fits)
        best_fitness_values.append(best_fitness)
        print("\nStatistics for this generation:")
        print(f"   Minimum: {best_fitness}")
        print(f"   Maximum: {max(fits)}")
        print(f"   Mean: {sum(fits) / len(fits)}")
        print(f"   Standard deviation: {np.std(fits)}")
    else:
        best_fitness_values.append(float('inf'))
        print("All individuals have invalid fitness values (inf).")
```

```
    print("\n" + "=" * 60)
    print("Evolution completed")
    print("=" * 60)

    best_ind = tools.selBest(population, 1)[0]
    heating_setpoint = best_ind[0]
    equipment_power_density = best_ind[1]
    lighting_power_density = best_ind[2]
    number_of_people = int(round(best_ind[3]))
    infiltration_ach = best_ind[4]
    design_water_flow_rate = best_ind[5]
    minimum_part_load_ratio = best_ind[6]
    maximum_part_load_ratio = best_ind[7]
    ufactor_times_area_value = best_ind[8]
    maximum_water_flow_rate = best_ind[9]

    best_param_str = f"hsp_{round(heating_setpoint, 2)}_epd_{round(equipment_power_density,
2)}_lpd_{round(lighting_power_density, 2)}_pn_{number_of_people}_ach_{round(infiltration_ach,
2)}_flow_{round(design_water_flow_rate, 4)}_minplr_{round(minimum_part_load_ratio,
2)}_maxplr_{round(maximum_part_load_ratio, 2)}_ua_{round(ufactor_times_area_value,
2)}_maxflow_{round(maximum_water_flow_rate, 4)}"

    print(f"\nBest parameter combination:")
    print(f"   Heating setpoint = {round(heating_setpoint, 2)} °C")
    print(f"   Equipment power density = {round(equipment_power_density, 2)} W/m²")
    print(f"   Lighting power density = {round(lighting_power_density, 2)} W/m²")
    print(f"   Number of people = {number_of_people}")
    print(f"   Infiltration rate = {round(infiltration_ach, 2)} ACH")
    print(f"   Boiler design water flow rate = {round(design_water_flow_rate, 4)} m³/s")
    print(f"   Boiler minimum part-load ratio = {round(minimum_part_load_ratio, 2)}")
    print(f"   Boiler maximum part-load ratio = {round(maximum_part_load_ratio, 2)}")
    print(f"   Heating coil U-value times area = {round(ufactor_times_area_value, 2)} W/K")
    print(f"   Heating coil maximum water flow rate = {round(maximum_water_flow_rate, 4)} m³/s")
    print(f"   RMSE = {best_ind.fitness.values[0]}")

    # Additional code: Save the best parameters to a text file
    best_params_file = os.path.join(output_directory, 'best_parameters.txt')
    with open(best_params_file, 'w') as f:
        f.write("Best parameter combination:\n")
        f.write(f"   Heating setpoint = {round(heating_setpoint, 2)} °C\n")
        f.write(f"   Equipment power density = {round(equipment_power_density, 2)} W/m²\n")
        f.write(f"   Lighting power density = {round(lighting_power_density, 2)} W/m²\n")
        f.write(f"   Number of people = {number_of_people}\n")
```

```
        f.write(f"    Infiltration rate = {round(infiltration_ach, 2)} ACH\n")
        f.write(f"    Boiler design water flow rate = {round(design_water_flow_rate, 4)} m³/s\n")
        f.write(f"    Boiler minimum part-load ratio = {round(minimum_part_load_ratio, 2)}\n")
        f.write(f"    Boiler maximum part-load ratio = {round(maximum_part_load_ratio, 2)}\n")
        f.write(f"    Heating coil U-value times area = {round(ufactor_times_area_value, 2)} W/K\n")
        f.write(f"    Heating coil maximum water flow rate = {round(maximum_water_flow_rate, 4)} m³/s\n")
        f.write(f"    RMSE = {best_ind.fitness.values[0]}\n")
    print(f"\nBest parameters saved to {best_params_file}")

    # Print the mapping of parameter combinations to simulation output directories
    print("\nMapping of parameter combinations to simulation output directories:")
    for k, v in parameter_output_dirs.items():
        print(f"    Parameter combination: {k}, Simulation output directory: {v}")

    # Retrieve the corresponding simulation output directory for the best parameters
    sim_output_dir = parameter_output_dirs.get(best_param_str)

    if sim_output_dir:
        # Call the post-processing function to handle the best individual's simulation results
        post_process(sim_output_dir)
    else:
        print("No simulation output directory found for the best parameter combination.")

    # Plot the loss curve
    generations = range(len(best_fitness_values))
    plt.figure(figsize=(10, 6))
    plt.plot(generations, best_fitness_values, marker='o')
    plt.title('Genetic Algorithm Loss Curve')
    plt.xlabel('Generation')
    plt.ylabel('RMSE of the Best Individual')
    plt.grid(True)
    plt.savefig(os.path.join(output_directory, 'loss_curve.png'))
    plt.show()
    print(f"\nLoss curve saved to {os.path.join(output_directory, 'loss_curve.png')}")

if __name__ == "__main__":
    main()
```

Data S5-Energy Retrofits

In this section, the agent automates the process of equipment replacement, output modification, and report generation, ensuring that new devices are properly integrated into the building model and that simulation results are analyzed and documented efficiently. The process culminates in the automated generation of comprehensive reports that include key retrofit outcomes, such as energy use, costs, and carbon tax comparisons, along with clear visualizations for easy interpretation.

Simulation reset

Equipment replacement

This step details the process of replacing existing HVAC equipment within the building energy model using the OpenStudio SDK. The Programmer agent is tasked with generating Python code that follows strict template guidelines to remove outdated devices and install new equipment as specified by the user. The operation involves four main actions: removing old equipment, accurately locating the target plant loop, creating the new device with all required parameters, and connecting it to the appropriate loop branch. This approach ensures code consistency, clarity, and reproducibility while accommodating custom equipment specifications such as type, capacity, and performance parameters. The example below demonstrates the full dialogue and resulting code for replacing a boiler with an air source heat pump.

Dialogue with Programmer agent for automated HVAC equipment replacement using OpenStudio SDK:

Agent: Programmer (Consensus, gpt-4)

User Prompt:

I want to replace the original boiler with an air source heat pump, with a capacity of 200,000 and a COP of 3.5.

System Prompt:

Users want to write code that complies with the OpenStudio SDK specification according to their specific needs, and replace the specified HVAC equipment with new models. The operation steps must strictly follow the example template:

1. Remove old devices: Call the correct API method to clear all original devices of the target type;

2. Positioning loop: Retrieve the target pipeline through an accurate loop type search method (e.g., hot water/chilled water loop) and verify its existence;

3. Create a new device: Instantiate a new device using the constructor of the corresponding device class, and set all required parameters and optional parameters as required by the API documentation;

4. Connect to the loop: Select the correct piping connection method according to the device type (e.g., add to the water supply/return branch).

Implementation requirements:

The code structure/comments/exception handling should be exactly the same as the example of replacing a boiler with a heat pump.

- All placeholders must be replaced with the actual class names, method names, and parameter values that exist in the OpenStudio SDK.

- Keep the variable naming logic of the original example (such as `hot_water_loop`/`heat_pump`) but need to adapt it to new device types.

- Prohibit any extension functions or code annotations that go beyond the example code pattern.

Example Response:

```
# remove all existing equipment of specified type
```

```
old_equipment = model.get[EQUIPMENT_TYPE]()
for item in old_equipment:
    item.remove()
# get target plant loop
target_loop = model.get[LOOP_TYPE]()[INDEX]   # INDEX should be 0 for first loop, etc.
if not target_loop:
    raise ValueError('[LOOP_NAME] not found!')
# create new equipment
new_equipment = openstudio.model.[NEW_EQUIPMENT_CONSTRUCTOR](model)
new_equipment.setName('[EQUIPMENT_NAME]')
new_equipment.set[PARAMETER_NAME]([VALUE])   # Repeat for all required parameters
# configure optional settings
if [CONDITION]:
    new_equipment.set[OPTIONAL_PARAMETER]([VALUE])
# connect to loop
target_loop.[CONNECTION_METHOD](new_equipment)   # addSupplyBranchForComponent or other
```

Answer:

```python
# remove all boilers
boilers = model.getBoilerHotWaters()
for boiler in boilers:
    boiler.remove()

# get hot water loop
hot_water_loop = model.getPlantLoops()[0]
if hot_water_loop == False:
    raise ValueError('hot water loop not found!')

# create ASHP
heat_pump = openstudio.model.HeatPumpPlantLoopEIRHeating(model)
heat_pump.setName('Air Source Heat Pump')
heat_pump.setReferenceCapacity(200000)
heat_pump.setLoadSideReferenceFlowRate(0.001)
heat_pump.setReferenceCoefficientofPerformance(3.5)
heat_pump.setMinimumPartLoadRatio(0.1)
heat_pump.setSizingFactor(1.25) # optional
heat_pump.setControlType('LeavingSetpointModulated')
hot_water_loop.addSupplyBranchForComponent(heat_pump)
```

Output modification

This step focuses on updating and synchronizing the model's output variables following equipment replacement in compliance with OpenStudio standards. The agent responsible for this task is the Programmer. Based on the completed operation (e.g., replacing a boiler with a heat pump), the agent first removes the output variables associated with the old equipment by precisely matching both device identifiers and variable names. Then, the

agent configures new monitoring variables for the updated device, ensuring strict adherence to the official naming conventions in the OSM variable library. All variables are bound to the new device instance and assigned an appropriate reporting frequency.

Dialogue with the Programmer agent for updating model output variables following equipment replacement:

Agent: Programmer (Consensus, gpt-4)

System Prompt:

Users want to write code that complies with OpenStudio specifications based on the completed equipment replacement operation to synchronize and update the model output variables. The operation must strictly follow the example mode of replacing a boiler with a heat pump:

Clean up old monitoring data: Traverse the output variables through getOutputVariables(), and use a double condition match (device identifier + variable name) to accurately locate and remove the original monitoring items of the replaced device;

Configure new device monitoring: Call the OutputVariable constructor to declare a new variable, which must strictly match the official naming of the OSM variable library. Bind the new device instance identifier through setKeyValue(), and use standard reporting frequency parameters (such as Hourly/Daily);

Code constraints: Keep the variable filtering logic, parameter setting sequence, and exception handling paradigm in the example, and prohibit adding any model modification code unrelated to output variables. All placeholders must be replaced with the real device names and variable names supported by OSM.

Example Response:

```
# remove original variables related to old equipment
output_vars = model.getOutputVariables()
for output_var in output_vars:
    if output_var.keyValue() == "[OLD_EQUIPMENT_IDENTIFIER]" and output_var.variableName() ==
"[OLD_VARIABLE_NAME]":
        output_var.remove()
# create new output variables for replaced equipment
new_output = openstudio.model.OutputVariable("[NEW_VARIABLE_NAME]", model)
new_output.setKeyValue("[NEW_EQUIPMENT_IDENTIFIER]")
new_output.setReportingFrequency("[REPORTING_FREQUENCY]")    # Hourly/Daily/Monthly etc
```

Answer:

```
# remove original variables
output_vars = model.getOutputVariables()
for output_var in output_vars:
    if output_var.keyValue() == "Hot Water Boiler" and output_var.variableName() == "Boiler Heating
Energy":
        output_var.remove()

# add variables
new_output = openstudio.model.OutputVariable("Heat Pump Electricity Energy", model)
new_output.setKeyValue("Air Source Heat Pump")
new_output.setReportingFrequency("Daily")
```

Process Execution

Report generation

This section presents the automated generation of comprehensive analysis reports for building energy simulation results using an intelligent agent. The Result Analyzer agent is designed to interpret user requests and produce Python code that reads simulation outputs, performs required calculations (such as total energy use, cost, and carbon tax before and after retrofit), and generates relevant charts and tables. All analytical results, visualizations, and textual explanations are programmatically compiled into a well-structured Word document, ensuring reproducibility and efficiency. The process adapts dynamically to the user's analysis targets and formatting needs, producing clear, targeted reports without extraneous functionality. The example below illustrates the agent's system prompt, user request, and the automatically generated code for comparing energy consumption, costs, and carbon taxes, and for visualizing daily and annual results in both numerical and graphical forms.

Dialogue with Result Analyzer agent for automated simulation result analysis and Word report generation:

Agent: Result Analyzer (Consensus, gpt-4)

User Prompt:

Please analyze and compare the total energy consumption, total operating cost, and total carbon tax of the building before and after the retrofit.

Generate daily comparison charts for cost and carbon tax, and provide an annual cost breakdown (including energy cost and carbon tax) in both numerical and graphical form.

System Prompt:

You are an advanced building energy analysis agent. Your task is to write Python code that analyzes the simulation results of the original and retrofitted models, whose file names and locations are fixed. All data is provided in specified simulation result files.

According to the user's prompt, identify and implement all requested analyses (such as total energy consumption, total cost, carbon emissions, daily comparisons, cost breakdown, carbon tax, etc.), and generate all corresponding charts and tables as specified.

All calculated results, tables, figures, and written explanations must be automatically compiled into a well-structured Word document using python-docx. The code must fully adapt to the specific analysis targets and visualizations requested by the user, and must not include any additional functions beyond those required. The final code should be complete, clearly organized, and ensure that all analysis, charts, and comments required by the user prompt are automatically inserted into the generated Word report.

Answer:

```
def parse_daily_data(csv_path):
    """Parse daily data (match timestamps exactly at 24:00:00)"""
    daily_values = []

    with open(csv_path, 'r', encoding='utf-8') as f:
        reader = csv.reader(f)
        headers = next(reader)   # Skip header row

        for row in reader:
            # Parse time format: ' 01/01    24:00:00'
            timestamp = row[0].strip()
            if timestamp.endswith("24:00:00"):
```

```
                try:
                    daily_value = float(row[1])   # Extract second column data
                    daily_values.append(daily_value)
                except (IndexError, ValueError) as e:
                    print(f"Warning: Failed to parse row {row}, error: {str(e)}")
    print(daily_values)
    return daily_values

def generate_charts(original_daily, retrofitted_daily):
    """Generate analysis charts and save as images"""
    plt.style.use('seaborn-v0_8')
    os.makedirs("analysis_charts", exist_ok=True)

    # Parameter settings
    gas_price = 0.059      # £/kWh
    elec_price = 0.274     # £/kWh
    gas_carbon = 0.184 * 300 / 1000
    elec_carbon = 0.2 * 300 / 1000

    # 3. Annual cost breakdown bar chart (improved version)
    gas_energy = sum(original_daily)/3.6e6 * gas_price
    gas_tax = sum(original_daily)/3.6e6 * gas_carbon
    elec_energy = sum(retrofitted_daily)/3.6e6 * elec_price
    elec_tax = sum(retrofitted_daily)/3.6e6 * elec_carbon

    plt.figure(figsize=(10, 6))
    bar_width = 0.6
    index = np.arange(2)

    # Use different color schemes for system types
    gas_color = '#FFA500'   # Orange
    elec_color = '#1E90FF' # Blue

    # Plot stacked bar chart
    bar1 = plt.bar(index[0], gas_energy, bar_width,
                   color=gas_color, label='Gas Energy Cost')
    bar2 = plt.bar(index[0], gas_tax, bar_width,
                   bottom=gas_energy, color='#FF4500', label='Gas Carbon Tax')

    bar3 = plt.bar(index[1], elec_energy, bar_width,
                   color=elec_color, label='Heat Pump Energy Cost')
    bar4 = plt.bar(index[1], elec_tax, bar_width,
                   bottom=elec_energy, color='#4169E1', label='Heat Pump Carbon Tax')
```

```
    # Professional style adjustment
    plt.title('Annual Cost Breakdown Comparison', fontsize=14, pad=20)
    plt.ylabel('Cost (£)', fontsize=12)
    plt.xticks(index, ['Boiler', 'Heat Pump'], fontsize=12)
    plt.yticks(fontsize=10)
    plt.grid(axis='y', linestyle='--', alpha=0.7)

    # Combine legends and optimize layout
    handles = [bar1, bar2, bar3, bar4]
    plt.legend(handles=handles,
              loc='upper right',
              fontsize=10,
              frameon=True,
              shadow=True,
              facecolor='white')

    plt.savefig('analysis_charts/cost_breakdown.png', dpi=300, bbox_inches='tight')
    plt.close()

def generate_word_report(original_sum, retrofitted_sum):
    """Generate a Word format analysis report"""
    doc = Document()

    # Add title
    doc.add_heading('Energy Retrofit Analysis Report', 0)

    # Calculate key metrics
    gas_kwh = original_sum / 3.6e6
    cost_gas = gas_kwh * 0.059
    carbon_gas = gas_kwh * 0.184 * 300 / 1000
    total_gas = cost_gas + carbon_gas

    elec_kwh = retrofitted_sum / 3.6e6
    cost_elec = elec_kwh * 0.274
    carbon_elec = elec_kwh * 0.2 * 300 / 1000
    total_elec = cost_elec + carbon_elec

    savings = total_gas - total_elec
    savings_percent = (savings / total_gas) * 100

    # Add text analysis
    doc.add_heading('Key Metrics', level=1)
    text = f"""
=== Original System (Boiler) ===
```

```
Total Gas Consumption: {gas_kwh:.2f} kWh
Energy Cost: £{cost_gas:.2f}
Carbon Tax: £{carbon_gas:.2f}
Total Cost: £{total_gas:.2f}

=== Retrofitted System (Heat Pump) ===
Total Electricity Consumption: {elec_kwh:.2f} kWh
Energy Cost: £{cost_elec:.2f}
Carbon Tax: £{carbon_elec:.2f}
Total Cost: £{total_elec:.2f}

Annual Total Savings: £{savings:.2f} ({savings_percent:.2f}%)
    """
    for line in text.strip().split('\n'):
        doc.add_paragraph(line.strip())

    # Add charts
    doc.add_heading('Daily Cost Comparison', level=1)
    doc.add_picture('analysis_charts/daily_cost.png', width=Inches(6.5))

    doc.add_heading('Daily Carbon Tax Comparison', level=1)
    doc.add_picture('analysis_charts/daily_carbon.png', width=Inches(6.5))

    doc.add_heading('Annual Cost Breakdown', level=1)
    doc.add_picture('analysis_charts/cost_breakdown.png', width=Inches(5))

    # Save document
    report_path = os.path.join("simulation_results", "analysis_report.docx")
    doc.save(report_path)
    print(f"Word report generated: {os.path.abspath(report_path)}")

# Modify main program logic
try:
    # Get daily data
    original_daily = parse_daily_data(os.path.join("simulation_results", "Openstudiomodel", "results",
"eplusout.csv"))
    retrofitted_daily = parse_daily_data(os.path.join("simulation_results", "retrofitted_model", "results",
"eplusout.csv"))

    # Calculate sums
    original_sum = sum(original_daily)
    retrofitted_sum = sum(retrofitted_daily)

    # Generate charts
```

```
    generate_charts(original_daily, retrofitted_daily)

    # Generate Word report
    generate_word_report(original_sum, retrofitted_sum)

except Exception as e:
    print("Analysis failed:", str(e))
```

Result comparison

This section provides sample outputs generated by the automated reporting workflow described in previous sections. By integrating the equipment replacement and report generation code, the system automatically produces a Word report containing key quantitative results and visualization charts. The following figures are direct screenshots from the generated report, demonstrating the clarity and informativeness of the outputs, as well as the consistency of the automated process for building retrofit analysis.

Data S6-Construction of the web application

The frontend of the web application was developed using the Vue.js framework, which provides a responsive client-side interface for user interaction. Vue.js was responsible for rendering the user interface, capturing user inputs, and communicating with the backend via RESTful API requests. The backend was implemented using Python's Flask framework, which exposed a set of API endpoints to handle various functional requests from the frontend. When users interact with the application, the frontend sends requests to the corresponding API routes, and the backend processes these requests and returns the results to the frontend for display. This architecture ensures a clear separation between the client and server, supports modular development, and enables efficient real-time communication between user operations and backend computation.

Data S7-The impact of different prompt styles on results

During the implementation of tasks, the language style of the user prompt significantly affects the retrieval results of the Information Retriever. To test this, we varied the prompts by altering the tone styles, introducing spelling mistakes, or adding extra words. For each variation, we conducted 5 sets of tests. The results show that these changes primarily influence the naming strategies of code variables, without affecting the program's execution logic or output. The following experiments demonstrate that the code's running results, even with different variable naming schemes due to varied prompt styles, remain fully consistent with the baseline version. Programmers, based on the information extracted from different prompts, generate executable code that meets the user's requirements, achieving a 100% correctness rate. Since the results obtained from different prompts show significant similarity, the following presents two typical cases.

Case 1

In Parameter configuration, users provided descriptions of the building's HVAC system components. In Case 1, we compared the effects of a natural language prompt with one written in a technical engineering style, modifying key terms and sentence structures. While these stylistic changes led to notable differences in the JSON information extracted in HVAC system composition extraction, both JSON outputs resulted in identical modeling code when used with the model library for code generation in System model development. In addition, in Component parameter assignment, the information retrieved is used to complete the code in System model development, and the same results are obtained. This demonstrates that if the HVAC system description is accurate and complete, the system can reliably extract the necessary information and generate consistent executable code, regardless of prompt style.

The prompt and answer of HVAC system composition extraction after modification:

Agent: Information Retriever (Consensus, gpt-4)

User Prompt:

STRUCTURAL COMPONENTS:

Primary heat transfer medium: Pressurized hot water

Circulation backbone: Hot Water Loop infrastructure

Flow control elements: Hot Water Nodes (zone distributors)

Hydraulic drivers: Hot Water Pumps (variable speed)

Thermal generation: High-efficiency Boiler unit

Control system: Setpoint Manager with PID logic

Pressure regulation: Dual bypass system (Supply/Demand Bypass Pipes)

Terminal units: Aluminum-finned Radiators

System Prompt:

Extract HVAC system composition from user requirement. Please provide the results in a JSON format.

Example Response:

JSON format

Answer (extracted_HVAC_composition.json):


```
{
    "HVAC System Composition": {
        "Primary heat transfer medium": "Pressurized hot water",
        "Circulation backbone": "Hot Water Loop infrastructure",
        "Flow control elements": "Hot Water Nodes (zone distributors)",
```

```
        "Hydraulic drivers": "Hot Water Pumps (variable speed)",
        "Thermal generation": "High-efficiency Boiler unit",
        "Control system": "Setpoint Manager with PID logic",
        "Pressure regulation": "Dual bypass system (Supply/Demand Bypass Pipes)",
        "Terminal units": "Aluminum-finned Radiators"
    }
}
```


The prompt and answer of System model development after the result of HVAC system composition extraction is changed:

Agent: Programmer (Consensus, gpt-4)

Files:

extracted_HVAC_composition.json

Model library.xlsx

System Prompt:

Using the equipment data provided in the JSON file, identify the corresponding model code snippets for each HVAC component (e.g., boilers, pumps, radiators) from the model library. Combine these code snippets to generate a complete system equipment model in Python, ensuring that all components are integrated. The output should be a single, well-structured Python script representing the entire HVAC system.

Example Response:

Create hot water loop

CREATE a plant loop for hot water in the model

SET the name of the plant loop

Hot Water Node

GET the supply outlet node of the hot water plant

GET the supply inlet node of the hot water plant

Other component

...

Answer:

```python
# Hot Water Loop
hot_water_plant = openstudio.model.PlantLoop(model)
hot_water_plant.setName("Hot Water Loop")
for thermal_zone in model.getThermalZones():
    sizing_zone = openstudio.model.SizingZone(model, thermal_zone)
    sizing_zone.setZoneHeatingDesignSupplyAirTemperature(actual numeric value)
    sizing_zone.setHeatingDesignAirFlowMethod("None")
sizing_plant = hot_water_plant.sizingPlant()
sizing_plant.setLoopType("Heating")
sizing_plant.setDesignLoopExitTemperature(actual numeric value)
sizing_plant.setLoopDesignTemperatureDifference(actual numeric value)

# Hot Water Node
```

```python
hot_water_supply_outlet_node = hot_water_plant.supplyOutletNode()
hot_water_supply_inlet_node = hot_water_plant.supplyInletNode()

# Hot Water Pump
hot_water_pump = openstudio.model.PumpVariableSpeed(model)
hot_water_pump.setName("Hot Water Loop Pump")
hot_water_pump.addToNode(hot_water_supply_inlet_node)

# Boiler
boiler = openstudio.model.BoilerHotWater(model)
boiler.setName("Hot Water Boiler")
boiler.setBoilerFlowMode("LeavingSetpointModulated")
boiler.setNominalThermalEfficiency(actual numeric value)
boiler.setWaterOutletUpperTemperatureLimit(actual numeric value)
boiler.setSizingFactor(actual numeric value)
hot_water_plant.addSupplyBranchForComponent(boiler)
boiler.setNominalCapacity(actual numeric value)
boiler.setDesignWaterFlowRate(actual numeric value)
boiler.setMinimumPartLoadRatio(actual numeric value)
boiler.setMaximumPartLoadRatio(actual numeric value)

# Setpoint Manager
setpoint_manager = openstudio.model.SetpointManagerScheduled(model, hot_water_temp_schedule)
setpoint_manager.setControlVariable("Temperature")
setpoint_manager.addToNode(hot_water_supply_outlet_node)

# Supply Bypass Pipe
supply_bypass_pipe = openstudio.model.PipeAdiabatic(model)
hot_water_plant.addSupplyBranchForComponent(supply_bypass_pipe)

# Demand Bypass Pipe
demand_bypass_pipe = openstudio.model.PipeAdiabatic(model)
hot_water_plant.addDemandBranchForComponent(demand_bypass_pipe)

# Radiator
for thermal_zone in model.getThermalZones():
    heating_coil = openstudio.model.CoilHeatingWaterBaseboard(model)
    heating_coil.setName(f"Heating Coil for {thermal_zone.name().get()}")
    heating_coil.setMaximumWaterFlowRate(actual numeric value)
    heating_coil.setUFactorTimesAreaValue(actual numeric value)
    radiator = openstudio.model.ZoneHVACBaseboardConvectiveWater(model, radiator_schedule,
heating_coil)
    radiator.setName(f"Radiator in {thermal_zone.name().get()}")
    radiator.addToThermalZone(thermal_zone)
```

```
    hot_water_plant.addDemandBranchForComponent(heating_coil)
```

The prompt and answer of Component parameter assignment after the result of HVAC system composition extraction is changed:

Agent: Programmer (Consensus, gpt-4)

Files:

extracted_parameter.json

Code for System model development.py

System Prompt:

Using the extracted parameter values from json document, replace the placeholders marked as actual numeric value in the Code for System model development.py file with the corresponding real values.

Answer:

```
# Hot Water Loop
hot_water_plant = openstudio.model.PlantLoop(model)
hot_water_plant.setName("Hot Water Loop")
for thermal_zone in model.getThermalZones():
    sizing_zone = openstudio.model.SizingZone(model, thermal_zone)
    sizing_zone.setZoneHeatingDesignSupplyAirTemperature(24)
    sizing_zone.setHeatingDesignAirFlowMethod("None")
sizing_plant = hot_water_plant.sizingPlant()
sizing_plant.setLoopType("Heating")
sizing_plant.setDesignLoopExitTemperature(75)
sizing_plant.setLoopDesignTemperatureDifference(10)

# Hot Water Node
hot_water_supply_outlet_node = hot_water_plant.supplyOutletNode()
hot_water_supply_inlet_node = hot_water_plant.supplyInletNode()

# Hot Water Pump
hot_water_pump = openstudio.model.PumpVariableSpeed(model)
hot_water_pump.setName("Hot Water Loop Pump")
hot_water_pump.addToNode(hot_water_supply_inlet_node)

# Boiler
boiler = openstudio.model.BoilerHotWater(model)
boiler.setName("Hot Water Boiler")
boiler.setBoilerFlowMode("LeavingSetpointModulated")
boiler.setNominalThermalEfficiency(1)
boiler.setWaterOutletUpperTemperatureLimit(45)
boiler.setSizingFactor(1.25)
hot_water_plant.addSupplyBranchForComponent(boiler)
boiler.setNominalCapacity(240000)
boiler.setDesignWaterFlowRate(0.005)
boiler.setMinimumPartLoadRatio(0.15)
```

```python
boiler.setMaximumPartLoadRatio(0.6)

# Setpoint Manager
setpoint_manager = openstudio.model.SetpointManagerScheduled(model, hot_water_temp_schedule)
setpoint_manager.setControlVariable("Temperature")
setpoint_manager.addToNode(hot_water_supply_outlet_node)

# Supply Bypass Pipe
supply_bypass_pipe = openstudio.model.PipeAdiabatic(model)
hot_water_plant.addSupplyBranchForComponent(supply_bypass_pipe)

# Demand Bypass Pipe
demand_bypass_pipe = openstudio.model.PipeAdiabatic(model)
hot_water_plant.addDemandBranchForComponent(demand_bypass_pipe)

# Radiator
for thermal_zone in model.getThermalZones():
    heating_coil = openstudio.model.CoilHeatingWaterBaseboard(model)
    heating_coil.setName(f"Heating Coil for {thermal_zone.name().get()}")
    heating_coil.setMaximumWaterFlowRate(0.001)
    heating_coil.setUFactorTimesAreaValue(500)
    radiator = openstudio.model.ZoneHVACBaseboardConvectiveWater(model, radiator_schedule,
heating_coil)
    radiator.setName(f"Radiator in {thermal_zone.name().get()}")
    radiator.addToThermalZone(thermal_zone)
    hot_water_plant.addDemandBranchForComponent(heating_coil)
```

Case 2

In this case, we modified the user prompt in Calibration parameters analysis, altering how certain calibration parameters were described. This change in expression style led to corresponding differences in the variable names used in the code generated by the Information Retriever and Programmer agents, as reflected in Parameter range assignment, Model initialization, Measure application, and Best offspring record.

The prompt and answer of Calibration parameters analysis after modification:

Agent: Information Retriever (Consensus, gpt-4)

User Prompt:

We propose to designate the following parameters as key identification variables, within their respective operational ranges:

Temperature Settings for Heating Systems: The temperature is maintained between 18°C and 24°C.

Power Density of Equipment: The power density ranges from 3 W/m² to 22 W/m².

Power Density of Lighting Systems: The lighting power density is set between 1.5 W/m² and 15 W/m².

Occupancy Levels: Occupancy is adjusted from 30 to 200 people, based on spatial dimensions and office configurations.

Hourly Air Change Rate for Infiltration (ACH): The ACH is calibrated between 0.5 and 1.5, according to environmental conditions.

Boiler Design Water Flow Rate: The water flow rate for the boiler ranges from 0.0001 m^3/s to 0.005 m^3/s.

Boiler Minimum and Maximum Operational Capacity Ratios: The minimum load ratio is between 0.1 and 0.4, while the maximum load ratio spans from 0.6 to 0.9.

Heat Transfer Coefficient-Area Product for Heating Coils: This value is set between 500.0 W/K and 2000.0 W/K.

Peak Water Flow Capacity for Heating Coils: The maximum water flow rate for the heating coils is between 0.001 m^3/s and 0.006 m^3/s.

System Prompt:

Extract information on calibration parameters and their ranges from User requirement. Please provide the results in a JSON format.

Example Response:

JSON format

Answer:


```json
{
    "calibration_parameters": [
        {
            "parameter": "temperature settings for heating systems",
            "range": {
                "min": 18,
                "max": 24,
                "unit": "°C"
            }
        },
        {
            "parameter": "power density of equipment",
            "range": {
                "min": 3,
                "max": 22,
                "unit": "W/m²"
            }
        },
        {
            "parameter": "power density of lighting systems",
            "range": {
                "min": 1.5,
                "max": 15,
                "unit": "W/m²"
            }
        },
        {
            "parameter": "occupancy levels",
            "range": {
```

```
                "min": 30,
                "max": 200,
                "unit": "persons"
            }
        },
        {
            "parameter": "hourly air change rate for infiltration",
            "range": {
                "min": 0.5,
                "max": 1.5,
                "unit": "ACH"
            }
        },
        {
            "parameter": "design water flow rate for boilers",
            "range": {
                "min": 0.0001,
                "max": 0.005,
                "unit": "m³/s"
            }
        },
        {
            "parameter": "minimum operational capacity ratio for boilers",
            "range": {
                "min": 0.1,
                "max": 0.4,
                "unit": ""
            }
        },
        {
            "parameter": "maximum operational capacity ratio for boilers",
            "range": {
                "min": 0.6,
                "max": 0.9,
                "unit": ""
            }
        },
        {
            "parameter": "heat transfer coefficient-area product for heating coils",
            "range": {
                "min": 500.0,
                "max": 2000.0,
                "unit": "W/K"
            }
```

```
            },
            {
                "parameter": "peak water flow capacity for heating coils",
                "range": {
                    "min": 0.001,
                    "max": 0.006,
                    "unit": "m³/s"
                }
            }
        ]
}
```


The prompt and answer of Parameter range assignment after the result of Calibration parameters analysis is changed：

Agent: Programmer (Consensus, gpt-4)

Files:

extracted_calibration_parameter_information.json

System Prompt:

Using the extracted parameter ranges from json document, write the code according to the example response. Do not load any file. Do not define the model library in the code. Do not import required python libraries. Don't write iterations.

Example Response:

parameter_name_range = (lower_bound_value, upper_bound_value)

Answer:

```
# Define the parameter ranges
temperature_settings_for_heating_systems_range = (18, 24)
power_density_of_equipment_range = (3, 22)
power_density_of_lighting_systems_range = (1.5, 15)
occupancy_levels_range = (30, 200)
hourly_air_change_rate_for_infiltration_range = (0.5, 1.5)
design_water_flow_rate_for_boilers_range = (0.0001, 0.005)
minimum_operational_capacity_ratio_for_boilers_range = (0.1, 0.4)
maximum_operational_capacity_ratio_for_boilers_range = (0.6, 0.9)
heat_transfer_coefficient_area_product_for_heating_coils_range = (500.0, 2000.0)
peak_water_flow_capacity_for_heating_coils_range = (0.001, 0.006)
```

The prompt and answer of Model initialization after the result of Calibration parameters analysis is changed:

Agent: Programmer (Consensus, gpt-4)

Files:

extracted_calibration_parameter_information.json

System Prompt:

Using the calibration parameter data provided in the JSON file, replace the placeholders in the example response with the corresponding real names. Only replace the parts involving the names, do not delete or

modify my other code. Do not load any file. Do not import required python libraries. Do not assume the JSON content is stored in a variable. Make sure all mentioned calibration parameters are included in the code. Do not define calibration parameters!!! Please list all the involved code line by line, do not write it as a iteration, and directly include the calibration parameter names in the code without defining variables.

Example Response:

calibration_parameter_1 = individual[0]

calibration_parameter_2 = individual[1]

calibration_parameter_3 = individual[2]

...

sim_output_dir = join the output directory path with a generated unique simulation identifier

IF the simulation output directory does not exist:

    CREATE the simulation output directory

param_str = (

    f'calibration_parameter_1_' with its formatted value

    f'calibration_parameter_2_' with its formatted value

    f'calibration_parameter_3_' with its formatted value

    ...

)

Answer:

```
temperature_settings_for_heating_systems = individual[0]
power_density_of_equipment = individual[1]
power_density_of_lighting_systems = individual[2]
occupancy_levels = individual[3]
hourly_air_change_rate_for_infiltration = individual[4]
design_water_flow_rate_for_boilers = individual[5]
minimum_operational_capacity_ratio_for_boilers = individual[6]
maximum_operational_capacity_ratio_for_boilers = individual[7]
heat_transfer_coefficient_area_product_for_heating_coils = individual[8]
peak_water_flow_capacity_for_heating_coils = individual[9]

sim_output_dir = os.path.join(output_directory, f'sim_{uuid.uuid4().hex}')
if not os.path.exists(sim_output_dir):
    os.makedirs(sim_output_dir)

param_str = (
    f'temperature_settings_for_heating_systems_{round(temperature_settings_for_heating_systems, 2)}_'
    f'power_density_of_equipment_{round(power_density_of_equipment, 2)}_'
    f'power_density_of_lighting_systems_{round(power_density_of_lighting_systems, 2)}_'
    f'occupancy_levels_{round(occupancy_levels, 2)}_'
    f'hourly_air_change_rate_for_infiltration_{round(hourly_air_change_rate_for_infiltration, 2)}_'
    f'design_water_flow_rate_for_boilers_{round(design_water_flow_rate_for_boilers, 2)}_'
```

```
f'minimum_operational_capacity_ratio_for_boilers_{round(minimum_operational_capacity_ratio_for_boilers, 2)}_'

f'maximum_operational_capacity_ratio_for_boilers_{round(maximum_operational_capacity_ratio_for_boilers, 2)}_'

f'heat_transfer_coefficient_area_product_for_heating_coils_{round(heat_transfer_coefficient_area_product_for_heating_coils, 2)}_'
    f'peak_water_flow_capacity_for_heating_coils_{round(peak_water_flow_capacity_for_heating_coils, 2)}_'
)
```

The prompt and answer of Measure application after the result of Calibration parameters analysis is changed:
Agent: Programmer (Consensus, gpt-4)

Files:

extracted_calibration_parameter_information.json

System Prompt:

Using the calibration parameter data provided in the JSON file, select the corresponding measure code snippets for each measure from the model library. Combine these code snippets to generate a complete code, ensuring that all measures are integrated. Please directly take the measure code from the model library, without modifying or writing iterations. Do not load any file. Do not define the extracted calibration parameter content. Do not define the model library in the code. Do not import required python libraries.

Example Response:

CREATE an instance of ExampleMeasure

GET the arguments for ExampleMeasure using the model

CREATE an argument map for ExampleMeasure

CLONE the required argument for ExampleMeasure

SET the value of the cloned argument using the corresponding calibration parameter

ASSIGN the cloned argument to the argument map with the correct key

APPEND the tuple of (ExampleMeasure, argument map) to the list of measures

Answer:

```
runner = openstudio.measure.OSRunner(openstudio.WorkflowJSON())
measures = []

# Replacing placeholders in the example response with the corresponding real names from the JSON file

measure_hsp = AdjustHeatingSetpointMeasure()
args_hsp = measure_hsp.arguments(model)
arg_map_hsp = openstudio.measure.OSArgumentMap()
hsp_arg = args_hsp[0].clone()
hsp_arg.setValue(temperature_settings_for_heating_systems)
arg_map_hsp["heating_setpoint"] = hsp_arg
```

```
measures.append((measure_hsp, arg_map_hsp))

measure_epd = AdjustEquipmentPowerDensityMeasure()
args_epd = measure_epd.arguments(model)
arg_map_epd = openstudio.measure.OSArgumentMap()
epd_arg = args_epd[0].clone()
epd_arg.setValue(power_density_of_equipment)
arg_map_epd["epd_value"] = epd_arg
measures.append((measure_epd, arg_map_epd))

measure_lpd = AdjustLightingPowerDensityMeasure()
args_lpd = measure_lpd.arguments(model)
arg_map_lpd = openstudio.measure.OSArgumentMap()
lpd_arg = args_lpd[0].clone()
lpd_arg.setValue(power_density_of_lighting_systems)
arg_map_lpd["lpd_value"] = lpd_arg
measures.append((measure_lpd, arg_map_lpd))

measure_pn = AdjustPeopleNumberMeasure()
args_pn = measure_pn.arguments(model)
arg_map_pn = openstudio.measure.OSArgumentMap()
number_of_people_arg = args_pn[0].clone()
number_of_people_arg.setValue(occupancy_levels)
arg_map_pn["number_of_people"] = number_of_people_arg
measures.append((measure_pn, arg_map_pn))

measure_infiltration = AdjustInfiltrationRateMeasure()
args_infiltration = measure_infiltration.arguments(model)
arg_map_infiltration = openstudio.measure.OSArgumentMap()
infiltration_ach_arg = args_infiltration[0].clone()
infiltration_ach_arg.setValue(hourly_air_change_rate_for_infiltration)
arg_map_infiltration["ach_value"] = infiltration_ach_arg
measures.append((measure_infiltration, arg_map_infiltration))

measure_boiler_flow = AdjustBoilerDesignWaterFlowRateMeasure()
args_boiler_flow = measure_boiler_flow.arguments(model)
arg_map_boiler_flow = openstudio.measure.OSArgumentMap()
design_water_flow_rate_arg = args_boiler_flow[0].clone()
design_water_flow_rate_arg.setValue(design_water_flow_rate_for_boilers)
arg_map_boiler_flow["design_water_flow_rate"] = design_water_flow_rate_arg
measures.append((measure_boiler_flow, arg_map_boiler_flow))

measure_boiler_min_plr = AdjustBoilerMinimumPartLoadRatioMeasure()
args_boiler_min_plr = measure_boiler_min_plr.arguments(model)
```

```
arg_map_boiler_min_plr = openstudio.measure.OSArgumentMap()
min_plr_arg = args_boiler_min_plr[0].clone()
min_plr_arg.setValue(minimum_operational_capacity_ratio_for_boilers)
arg_map_boiler_min_plr["minimum_part_load_ratio"] = min_plr_arg
measures.append((measure_boiler_min_plr, arg_map_boiler_min_plr))

measure_boiler_max_plr = AdjustBoilerMaximumPartLoadRatioMeasure()
args_boiler_max_plr = measure_boiler_max_plr.arguments(model)
arg_map_boiler_max_plr = openstudio.measure.OSArgumentMap()
max_plr_arg = args_boiler_max_plr[0].clone()
max_plr_arg.setValue(maximum_operational_capacity_ratio_for_boilers)
arg_map_boiler_max_plr["maximum_part_load_ratio"] = max_plr_arg
measures.append((measure_boiler_max_plr, arg_map_boiler_max_plr))

measure_coil_ua = AdjustHeatingCoilUFactorTimesAreaMeasure()
args_coil_ua = measure_coil_ua.arguments(model)
arg_map_coil_ua = openstudio.measure.OSArgumentMap()
ua_value_arg = args_coil_ua[0].clone()
ua_value_arg.setValue(heat_transfer_coefficient_area_product_for_heating_coils)
arg_map_coil_ua["ufactor_times_area_value"] = ua_value_arg
measures.append((measure_coil_ua, arg_map_coil_ua))

measure_coil_max_flow = AdjustHeatingCoilMaximumWaterFlowRateMeasure()
args_coil_max_flow = measure_coil_max_flow.arguments(model)
arg_map_coil_max_flow = openstudio.measure.OSArgumentMap()
max_water_flow_rate_arg = args_coil_max_flow[0].clone()
max_water_flow_rate_arg.setValue(peak_water_flow_capacity_for_heating_coils)
arg_map_coil_max_flow["maximum_water_flow_rate"] = max_water_flow_rate_arg
measures.append((measure_coil_max_flow, arg_map_coil_max_flow))

for measure, arg_map in measures:
    measure.run(model, runner, arg_map)
    result = runner.result()
```

The prompt and answer of Best offspring record after the result of Calibration parameters analysis is changed:
Agent: Programmer (Consensus, gpt-4)

Files:

extracted_calibration_parameter_information.json

System Prompt:

Using the calibration parameter data provided in the JSON file, replace the placeholders in the example response with the corresponding real names. Only replace the parts involving the names, do not delete or modify my other code. Do not load any file. Do not import required python libraries. Do not assume the JSON content is stored in a variable. Make sure all mentioned calibration parameters are included in the code. Do

not define calibration parameters!!! Please list all the involved code line by line, do not write it as an iteration, and directly include the calibration parameter names in the code without defining variables.

Example Response:

```
best_ind = select the best individual from the population

calibration_parameter_1 = best_ind[0]
calibration_parameter_2 = best_ind[1]
calibration_parameter_3 = best_ind[2]
...
calibration_parameter_N = best_ind[N]

best_param_str = (
    f"calibration_parameter_1_" with the formatted value
    f"calibration_parameter_2_" with the formatted value
    f"calibration_parameter_3_" with the formatted value
    ...
    f"calibration_parameter_N_" with the formatted value
)

best_params_file = join the output directory path with 'best_parameters.txt'
with open(best_params_file, 'w') as file_handle:
    write "Best Parameter Set:\n" to the file
    write the formatted value of calibration_parameter_1 to the file
    write the formatted value of calibration_parameter_2 to the file
    write the formatted value of calibration_parameter_3 to the file
    ...
    write the formatted value of calibration_parameter_N to the file
```

Answer:

```
best_ind = tools.selBest(population, 1)[0]
temperature_settings_for_heating_systems = best_ind[0]
power_density_of_equipment = best_ind[1]
power_density_of_lighting_systems = best_ind[2]
occupancy_levels = best_ind[3]
hourly_air_change_rate_for_infiltration = best_ind[4]
design_water_flow_rate_for_boilers = best_ind[5]
minimum_operational_capacity_ratio_for_boilers = best_ind[6]
maximum_operational_capacity_ratio_for_boilers = best_ind[7]
heat_transfer_coefficient_area_product_for_heating_coils = best_ind[8]
peak_water_flow_capacity_for_heating_coils = best_ind[9]

best_param_str = (
    f"hsp_{round(temperature_settings_for_heating_systems, 2)}_"
```

```
    f"epd_{round(power_density_of_equipment, 2)}_"
    f"lpd_{round(power_density_of_lighting_systems, 2)}_"
    f"ol_{round(occupancy_levels, 2)}_"
    f"acr_{round(hourly_air_change_rate_for_infiltration, 2)}_"
    f"dwfr_{round(design_water_flow_rate_for_boilers, 6)}_"
    f"mocr_{round(minimum_operational_capacity_ratio_for_boilers, 2)}_"
    f"maxocr_{round(maximum_operational_capacity_ratio_for_boilers, 2)}_"
    f"htcap_{round(heat_transfer_coefficient_area_product_for_heating_coils, 2)}_"
    f"pwfc_{round(peak_water_flow_capacity_for_heating_coils, 3)}_"
)

best_params_file = os.path.join(output_directory, 'best_parameters.txt')
with open(best_params_file, 'w') as f:
    f.write("Best Parameter Set:\n")
    f.write(f"    temperature settings for heating systems = {round(temperature_settings_for_heating_systems,
2)} °C\n")
    f.write(f"    power density of equipment = {round(power_density_of_equipment, 2)} W/m²\n")
    f.write(f"    power density of lighting systems = {round(power_density_of_lighting_systems, 2)} W/m²\n")
    f.write(f"    occupancy levels = {round(occupancy_levels, 2)} persons\n")
    f.write(f"    hourly air change rate for infiltration = {round(hourly_air_change_rate_for_infiltration, 2)}
ACH\n")
    f.write(f"    design water flow rate for boilers = {round(design_water_flow_rate_for_boilers, 6)} m³/s\n")
    f.write(f"    minimum operational capacity ratio for boilers =
{round(minimum_operational_capacity_ratio_for_boilers, 2)}\n")
    f.write(f"    maximum operational capacity ratio for boilers =
{round(maximum_operational_capacity_ratio_for_boilers, 2)}\n")
    f.write(f"    heat transfer coefficient-area product for heating coils =
{round(heat_transfer_coefficient_area_product_for_heating_coils, 2)} W/K\n")
    f.write(f"    peak water flow capacity for heating coils =
{round(peak_water_flow_capacity_for_heating_coils, 3)} m³/s\n")

sim_output_dir = parameter_output_dirs.get(best_param_str)
```
